# Hand-arm vibration and the risk of vascular and neurological diseases—A systematic review and meta-analysis

**DOI:** 10.1371/journal.pone.0180795

**Published:** 2017-07-13

**Authors:** Tohr Nilsson, Jens Wahlström, Lage Burström

**Affiliations:** Occupational and Environmental Medicine, Department of Public Health & Clinical Medicine, Umeå University, Umeå, Sweden; McMaster University, CANADA

## Abstract

**Background:**

Increased occurrence of Raynaud’s phenomenon, neurosensory injury and carpal tunnel syndrome has been reported for more than 100 years in association with work with vibrating machines. The current risk prediction modelling (ISO-5349) for “Raynaud’s phenomenon” is based on a few studies published 70 to 40 years ago. There are no corresponding risk prediction models for neurosensory injury or carpal tunnel syndrome, nor any systematic reviews comprising a statistical synthesis (meta-analysis) of the evidence.

**Objectives:**

Our aim was to provide a systematic review of the literature on the association between Raynaud’s phenomenon, neurosensory injuries and carpal tunnel syndrome and hand-arm vibration (HAV) exposure. Moreover the aim was to estimate the magnitude of such an association using meta-analysis.

**Methods:**

This systematic review covers the scientific literature up to January 2016. The databases used for the literature search were PubMed and Science Direct. We found a total of 4,335 abstracts, which were read and whose validity was assessed according to pre-established criteria. 294 articles were examined in their entirety to determine whether each article met the inclusion criteria. The possible risk of bias was assessed for each article. 52 articles finally met the pre-established criteria for inclusion in the systematic review.

**Results:**

The results show that workers who are exposed to HAV have an increased risk of vascular and neurological diseases compared to non-vibration exposed groups. The crude estimate of the risk increase is approximately 4–5 fold. The estimated effect size (odds ratio) is 6.9 for the studies of Raynaud’s phenomenon when including only the studies judged to have a low risk of bias. The corresponding risk of neurosensory injury is 7.4 and the equivalent of carpal tunnel syndrome is 2.9.

**Conclusion:**

At equal exposures, neurosensory injury occurs with a 3-time factor shorter latency than Raynaud’s phenomenon. Which is why preventive measures should address this vibration health hazard with greater attention.

## Introduction

Extensive and prolonged exposure to manual work involving the use of vibrating power tools can lead to a number of adverse health effects, primarily in the peripheral neurological, vascular and musculoskeletal systems [1, 2]. The resulting symptom complex is now collectively summarised and internationally acknowledged as hand-arm-vibration syndrome (HAVS).

The vascular component of HAVS represents an increased tendency to vasospasm in the digital capillaries, and is manifested by the appearance of “white finger” (Raynaud’s Phenomenon). The neurological component includes both a diffuse peripheral neurosensory injury and an entrapment of the median nerve at the wrist, entailing a symptom complex covered by the carpal tunnel syndrome (CTS) concept. In both neurosensory cases, symptoms of disturbed neural function include positive, negative and provokable manifestations [3]. Skeletal injuries manifest as osteoarthritis. An increased occurrence of reduced muscular function and the development of tendinopathies, tenosynovitis or fibrosis (e.g. Dupytrens contracture) are also reported.

The evidence of the relation between vibration exposure and injury is currently based solely on descriptive, narrative summaries of scientific reports. Only for “Raynaud’s phenomenon” is there a risk assessment model, as presented in the annexe to ISO 5349–1 [4]. Neurosensory injury, carpal tunnel syndrome and musculoskeletal injuries all lack separate ISO-standard models for risk assessment in relation to vibration exposure.

Increased occurrence of Raynaud’s phenomenon has been reported for more than 100 years. These findings come from studies of several work environments where exposure to vibration was present [5–7]. The conclusion that the symptoms were caused by exposure to vibrations and that the symptoms could be triggered by cold was not confirmed until the 1930s in connection with studies of foundry workers [8]. Alternate concurrent exposures (e.g. cold, quartz exposure) may also contribute to the occurrence of vascular damage and trigger the symptoms. It is well documented that work involving vibration exposure represents a potential risk for “Raynaud’s phenomenon”, but little is known about the extent to which the exposure to vibration in itself contributes to “Raynaud’s phenomenon”. Current risk prediction modelling for “Raynaud’s phenomenon” (ISO 5349) is based on estimates of prevalence rates and latencies from seven studies published during the period from 1946 to 1977 [9]. The studies included represent relatively high intensity exposures measured in the dominant vibration direction (A(8) within the domain 9–20 m/s^2^) and long working hours, of 9–12 hours pro day. The model assumptions on exposure were conditioned that the exposure to vibrations was more or less continuous, with only minor, brief interruptions. For the low daily exposure predictions, the risk estimates were extrapolated based on the relationships that were found for high exposures. The prevalence rates of “Raynaud’s phenomenon” in the underlying studies varied from 30% to 70%. The relationship was, moreover based on definitions of “Raynaud’s phenomenon” that varied in the degree of diagnostic resolution and precision.

Our aim was to provide a systematic review of the literature on the association between “Raynaud’s phenomenon” (“white finger”) and hand-arm vibration exposure with special attention paid to exposure estimates and the risk of bias. Moreover, the aim was to add a complementary statistical synthesis by estimating the magnitude of such an association using meta-analysis.

Reports witness neurosensory injuries in the form of sensory impairment, reduced motor function and trouble from neurosensory symptoms in relation to vibration exposure, over a long period. The reports stem from work environments where vibration exposure was prevalent [6]. The nerve injuries have to varying degrees been interpreted as a general manifestation of a widespread syndrome to specific nerve dysfunctions. The neurosensory symptoms were until the mid-1900s attributed to early signs of the development of vascular injury. The current knowledge of neurosensory injuries in relation to vibration exposure is insufficient to indicate to what extent the exposure to vibration in itself contributes to the ‘neuro-sensory impairment “, as distinguished from other work exposures.

Our aim was also to provide a systematic review of the literature on the association between neurosensory disorders and hand-arm vibration exposure with special attention paid to exposure estimates and the risk of bias. Moreover, the aim was to estimate the magnitude of such an association using meta-analysis

Nerve symptoms in accordance with carpal tunnel syndrome were reported early in studies from specific work environments in which vibrating tools were used [6]. A relation between work with vibrating tools and the contracting of carpal tunnel syndrome is supported by several separate original studies and systematic reviews (e.g. [10] and [11]). Most of the original reports and reviews on the relation between CTS and vibration exposure use job title as an indicator of exposure to vibration. A restricted number of studies present the total operating time (TOT) but there is a general lack of information on the vibration acceleration levels.

There is currently a shortage of knowledge about the extent to which exposure to vibration in itself contributes to “carpal tunnel syndrome”, as distinct from other ergonomic work exposures.

The following systematic review with accompanying meta-analysis attempts to answer the question of the relationship between the risks of “carpal tunnel syndrome” and exposure to hand-transmitted vibration with special attention being paid to exposure estimates and the risk of bias.

## Method

This systematic review with supporting statistical syntheses (meta-analyses) is limited to hand-arm vibration exposure and vascular and nerve damage. We accumulate nerve damage as neurosensory damage and as carpal tunnel syndrome. The hand-arm vibration syndrome’s vascular and nervous manifestations can occur either separately or together and without mutual relation. The following systematic review will discuss the different outcomes separately. Only studies in which a measurement or an estimation of vibration exposure has been reported are included in this review.

### Systematic literature search

Our systematic review follows the “PRISMA statement” for reporting systematic reviews and meta-analyses [12]. We adhere to Prisma’s guidelines regarding the application of its 24-point checklist, terminology, flow and reporting. The systematic literature review was based solely on original scientific papers published in refereed journals.

The databases used for interrogation were PubMed (US National Library of Medicine, Bethesda, Maryland) and Science Direct (Elsevier, Amsterdam). The reason for the slightly overlapping databases was that these databases index articles from partially various journals. The search strategy has deliberately been as broad as possible so as to include articles with HAV exposure and the outcome of malfunctioning of vessels and nerves in the hand-arm (in the form of either blood flow disorders (Raynaud’s phenomenon), the effect on nerves (neuro-sensory impairment) or specific nerve effects on the median nerve in the wrist (carpal tunnel syndrome). Initial search was carried out on a broad basis, without selection criteria based on indexing. Articles published in languages other than English and studies that did not involve effects on humans were excluded manually. The detailed search string is presented in the supporting information (S1 Text), and the literature search covers publications from 1945 until January 1, 2016.

The articles’ abstracts were examined in relation to the purpose of the literature review with requirements for information on vibration exposure as well as current health outcomes. All articles were reviewed by two of the authors independently. In case of disagreement, all three authors discussed each article until a consensus was reached.

For each article abstract which was found to be relevant, the full article was read by two reviewers independently to ensure that the relevant criteria were met. The reviewers used a protocol with defined quality with points (S1 Table), which focused on the study’s methodology and scientific quality in the sense of risk of bias. Each article was discussed by the two reviewers and in case of disagreement all three reviewers discussed it until there was a consensus.

**Fig 1:**
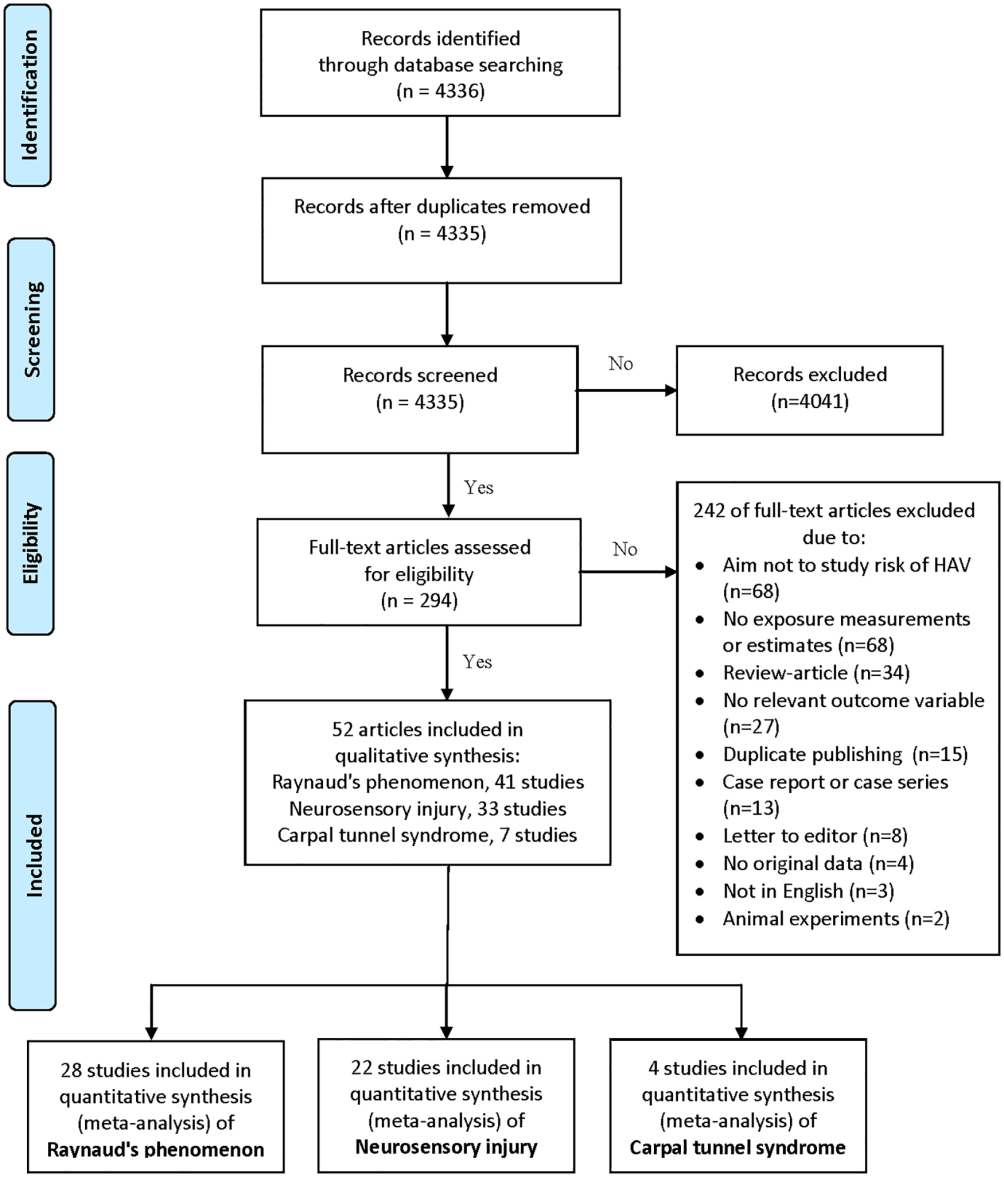
Flow chart of the search strategy and selection of studies in order to evaluate the relationship between the hand-arm vibration and health outcomes in accordance with the PRISMA (Raynaud’s phenomenon, neurosensory injury, carpal tunnel syndrome).

We found a total of 4,335 abstracts (Fig 1), which were read and whose validity was assessed according to pre-established criteria. 294 articles were then examined in their entirety to determine whether each article met the inclusion criteria. From the 293 articles 242 were excluded (Fig 1), which resulted in 52 articles finally meeting the pre-established criteria for inclusion in the systematic review.

### Meta-analysis

Studies that reported a relative risk (odds ratio) for any of the three outcomes (Raynaud’s phenomenon, neurosensory damage and CTS) were included in our meta-analyses. Included in the meta-analysis were only studies that presented data which enabled us to calculate an un-adjusted odds ratio.

In order to study the influence of the magnitude of the vibration exposure on the outcome, studies of the same groups of vibration-exposed workers, but with varying exposure levels, were gathered. The lowest exposure group in each study was defined as “Low exposure” and the highest exposure group as “High exposure”. The comparisons of estimates have been made in accordance with Altman and Bland [13].

Exposure quantification was based on the exposure data that could be identified in each article. In the articles where information could be found, the daily equivalent vibration expressed as A(8), has been calculated. The following four doses have been identified: Dose 1 = Number of exposure years (year); Dose 2 = Number of exposure hours (h); Dose 3 = Daily vibration exposure, A (8) (m/s^2^); Dose 4 = Cumulative vibration exposure (mh/s^2^, m^2^h/s^4^ or m^2^h^3^/s^4^).

We carried out “random-effect” meta-analyses and tested the heterogeneity between the different studies using the Cochran chi-square (Q-test) and I^2^ statistics [14, 15]. The risk of bias was assessed by cumulative meta-analysis and by subgroup analysis. For cumulative meta-analysis, studies were ranked in descending order by their respective graded quality score (risk of bias). The results are reported for each study effect size as odds ratios with 95% confidence intervals (lower and upper limit) and the z-value as well as p-value.

Publication bias was examined with funnel plots. Asymmetry in funnel plots were assessed by three statistical methods: the rank correlation method [16], regression analysis [17] and the Duval and Tweedie “trim and fill” method [18]. In the case of empty cells occurring (for vascular injury only), we have added one case to the empty cells, and a case in the other cells to allow the calculation of risk estimates [19].

Of the original 52 articles, only have 24 been included in our meta-analyses (Fig 1). The exclusion is conditioned, among other things, by the fact that certain articles only described the prevalence of injury in vibration-exposed individuals (9 articles omitted) [20–28] or that the aim was to compare exposed with unexposed individuals regarding any specific clinical outcome for example temperature thresholds (6 articles omitted) ([29–34]). Studies were also omitted that describe the same study population and have similar data (2 articles omitted) [35, 36]. In these cases, only the publication that we in the consensus deemed most relevant has been included in the meta-analyses.

All calculations were made using the statistical program CMA Comprehensive Meta-Analysis version 3.3 (Biostat, Englewood, United States; [37])

## Results

### Raynaud’s phenomenon

The result of the systematic literature review presented in Table 1 shows both our estimate of risk of bias (S1 Table) regarding the reliability of the diagnosis for “Raynaud’s phenomenon” and the total number of quality points. The total is the sum of the quality scores for the diagnosis added to the quality score for “study method” and “exposure”.

**Table 1:** Includes studies of Raynaud’s phenomenon and their assessed risk of bias (quality score) regarding the diagnosis “Raynaud’s phenomenon” (Diagnosis sum) and the total sum when the quality score for assessing diagnosis’ Raynaud’s phenomenon, “study method” and “exposure” has been added (Total score). The studies are presented in descending order based on the total score. Higher scores indicate higher “quality”, indicating a smaller possible risk of bias. Furthermore, the study design is given for each study.

Study	Reference	Design	Diagnosis sum	Total sum
Bovenzi, 1998	[35]	Cohort	11	29
Bovenzi, 2008	[36]	Cohort	11	28
Bovenzi, 2008*	[38]	Cohort	11	27
Bovenzi, 2000	[39]	Case-control	11	26
Bovenzi, 2010*	[40]	Cohort	9	26
Bovenzi, 1995*	[41]	Cross-section	11	25
Bovenzi, 2010*	[42]	Cohort	9	25
Bovenzi, 1998*	[43]	Cross-section	11	24
Mirbod, 1992*	[44]	Case-control	11	23
Bovenzi, 2011*	[45]	Cohort	8	23
Mirbod, 1999	[27]	Cohort	8	22
Aiba, 2012	[20]	Cohort	8	21
Hagberg, 2008*	[46]	Cohort	4	21
Barregard, 2003	[21]	Case-control	8	20
Bovenzi, 1988*	[47]	Cross-section	10	19
Bovenzi, 1985	[48]	Cross-section	9	19
Bovenzi, 1994*	[49]	Cross-section	8	19
Nilsson, 1989*	[50]	Cross-section	7	19
Palmer, 1998*	[51]	Cross-section	7	18
Futatsuka, 2005*	[52]	Cross-section	7	17
Brubaker, 1987*	[22]	Cohort	6	17
Koskimies, 1992	[26]	Cross-section	6	17
Futatsuka, 1985	[23]	Cohort	2	17
Su, 2013	[53]	Cross-section	6	16
Jang, 2002*	[54]	Cross-section	4	16
Yamada, 1995*	[55]	Cross-section	9	15
Bovenzi, 2005*	[56]	Cross-section	6	15
Letz, 1992*	[57]	Cross-section	4	15
Chatterjee, 1978*	[58]	Cross-section	6	14
Harazin, 1996	[25]	Cross-section	6	14
Brubaker, 1985*	[59]	Cross-section	5	14
Burdorf, 1991*	[60]	Cross-section	3	14
Cherniack, 2004*	[61]	Cross-section	3	14
Anttonen, 1994*	[62]	Cross-section	1	12
Virokannas, 1995*	[63]	Cross-section	1	12
Bovenzi, 1980*	[64]	Cross-section	2	10
Musson, 1989	[28]	Cross-section	1	10
Walker, 1985*	[65]	Cross-section	1	9
Burstrom, 2010*	[66]	Cross-section	1	8
Mirbod, 1994*	[67]	Cross-section	1	7
Tominaga, 1994*	[68]	Cross-section	1	7

*The study included in the meta-analysis

**Fig 2:**
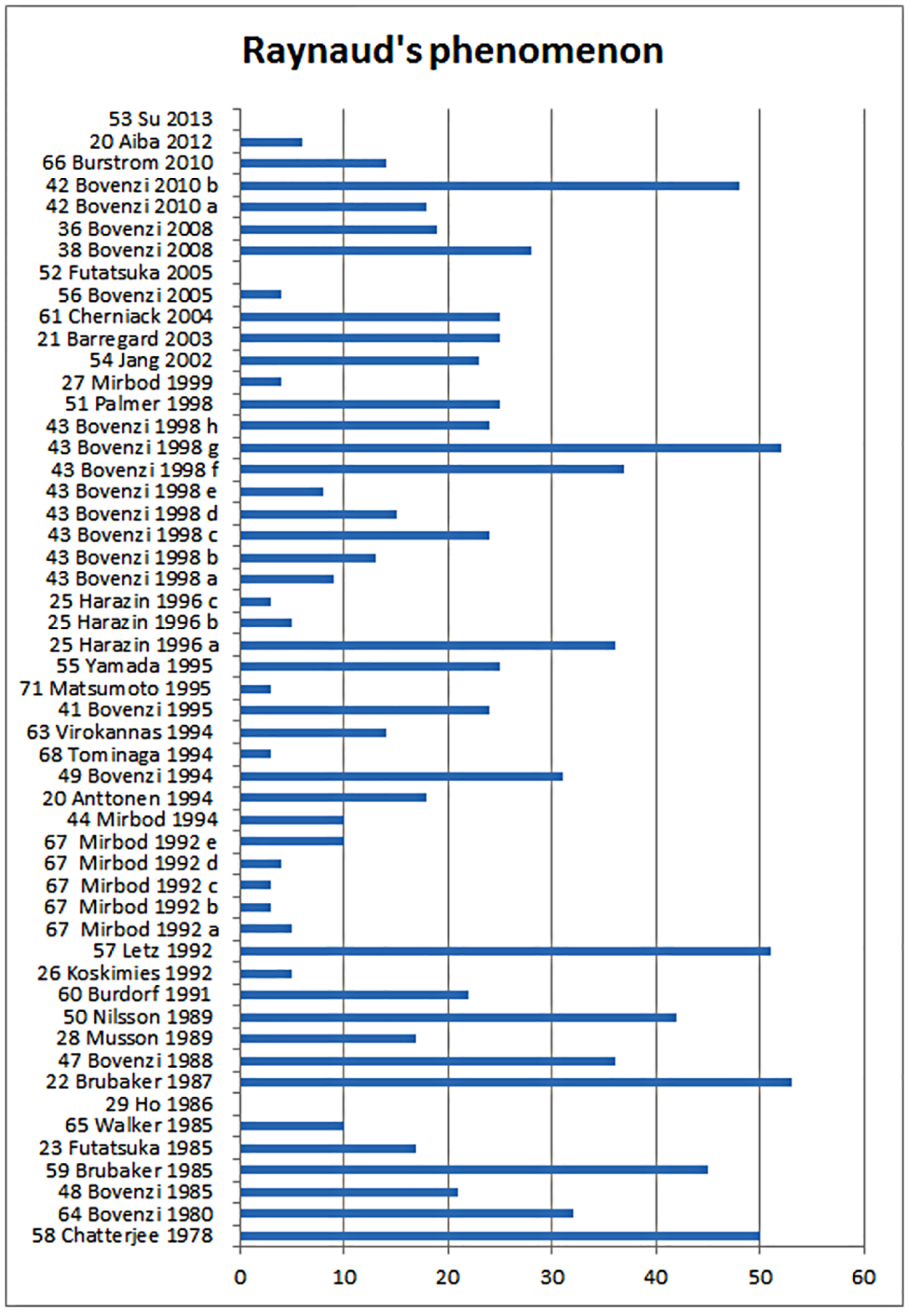
The prevalence of Raynaud’s phenomenon in the included studies sorted by year of publication.

For the outcome Raynaud’s phenomenon 41 articles were obtained (Table 1), published between 1978 and 2013. The articles examined had a variation in their mean score of between 1 and 11 for the quality points for the diagnosis of Raynaud’s phenomenon. The prevalence of Raynaud’s phenomenon varies from 0% to 53% in the different studies, with an average of 22% (Fig 2).

**Fig 3:**
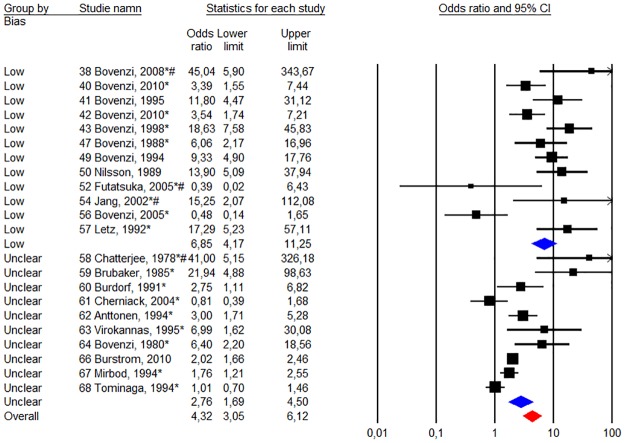
Statistics and forest plot of a “random - effect” meta-analysis of the prevalence of Raynaud’s phenomenon between the groups exposed to HAV and non-exposed reference groups. The size of the square of the individual studies is proportional to the study’s weight in the analysis. The red diamond indicates the overall risk. The studies have been ranked in order from highest to lowest quality score points according to quality criteria in appendix (S1 Table) and Table 1. The asterisk indicates that the study data presented made it possible to calculate the unadjusted odds ratio.

The total weighted risk of all studies for Raynaud’s phenomenon gave an odds ratio of 4.56 (95% CI 3.00–6.95). Fig 3 depicts on a forest plot the results of our meta-analysis on studies when we compare the risk of Raynaud’s phenomenon between groups exposed to HAV versus unexposed reference groups.

Stratified meta-analysis of studies with low risk of bias (studies with the highest quality scores in Table 1) versus those with unclear / high risk of bias (studies with a minimum grade point) shows large differences. The group of studies with a low risk of bias (n = 12) reported an overall odds ratio of 6.85 (95% CI 4.17–11.25), while group of studies with unclear / high risk (n = 10) for bias had an overall odds ratio of 2.76 (95% CI 1.69–4.50). Heterogeneity was comparable between both groups of studies [75% (p <0.01) vs. 81% (p <0.01)].

Stratified studies based on the year of publication (before and after 1997; n = 13 vs. n = 9) noted that the weighting of the older studies provide a higher odds ratio compared to recent studies [6.03 (p <0.01) Vs. 3.13 (p <0:01)], while heterogeneity is comparable [88% (p <0.01) Vs. 85% (p <0.01)]. The analysis was stratified in studies with a low risk of bias (based on the distribution of quality points according to S1 Table) versus those with an unclear risk of bias. Within the two risk groups (n = 12 and n = 10), studies have been ranked in descending order from those with the lowest risk of bias to those with the highest risk, based on their overall quality score.

**Fig 4:**
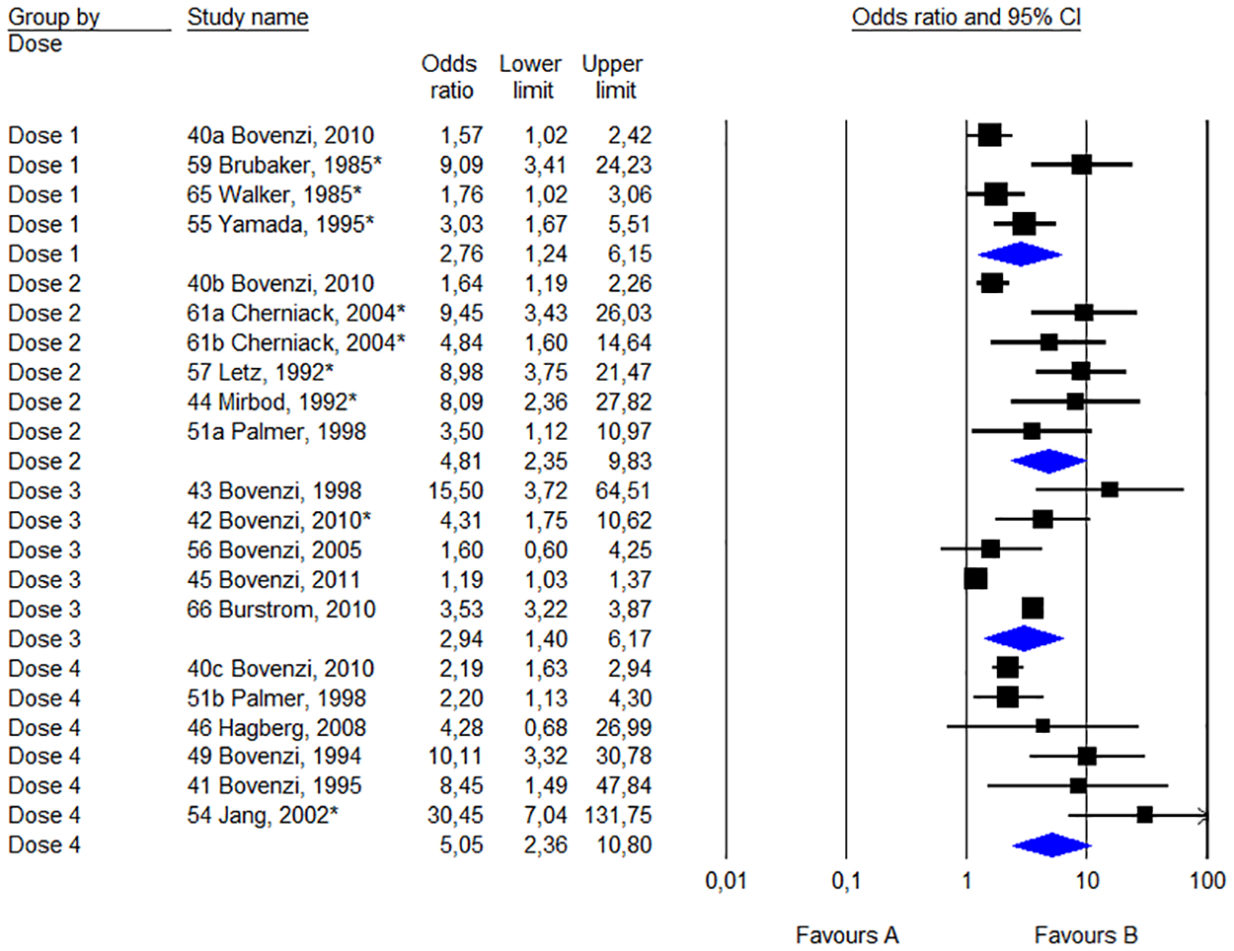
Statistics and forest plot of the weighting for each dose studies in Raynaud’s phenomenon sorted by various measuring cup. The size of the square of the individual studies is proportional to the study’s importance in the analysis. The blue diamond’s (rhomboids) shows the combined effect of the subgroups of measuring cup; Dose 1 = Number of exposure years (year); Dose 2 = Number of exposure hours (h); Dose 3 = Daily vibration exposure, A (8) (m/s2); Dose 4 = Cumulative vibration exposure (mh/s^2^, m^2^h/s^4^ or m^2^h^3^/s^4^). The studies have been ranked in order from highest to lowest quality score points according to Table 1. The asterisk indicates that the study data presented made it possible to calculate the unadjusted odds ratio.

Fig 4 describes the results of the meta-analysis for studies comparing the risk of Raynaud’s phenomenon between groups exposed to different levels of HAV. The analysis has been divided between the four different categories of measuring doses. For each measuring dose, studies have been ranked in descending order based on their total score (Table 1). In some of the articles several exposure parameters have been presented.

For the different dose measures varies the odds ratios between 2.64 (p <0.01) and 5.08 (p <0.01). The heterogeneity of the various dose measures is 75% (p <0.01), 81% (p <0.01), 98% (p <0.01) and 75% (p <0.01).

**Fig 5:**
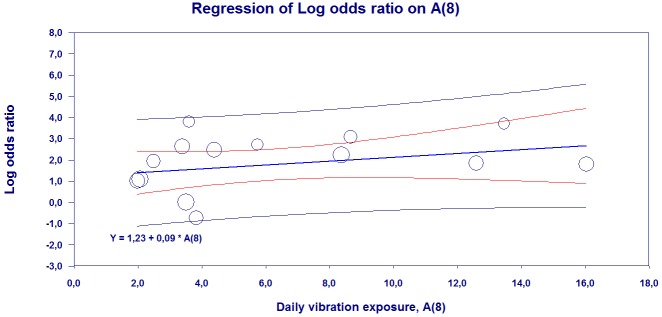
Meta-Regression analysis of the relationship between the logarithm of the prevalence of Raynaud’s phenomenon in vibration-exposed and vibration exposure A(8) (random effect; n = 14). The figure also shows the regression line’s 95% confidence intervals intervals (red lines). Blue lines show the prediction interval. The size of the circles represents the importance of the study results in the estimates of the regression.

Fig 5 shows the results of the meta-regression analysis of the relationship between A(8) and the natural logarithm of the prevalence of Raynaud’s phenomenon among vibration-exposed individuals. The result is based on the data of 33 studies, and in some of the studies several groups of exposed been present.

The analysis shows that the relation is reliable (p = 0.012) and the coefficient of A(8) is 0.09. The results also show that there is a significant (p <0.001) variation between the studies, indicating that, although the A (8) is the same in the two studies, prevalence of Raynaud’s phenomenon varies. The model explains 16% of variation between studies.

**Fig 6:**
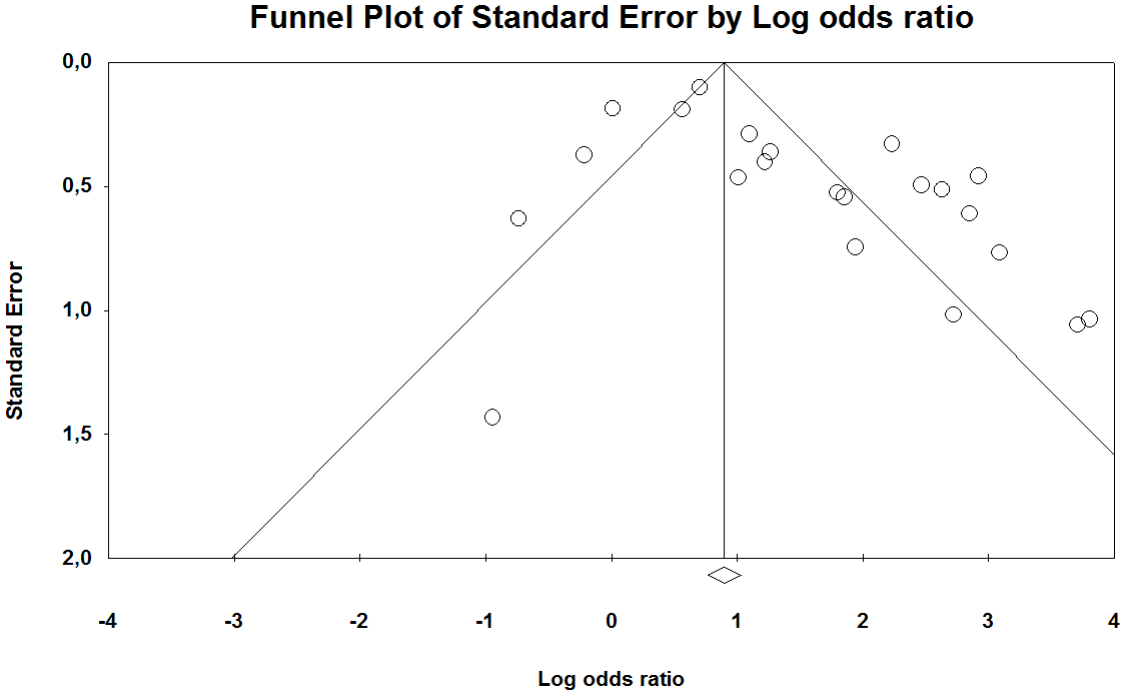
Funnel plot with pseudo 95% confidence interval for publication bias in studies of the association between the occurrence of Raynaud’s phenomenon among groups exposed to HAV and non-exposed reference groups. Beggs test shows no evidence of publication bias (p = 0.14), while Eggers test indicates such an effect (p <0.01). The trim and fill method imputed three missing studies to the left of the mean (random-effects model).

**Fig 7:**
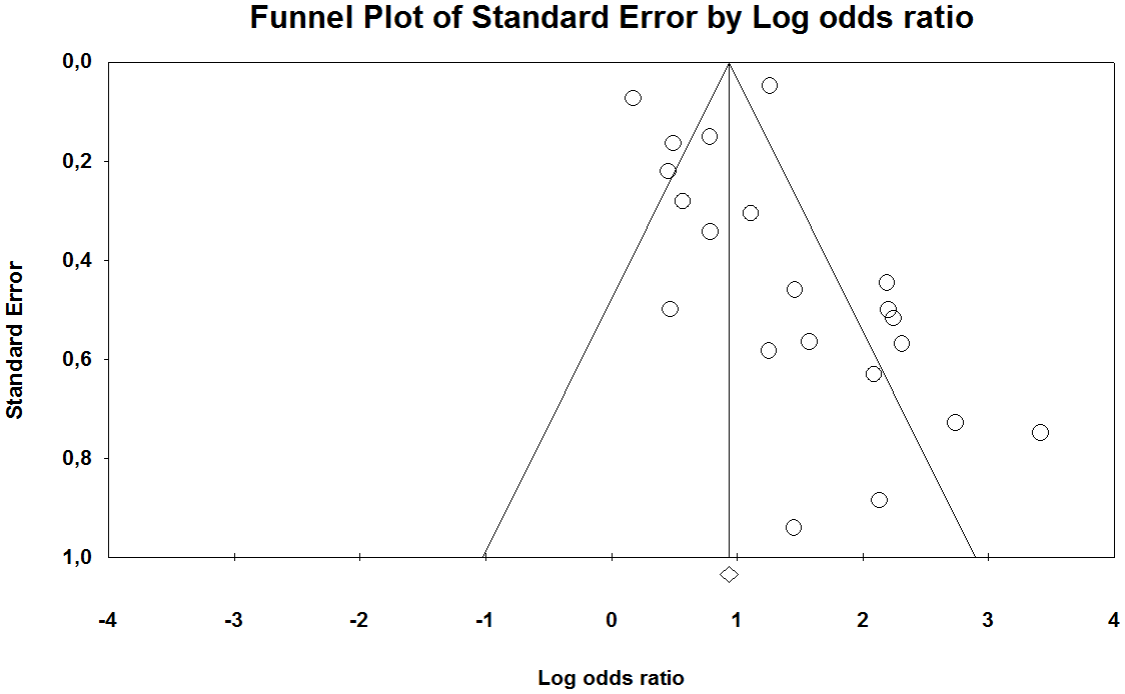
Funnel l plot with pseudo 95% confidence interval for publication bias in studies of the association between the occurrences of Raynaud’s phenomenon among groups exposed to different levels of HAV. Beggs test (p = 0.04), but not Eggers test (p = 0.36), showed evidence of publication bias and trim and fill method imputed seven studies lacked the left of the mean (random-effect model).

The funnel plot of the studies included in our meta-analysis indicates that they were distributed symmetrically around the estimated effect, suggesting little effect of publication bias for the contrast between HAV-exposed and unexposed individuals (Fig 6). However, between the low and high exposed group there was a tendency that the studies were distributed somewhat asymmetrically around the estimated effect, indicating some publication bias (Fig 7).

### Neuro-sensory injury

The results of the systematic literature review on articles addressing neurosensory injuries are given in Table 2. The table shows both our estimate of risk of bias (S1 Table) regarding the reliability of the diagnosis of “neuro-sensory injury” as well as the total number of quality points. The total is the sum of the quality scores for the diagnosis added to the quality score for “study method” and “exposure”.

**Table 2:** Included studies of neuro-sensory injury and their assessed risk of bias (quality score) regarding the diagnosis “neuro-sensory injury “(Diagnosis sum) and the total sum when the quality score for assessing diagnosis’ neuro-sensory injury, “study method” and “exposure” has been added (Total score). The studies are presented in descending order based on the total score. Higher scores indicate higher “quality”, indicating less possible risk of bias. Furthermore, the study design is given for each study.

Study	Reference	Design	Diagnosis sum	Total sum
Bovenzi, 2011*	[69]	Cohort	11	25
Bovenzi, 2000	[39]	Case-control	9	24
Mirbod, 1992*	[44]	Case-control	11	23
Mirbod, 1999	[27]	Cohort	9	23
Nilsson, 2008	[31]	Cross-section	9	20
Barregard, 2003	[21]	Case-control	8	20
Nilsson, 2001	[33]	Cross-section	8	20
Ho, 1986	[29]	Cross-section	11	19
Su, 2013*	[53]	Cross-section	9	19
Bovenzi, 1994*	[49]	Cross-section	8	19
Futatsuka, 2005*	[52]	Cross-section	8	18
Edlund, 2013*	[70]	Cohort	2	18
Bovenzi, 1988*	[47]	Cross-section	8	17
Bovenzi, 1985*	[48]	Cross-section	7	17
Palmer, 1998*	[51]	Cross-section	6	17
Yamada, 1995	[55]	Cross-section	10	16
Lundstrom, 1999	[30]	Cross-section	6	16
Koskimies, 1992	[26]	Cross-section	5	16
Jang, 2002*	[54]	Cross-section	4	16
Matsumoto, 1995*	[71]	Cross-section	9	15
Gerhardsson, 2005	[24]	Cross-section	6	15
Letz, 1992*	[57]	Cross-section	4	15
Chatterjee, 1978*	[58]	Cross-section	6	14
Malchaire, 2001*	[72]	Cross-section	4	14
Burdorf, 1991*	[60]	Cross-section	3	14
Cherniack, 2004*	[61]	Cross-section	3	14
Brubaker, 1985*	[59]	Cross-section	4	13
Anttonen, 1994*	[62]	Cross-section	1	12
Virokannas, 1995*	[63]	Cross-section	1	12
Bovenzi, 2005*	[56]	Cross-section	2	11
Musson, 1989	[28]	Cross-section	1	10
Bovenzi, 1980*	[64]	Cross-section	1	9
Mirbod, 1994*	[67]	Cross-section	1	7

*The study included in the meta-analysis

**Fig 8:**
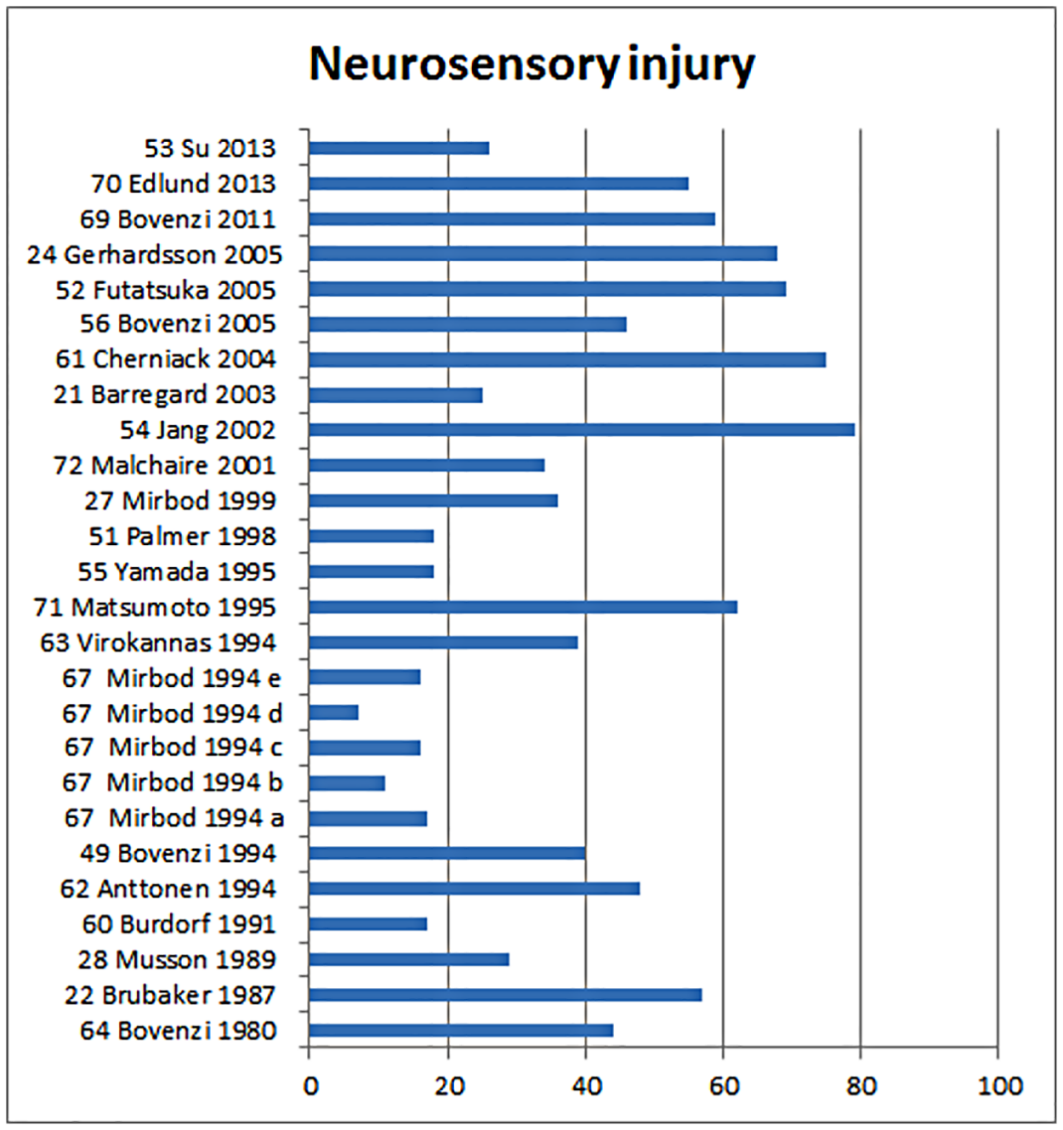
The prevalence of neuro-sensory injury in the studies included sorted by year of publication.

For the outcome neurosensory injury, 33 articles were included, of which 7 articles are unique to neurosensory injury. 21 articles included also Raynaud’s phenomenon and 2 articles carpal tunnel syndrome. For the outcome neurosensory damage articles included were published from 1978 to 2013. The prevalence of neurosensory damage has varied in the different studies from 17% to 79%, with an average of 43% (Fig 8).

**Fig 9:**
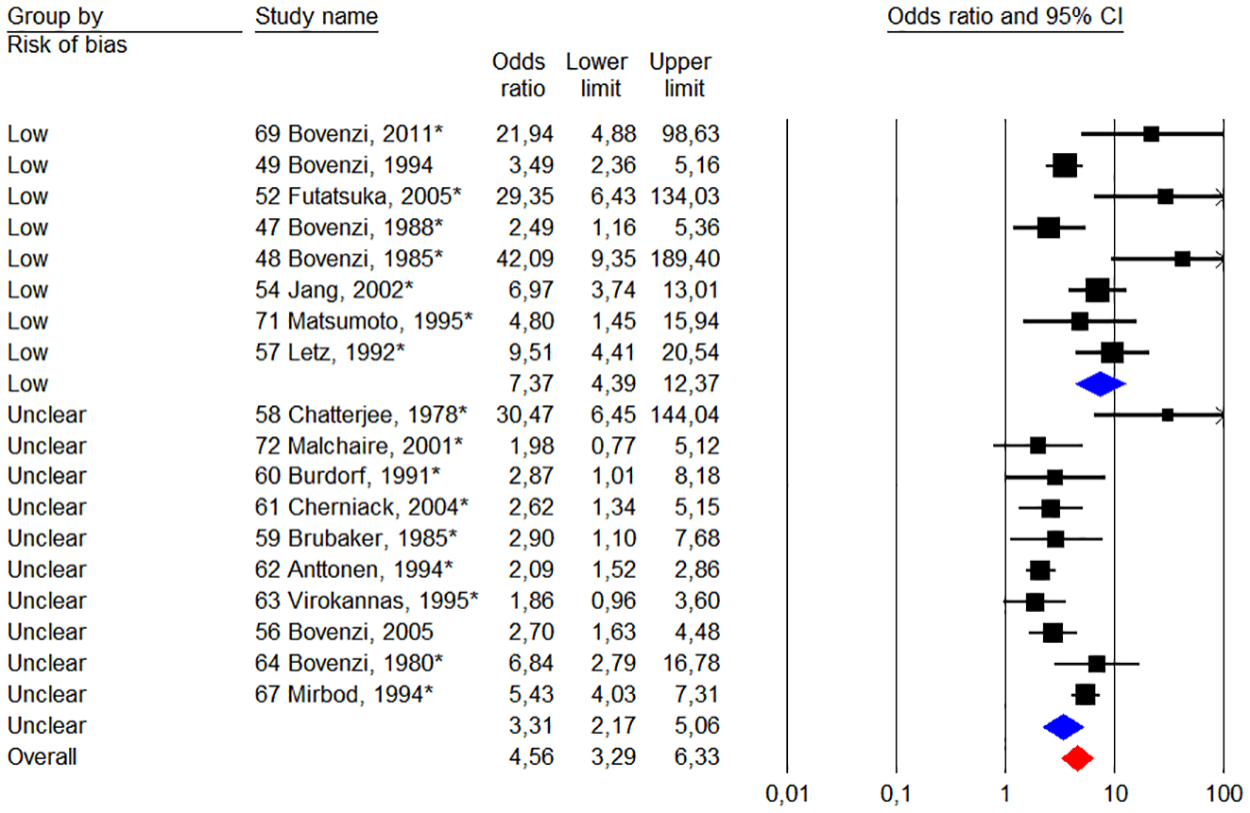
Statistics and “forest plot” with aggregate from “random - effect” meta-analysis of the incidence of neurosensory injury between groups exposed to HAV and non-exposed reference groups. The size of the squares of the individual studies is proportional to the importance of the study in the analysis. The red diamond represents the weighted risk for all studies. The studies have been sorted in order from highest to lowest quality score points according to Table 2. The asterisk indicates that the study data presented made it possible to calculate the unadjusted odds ratio.

The total weighted risk of all 18 studies in neuro-sensory injury gave an odds ratio of 4.58 (95% CI 3.28 to 6.38). Fig 9 describes in a forest plot the results of the meta-analysis of studies where we compare the risk of neurosensory injury between groups exposed to HAV versus unexposed reference group.

Stratified meta-analysis of studies with a low risk of bias (studies with the highest quality scores in Table 2) versus those with an unclear / high risk of bias (studies with the lowest quality score) shows some differences. The group of studies with a low risk of bias (n = 8) reported an overall odds ratio of 7.37 (95% CI 4.28 to 14.15), while the group of studies with an unclear / high risk (n = 10) for bias had an overall odds ratio of 3.31 (95% CI 2.17–5.06). Heterogeneity was the same for both groups of studies [74% (p <0:01) vs. 74% (p <0:01)].

Stratified meta-analysis on studies based on the year of publication (before resp. after 1997; n = 12 and n = 6) shows no significant difference in risk estimates between early and later publications [5.08 (p <0:01) vs. 4.46 (p <0:01)] and similar heterogeneity [76% (p <0.01) vs. 78% (p <0.01)].

**Fig 10:**
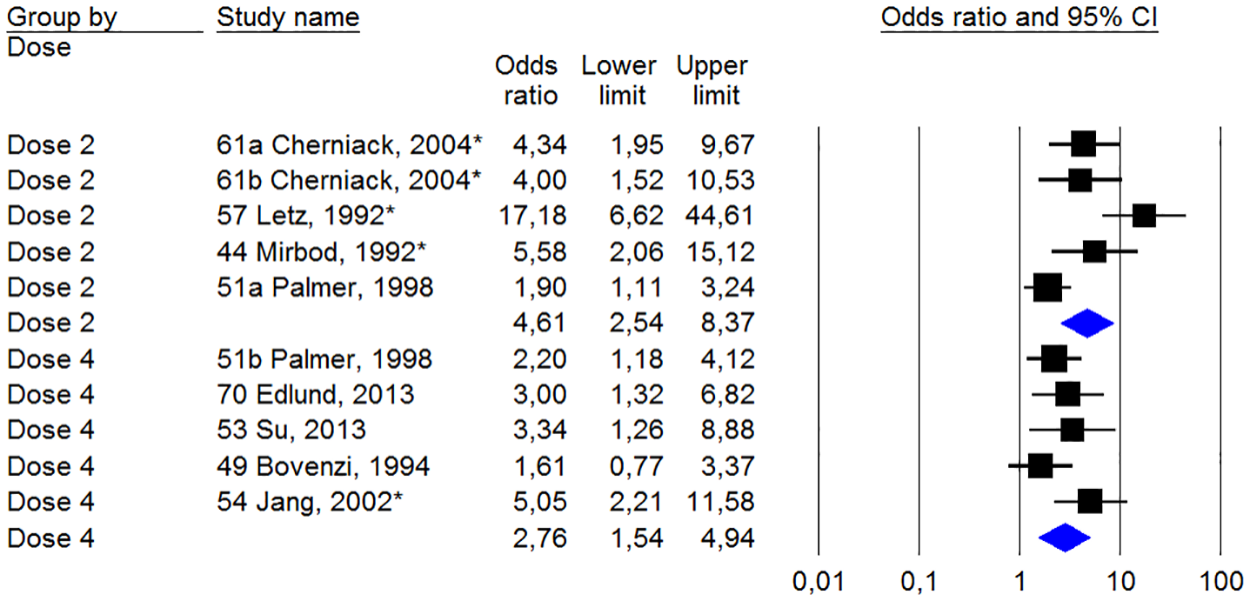
Statistics and Forest plot of the weighting for each dose studies in neuro-sensory injury sorted by the various dose measures. The size of the square of the individual studies is proportional to the study’s importance in the analysis. The blue diamonds (rhomboids) shows the combined effect of the subgroups of dose measures. Dose 2 = Number of exposure hours (h); Dose 4 = Cumulative vibration exposure (mh/s^2^, m^2^h/s^4^ or m^2^h^3^/s^4^). The studies have been ranked in order from highest to lowest quality score points according to Table 2. The asterisk indicates that the study data presented made it possible to calculate the unadjusted odds ratio.

Fig 10 describes the results of the meta-analysis of studies, comparing the risk of neurosensory injury between groups exposed to different levels of HAV. The analysis has also been split between the two categories of measuring doses presented. For each measuring dose, studies have been ranked in descending order according to their total score. In some of the articles several exposure parameters have been entered.

For the two dose measures, the odds ratio varies between 2.67 (p <0:01) to 4.77 (p <0:01). Heterogeneity Dos 2 is 76% (p <0.01) and Dos 4, 15% (p = 0.32).

**Fig 11:**
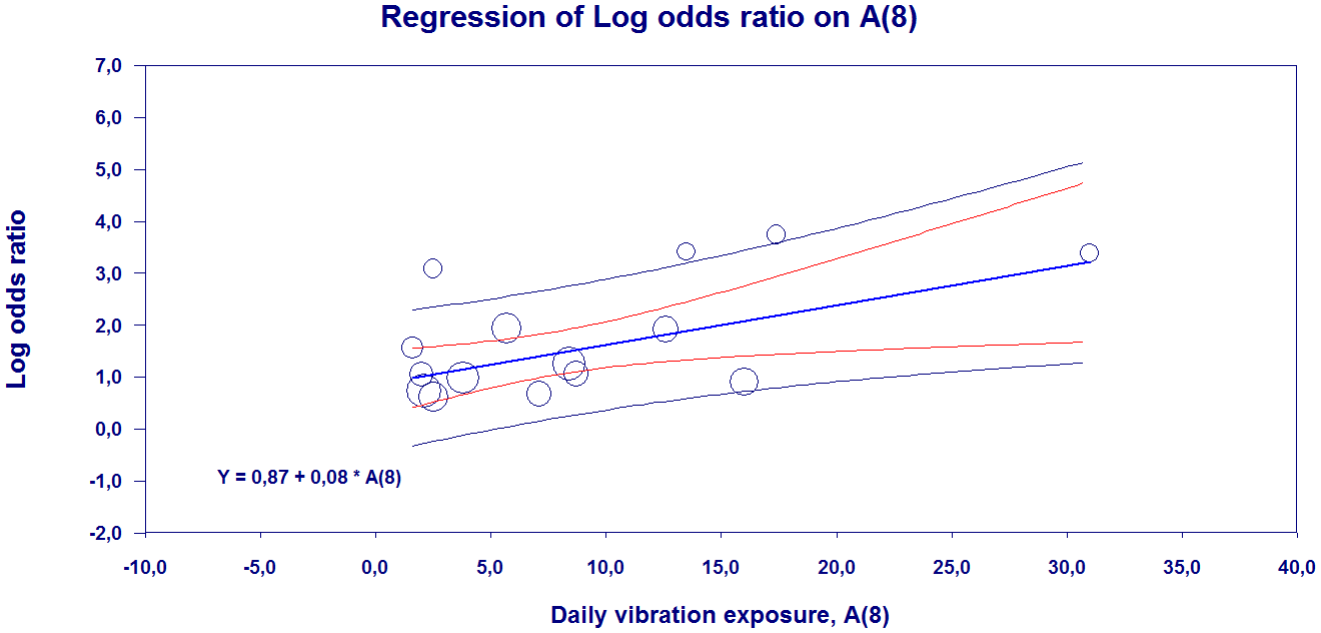
Meta-Regression analysis of the relationship between the logarithm of the prevalence of neurosensory injury in vibration-exposed and vibration exposure A(8) (random effect; n = 15). The figure also shows the regression line 95% confidence limits (red lines). Blue lines show the prediction interval. The size of the circles represents the importance of the study results in the estimates of the regression.

Fig 11 presents the results of meta-regression analysis of the relationship between A(8) and the natural logarithm of the prevalence (logit event rate) of neurosensory injury among vibration-exposed individuals. The result is based on data for 19 articles, and in some of the articles several groups of exposed individuals have been studied.

The analysis shows that the relationship is statistically significant (p = 0.039) and the coefficient of A(8) is 0.08. The results also show that there is a significant (p <0.001) variation between the studies, indicating that, although the A(8) is the same in the two studies, prevalence of neurosensory damage varies. The model explains 5% of the variation between studies.

**Fig 12:**
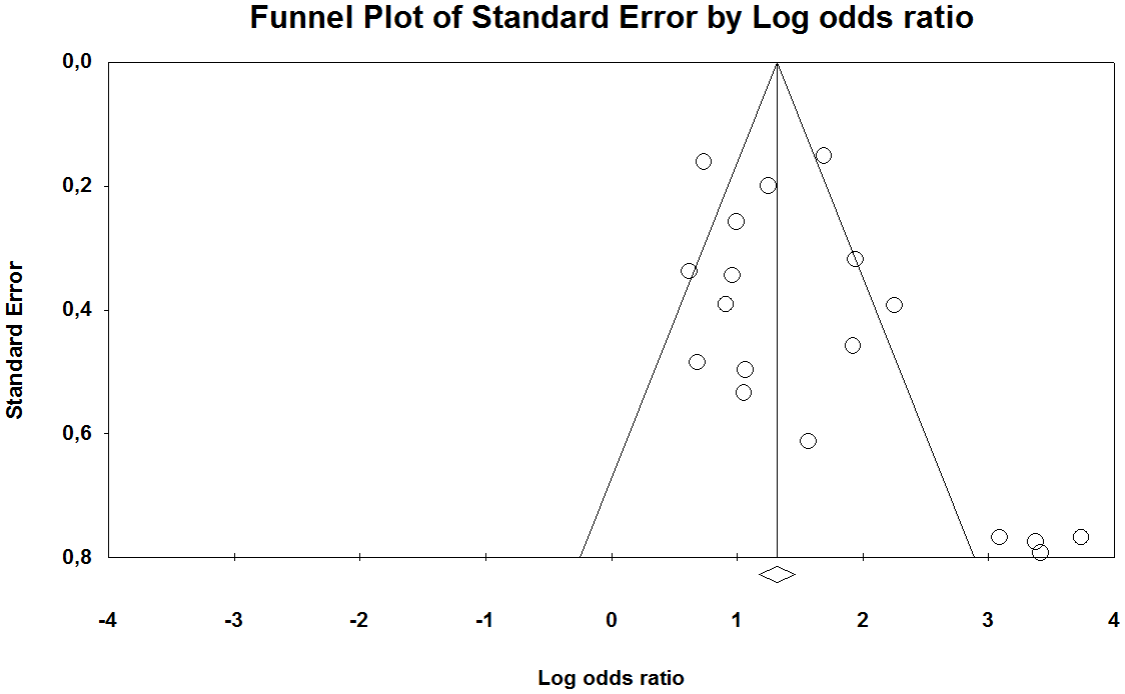
Funnel plot with pseudo 95% confidence interval for publication bias in studies of the association between the occurrences of neuro-sensory injury among groups exposed to HAV and non-exposed reference groups. A Beggs test shows evidence of publication bias (p = 0.04), while Eggers test indicates no evidence the effect (p = 0.07). The trim and fill method imputed no missing study (random-effects model).

**Fig 13:**
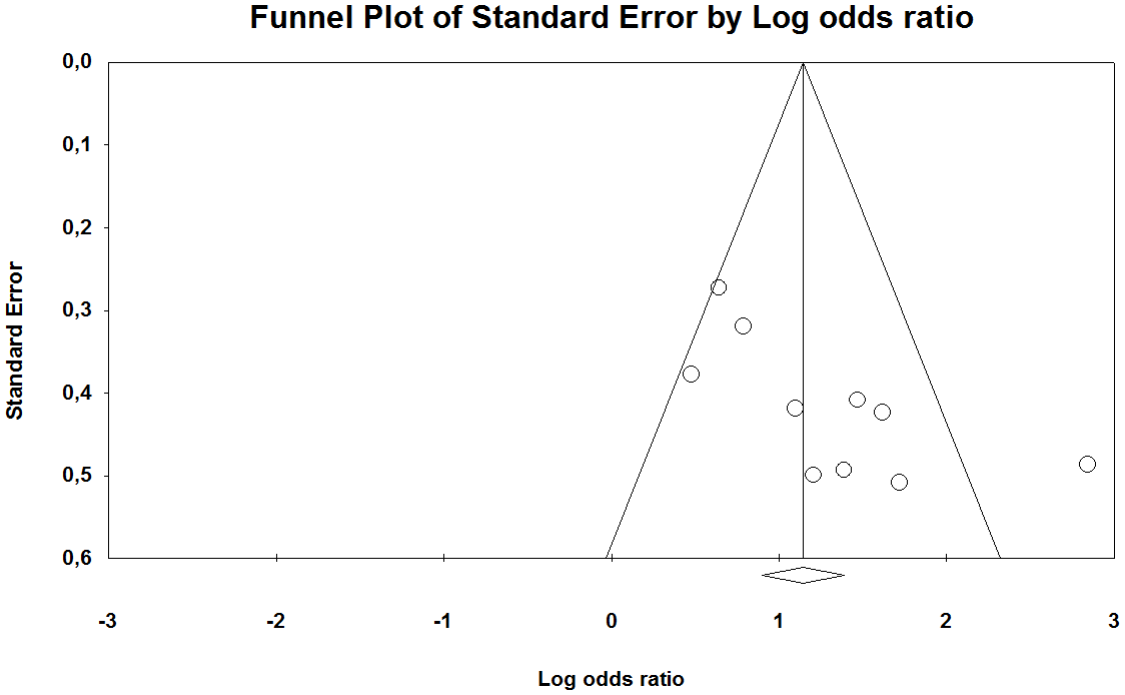
Funnel l plot with pseudo 95% confidence interval for publication bias in studies of the association between the occurrences of neuro-sensory impairment among groups exposed to different levels of HAV. A Beggs test (p = 0.04) and Eggers test (p = 0.02) showed evidence of publication bias while the trim and fill method imputed no missing study (random-effects model).

The funnel plot of the studies included in our meta-analysis indicate that they were distributed asymmetrically around the estimated effect, suggesting some publication bias for both the contrast between HAV exposed and unexposed individuals as well as between low and high exposed groups (Figs 12 and 13).

### Carpal tunnel syndrome

**Fig 14:**
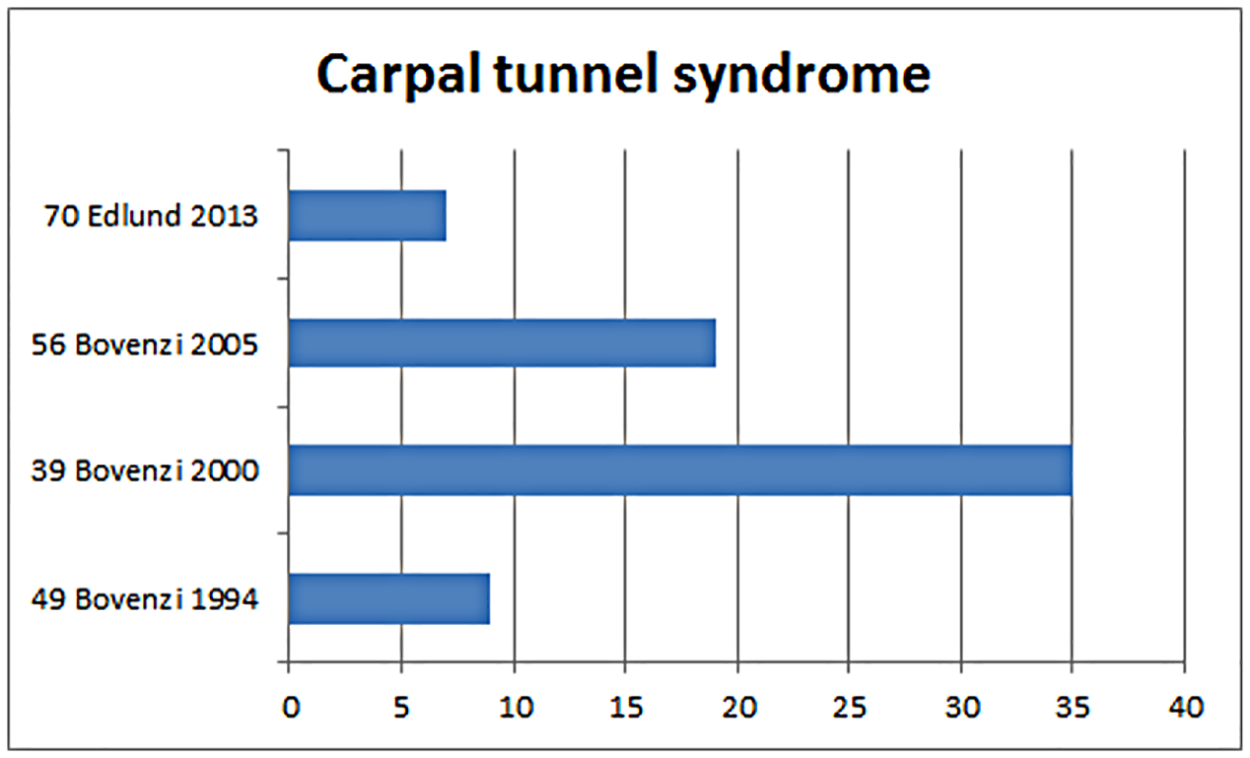
The prevalence of CTS in the studies included sorted by year of publication.

The result of the systematic literature review of articles addressing CTS is presented in Table 3. The table shows both our estimate of risk of bias (S1 Table) regarding the reliability of the diagnosis of “carpal tunnel syndrome” and the total number of quality points. The total is the sum of the quality scores for the diagnosis added to the quality score for “study method” and “exposure”. The summary shows that the seven studies that examined the CTS have a variation of the mean score between 1 and 11 for the diagnosis of CTS. The prevalence of CTS has varied from 7% to 35% in the different studies with an average of 18% (Fig 14).

**Table 3:** Studies included of CTS and their estimated risk of bias (quality score) regarding the diagnosis “Carpal tunnel syndrome” (Diagnosis sum) and the total sum when the quality score for assessing diagnosis’ CTS, “study method” and “exposure” has been added (Total score). The studies are presented in descending order based on the total score. Higher scores indicate higher “quality” indicating less possible risk of bias. Furthermore, the study design is given for each study.

Study	Reference	Design	Diagnosis sum	Total sum
Bovenzi, 2000*	[39]	Case-control	11	26
Sanden, 2010	[34]	Cross-section	11	22
Nilsson, 1994	[32]	Cross-section	11	24
Bovenzi, 2005*	[56]	Cross-section	10	19
Bovenzi, 1994*	[49]	Cross-section	6	17
Edlund, 2013*	[70]	Cohort	1	17
Gerhardsson, 2005	[24]	Cross-section	6	15

*The study included in the meta-analysis

**Fig 15:**
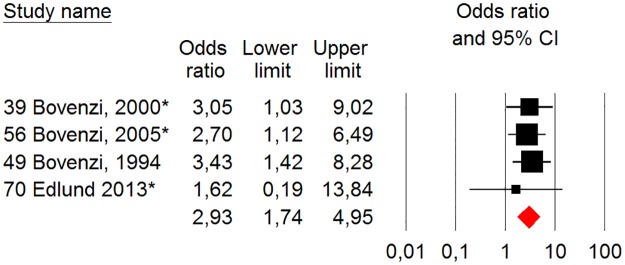
Statistics and “forest plot” with aggregate from “random - effect” meta-analysis of the prevalence of CTS among groups exposed to HAV and non-exposed reference groups. The size of the squares of the individual studies is proportional to the study’s importance in the analysis. The red diamond represents the weighted risk for all studies. The studies have been sorted in order from highest to lowest quality score points according to Table 3. The asterisk indicates that the study data presented made it possible to calculate the unadjusted odds ratio.

Fig 15 presents in a forest plot the results of our meta-analysis of studies comparing the risk of CTS between groups exposed to HAV versus an unexposed reference group. The studies are ranked in descending order according to their overall quality score.

**Fig 16:**
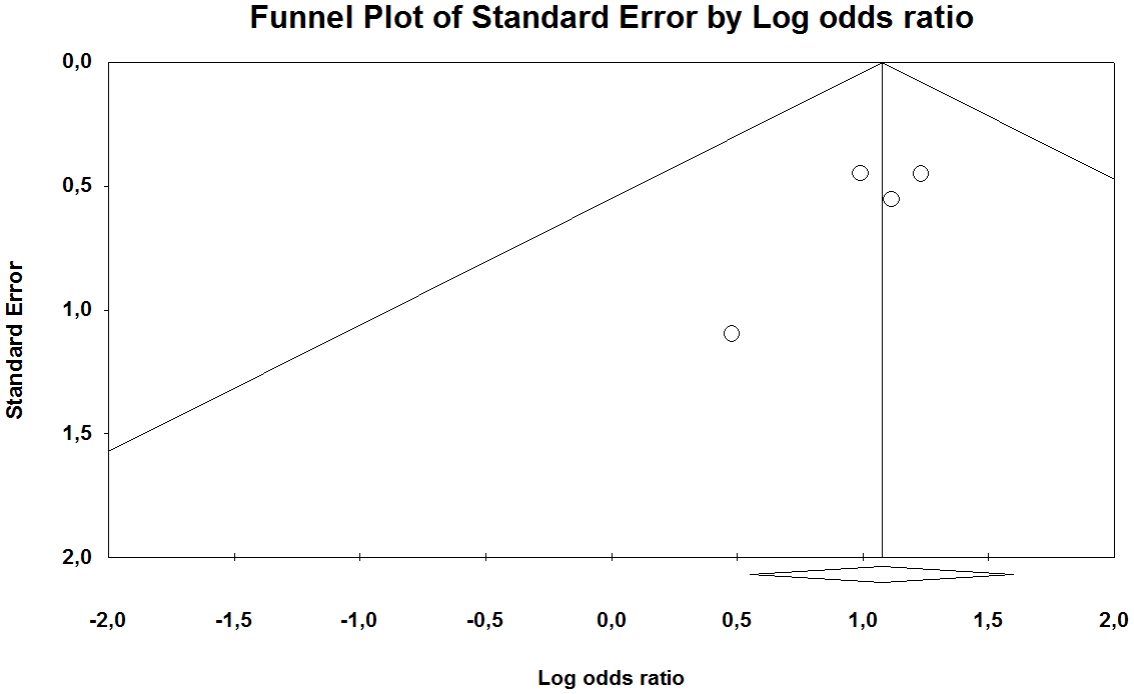
Funnel plot with pseudo 95% confidence interval for publication bias in studies of the association between the occurrences of CTS among groups exposed to HAV and non-exposed reference groups. A Beggs and Eggers test showed no evidence of publication bias (p = 0.50; p = 0.21) and the trim and fill method imputed one missing study to the right of the mean (random-effects model).

For the CTS showed the overall risk estimate an odds ratio of 2.93 (95% CI 1.74–4.95) and 0% (p = 0.93) heterogeneity. The funnel plot of the few studies included in our meta-analysis indicates that they were distributed quite symmetrically around the estimated effect suggesting little publication bias (Fig 16).

**Fig 17:**
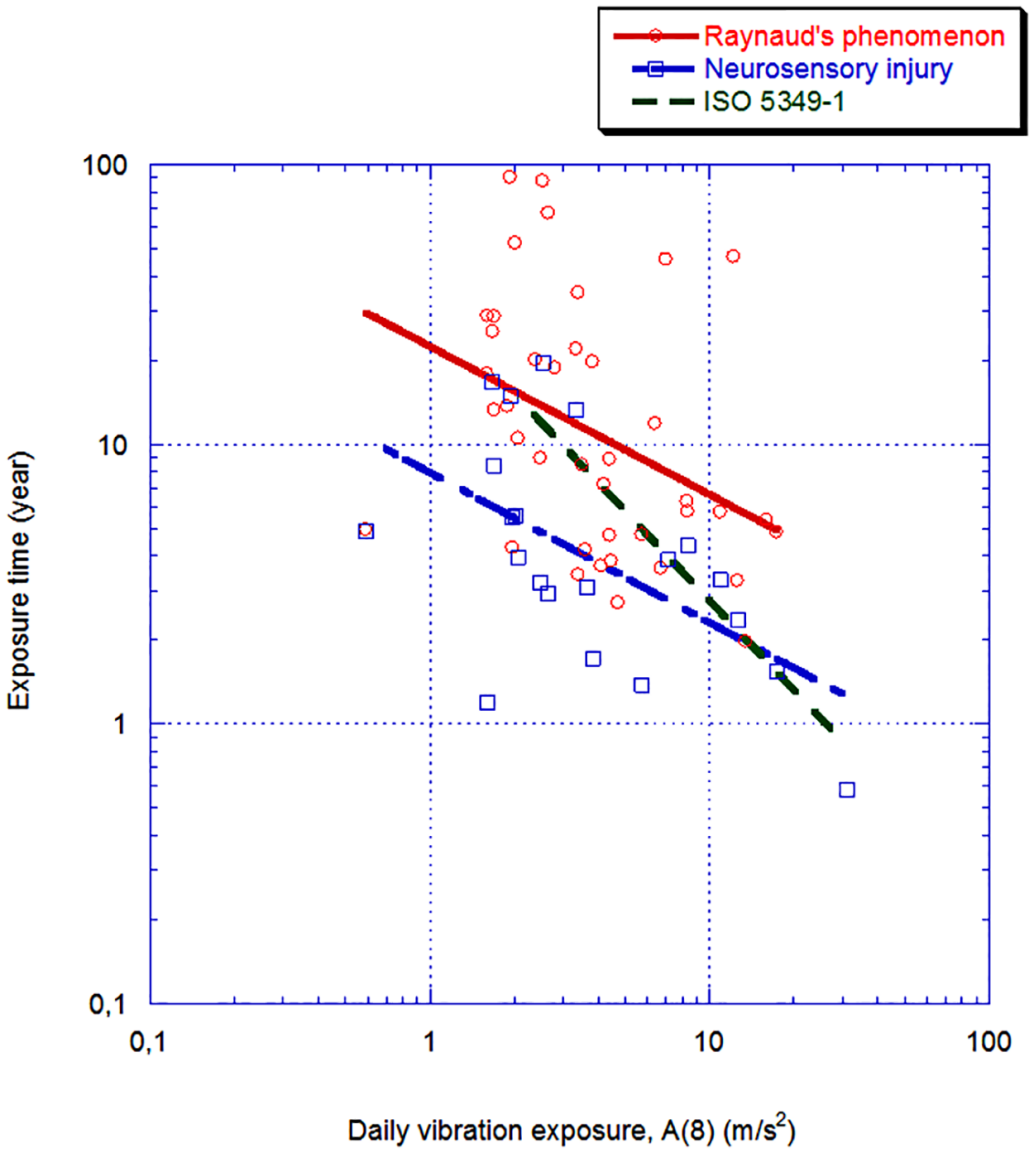
Calculated 10% correlation between the prevalence of Raynaud phenomenon (25 studies; 40 values) and neurosensory injury (17 studies; 21 values) as a function of the 8-hour equivalent frequency-weighted acceleration and number of years of exposure. In the figure shown, the linear regression line for the two outcomes, and the corresponding curve of ISO 5349–1 [(Equations: Prevalence Raynaud phenomenon (%) = 10 ^ (1.35+ log10 (A (8) + - 0.53)) r = 12.39; Prevalence neurosensory damage (%) = 10 ^ (0.9+ log10 (A (8) + - 0.54)), r = 12.55)].

If we use the findings in our review, the following models between exposure and Raynaud’s phenomenon or neurosensory injury can be calculated. The relationship is based on data on the different study groups’ daily vibration exposure, expressed as A(8), the predicted prevalence of symptoms and the group’s medium exposure time (years). We have assumed a linear relationship between occurrence of symptoms and exposure. For example, in a group, the prevalence of symptoms is 40% and the group’s medium exposure time is 20 years. The time before 10% of the group exhibit symptoms can then be calculated to 5 years. The information of the A(8) and the number of years to 10% prevalence can then be used to form a relationship. Such a relation is depicted in Fig 17 for Raynaud’s phenomenon and neurosensory injury. Furthermore, in the figure, the corresponding curve of ISO 5349–1 is shown (green dotted line).

The figure (Fig 17) shows that the disparity of the calculated values is extensive. In the assumptions we made, it appears, based on the calculated regression lines, that at a daily exposure level of 10 m/s^2^ results in a ten per cent prevalence of Raynaud’s phenomenon after 6 years and neuro-sensory impairment after 2 years; the difference is thus a factor of 3. Furthermore, it appears that both regression lines run roughly parallel and have a poor compliance with the prediction based on ISO 5349–1.

## Discussion

This systematic literature review and meta-analysis which covers the scientific literature up to January 2016 shows that workers who are exposed to HAV have an increased risk of vascular and neurological diseases compared to non-vibration exposed groups. The crude estimate of the risk increase is approximately 4–5 fold. The estimated effect size (odds ratio) is 6.9 for the studies of Raynaud’s phenomenon when including only the studies judged to have a low risk of bias. The corresponding risk of neurosensory injury is 7.4 and the equivalent of carpal tunnel syndrome is 2.9. However, for the relation between vibration exposure and carpal tunnel syndrome, the number of studies included is low, which makes this risk estimate less precise and the relation more sensitive to bias.

The comparison of the same groups of vibration-exposed individuals by contrasting high exposure with low exposure showed a pooled risk estimate that varies between 2.5 and 5 for both Raynaud’s phenomenon and neuro-sensory injury and a relation that indicates an exposure-response relationship. Meta-regression analyses also indicate that the risk increases for Raynaud’s phenomenon by a log Odds rate of 0.09% for each exposure increase of 1 m/s^2^. The corresponding increase in risk for neuro-sensory impairment was 0.08%. However, this does not answer the question of how severe the extent of the damage becomes, in each individual case (dose-effect), when the exposure increases. For carpal tunnel syndrome valid exposure-response calculations have not been possible to conduct due to lack of studies.

### Possible bias

#### Possible outcome bias

The definitions of “injury” demonstrate significant shifts in diagnostic and medical content from the early studies to the recent ones. Initially, a collective, all-encompassing term was used for the syndrome “HAVS”, while later the diagnosis became categorized into specific organ entities (vascular, nerve), and thereafter even further sub-grouped. Nerve injury is one example of a collective term which has further been sub-categorized into various sensory units pathology, large- and small fibre neuropathy and entrapment syndrome (CTS). Early studies on neurosensory impairment could, thus, have included both diffuse neuropathy and damage to the nerves in the carpal tunnel, and would in recent studies therefore possibly have been referred to as carpal tunnel syndrome. In our selection of articles, we found studies of carpal tunnel syndrome mainly from the mid-1990s and onwards introducing a diagnostic bias to the early studies.

During the time interval covered, laboratory tests have also been improved and new laboratory tests introduced. Electro-diagnostic tests and various cold provocation tests are such examples. The panorama of injury has also changed during this period. The early studies mainly reported vascular injury, while recent studies report a relatively higher occurrence of nerve injury, an observation that might reflect a real transition in the injury panorama but could also be an effect of attention bias or publication bias.

The level of diagnostic precision also varies between different studies and over time. Numerous studies define the outcome as symptom descriptions only, while others use semi objective or objective tests at diagnosis. A number of studies differentiate minor injury against serious harm by classification, but the majority of the studies indicate only the presence of symptoms, which means that more advanced injury may be equated with minor damage. The presently advocated “Stockholm workshop scales” [73, 74] to classify vascular and neurological disorders lack precision for several of the terms used and are thus unable to clearly differentiate minor injury from severe disease

The results of our meta-analysis reveal that effect sizes vary between the studies with a low risk of bias (“high quality” studies) compared with the studies with increased (“low quality studies) risk of bias. For the studies with a low risk of bias, we found an overall risk of Raynaud’s phenomenon of 6.8, while the equivalent of studies with a lower quality was 3.6. The relationship is similar for neuro-sensory impairment with a risk of 7.8 and 3.3, respectively.

#### Possible Exposure bias

Measuring instruments, electronics and measurement methodologies have developed and have thereby varied over time, but since 1976 have been standardized under ISO (ISO 5349). The most obvious difference in method involves measuring the dominant direction to sum vector, expressed as an initial 4-hour equivalent and subsequently the 8-hour equivalent value and with or without frequency weighting. In this report, if at hand, we have consistently used or recalculated all exposures to the 8-hour equivalent frequency-weighted vibration, which allows comparability between different studies. Levels of exposure in various studies have been calculated as the group mean and not as individual cumulative dose. This means that anyone who showed signs or expressed symptoms may have received these in a previous exposure alternatively previously been anywhere from heavy to insignificantly exposed. Moreover, in the studies included, the exposures occurred at many different levels and were quantified in different ways. This means that those who in one study were considered low-exposed might in another study be regarded as highly exposed.

The results of this review could be biased by confounding exposures. Hand intensive work, cold, local stress and a power grip can all constitute contributory factors for the occurrence of vascular and nerve damage. Besides being a confounder for the occurrence of both white finger and nerve damage, cooling is also a trigger for attacks of Raynaud’s phenomena and cold intolerance. Most studies have been conducted in the temperate climate zone, and studies have shown that Raynaud’s phenomenon does not manifest in hot climates [52].

To investigate whether other factors may explain the occurrence of injury, we have gone over all the studies and examined whether they have taken into account a possible Primary Raynaud`s phenomenon and effect-modifying factors or confounders. We have looked for information in the studies of whether the authors have taken into account other diseases and whether standard laboratory screening tests for inflammatory disease and polyneuropathy has been performed. The result is expressed in terms of high or low risk for diagnosis bias (quality score).

#### Possible method bias

During our primary systematic literature search of publications, we followed the conventional electronic database-based literature search on several search engines. Through our broad approach, our quality testing and renewed searches, complemented by a manual review of non-indexed reports, we estimate that the final literature search was sensitive enough (number needed to read = 83 for the electronic search and NNR = 100 for the hand search) to give a valid picture of the scientific base as it is reflected in Western scientific publications. Our restriction to English-language publications may cause the relevant Russian, French, German, Spanish and Chinese science to be overlooked.

The literature search process has been marred by duplication bias and citations bias. A few writers have repeatedly published reports based on the same study population. In the duplications, only the publication that we by consensus have deemed to be most relevant has been included in the Meta-analyses. In order to provide the reader with transparent information about the publication bias, funnel plots for standard error in relation to effect size as log odds ratios have been presented. This is with the proviso that the standard error is related to the study population log-odds (115). Signs of publishing bias are found for both white finger and nerve injury. This may indicate that the risk estimate is possibly overstated. This is most clearly seen in a number of publications with few participants but with high risks.

The statistical synthesis is based on a meta-analysis according to the “Comprehensive Meta Analyses” statistical package. In cases where there are empty cells, a conservative approach with the addition of “1” in all cells been utilized. Meta-analyses in which this adjustment has been used were, for example, where there was no occurrence of “white fingers” [52]. Calculation where all studies with 0 cells were excluded gave a change in the overall crude risk of 3.6 to 3.1 and for the subgroup of high-quality studies from 6.8 to 6.5.

The scientific literature on vibration health hazards is dominated by cross-sectional studies. In the descriptive assessment of individual studies’ effect size, we found prevalence rates of the magnitude that challenge the use of odds ratio. The overestimation of the risk that might result has been considered in our overall judgement. We have in our grading of risk for bias weighted the type of study and in addition to the meta-analysis taken into account the design in determining the effect size.

### Aspects on risk assessment

The risk assessment in the annex of ISO 5349–1 is based on the relation to the level of vibration intensity, number of years of exposure and the prevalence of Raynaud’s phenomenon. Also, this annex is often used for neurological injuries. Our results show that the relationships between vibration exposure and Raynaud’s phenomenon and neurosensory injury represent different functions. Risk assessment of neuro-sensory impairment based on the data of ISO 5349 may thus be misleading. The European vibration directive [75] could, therefore, suffer from the same weakness. The need for revision of the risk assessment model is thus important. A conclusion compatible with that from the UK´s Health and Safety Laboratory “A critical review of evidence related to hand-arm vibration syndrome and the extent of exposure to vibration”, which claims that there are still a number of unknowns with regard to the exposure—response relationships for HAVS [76].

Our literature review also reveals that there is no research on the interactions between various diseases and the various manifestations of HAVS. The effect of such comorbidity with vibration injury has in recent decades received increased attention and has gained in clinical importance, since the occurrence of chronic diseases increases in a work population who work at higher age. Interactions can occur not only between HAVS and various diseases, but also with the medication given for medical risk factors (such as the treatment of blood pressure). Knowledge of how different diseases interact (comorbidity) with the onset and worsening of HAVS as well as the influence of basal body functions such as sleep and fitness, are currently lacking in the literature.

An issue closely related to comorbidity is the question on how individuals’ potential vulnerability to vibration exposure modifies the development of HAVS. Comorbidity, age-related modifying factors and the possible interactions with drugs and other vasoactive or nerve-disturbing exposures have mostly been overlooked in present research. Risk information about the dangers of working with vibrating machines related to age and susceptibility lacks evidence-based knowledge. Vulnerability factors for the occurrence of various manifestations of HAVS need to be identified.

## Conclusions

Workers who are exposed to HAV have an increased risk of vascular and neurological diseases compared to non-vibration-exposed groups. At equal exposures, neurosensory injury occurs with a 3-time factor shorter latency than Raynaud’s phenomenon, which is why more preventive measures should more aggressively address this vibration health hazard.

## Supporting information

S1 Table Evaluation of risk of bias. (DOCX)

S2 Table Dataset from S2. (XLSX)

S3 Table PRISMA 2009 checklist. (DOC)

S1 Text Search strategy. (DOCX)

S2 Text Alternative language report. Previous, extended report in Swedish.
(PDF)
