# Supplementary material for: Hand-arm vibration and the risk of vascular and neurological diseases—A systematic review and meta-analysis
Source: PLoS One. 2017 Jul 13;12(7):e0180795. doi: 10.1371/journal.pone.0180795 (PMC5509149; doi:10.1371/journal.pone.0180795)
Supplement: S1 Table — (DOCX) [file pone.0180795.s001.docx]

| S2 Table. Evaluation of risk of bias (quality criteria)  **Raynaud’s phenomenon** | | |
| --- | --- | --- |
| Criterion | Alternative | Score |
| Subjective symptom description | Doctor’s anamnesis (interview) Symptom report and colour chart* Questionnaire Missing | 3 2 1  0 |
| Clinical investigation | Doctor’s examination Missing | 2  0 |
| Objective investigation | COP, FSBP  Re-warming  Missing | 2 1  0 |
| Control of diagnostic methods | Method-, Subject, environment  Missing | 1  0 |
| Checking for diff. diagnosis alternative causes | Doctor’s examination, lab screening, medicine  Missing | 1  0 |
| Stadium classified | Yes No | 2  0 |
| **Neurosensory symptoms** | | |
| Criterion | Alternative | Score |
| Subjective symptom description | Doctor’s anamnesis (interview) Anamnesis  Questionnaire Missing | 3 2 1 0 |
| Clinical investigation | Doctor’s examination Missing | 2  0 |
| Objective investigation | Morphological or Electrical Diagnostics Missing | 2 0 |
| Semi-objective investigation | QST: vibration, temperature, other Missing | 2 0 |
| Control of diagnostic methods | Method-, Subject, environment  Missing | 1 0 |
| Checking for diff. diagnosis alternatively cause | Doctor’s examination, lab screening, medicine  Missing | 1 0 |
| Stadium classified | Yes No | 2  0 |
| **Carpal tunnel syndrome** | | |
| Criterion | Alternative | Score |
| Subjective symptom description | Doctor’s anamnesis (interview) Symptom report  Questionnaire Missing | 3 2 1  0 |
| Clinical investigation | Doctor’s examination, Phalen, Tinel  Missing | 2  0 |
| Objective investigation | Morphological or Electrical Diagnostics Missing | 4  0 |
| Control of diagnostic methods | Method, Subject, environment  Missing | 1  0 |
| Checking for diff. diagnosis alternatively cause | Doctor’s examination, lab screening, medicine  Missing | 1  0 |
| Stadium classified | Yes No | 2  0 |
| **Exposure** | | |
| Criterion | Alternative | Score |
| Current exposure acceleration (m/s^2^) | Objective measures  Subjective estimates  No information | 2 1  0 |
| Current exposure time (hours/day) | Objective measures  Subjective estimates  No information | 2 1 0 |
| Information about previous acceleration | Objective measures  Subjective estimates  No information | 2 1  0 |
| Information about previous exposure (years) | Objective measures  Subjective estimates  No information | 2 1  0 |
| Information about previous exposure (hours/day) | Objective measures  Subjective estimates  No information | 2 1  0 |
| **Method** | | |
| Criterion | Alternative | Score |
| Study design | RCT  Cohort  Case-control  Cross-section | 8  6  4 2 |
| Selection | Response rate higher than 70% or falling off at follow-up less than 30% Not met | 2   0 |
| Control of individual confounding factors | Yes No | 2 0 |

* Negro C, Rui F, D'Agostin F, Bovenzi M. Use of color charts for the diagnosis of finger whiteness in vibration-exposed workers. International archives of occupational and environmental health. 2008 Apr;81(5):633-8.
