# Supplementary material for: Hand-arm vibration and the risk of vascular and neurological diseases—A systematic review and meta-analysis
Source: PLoS One. 2017 Jul 13;12(7):e0180795. doi: 10.1371/journal.pone.0180795 (PMC5509149; doi:10.1371/journal.pone.0180795)
Supplement: S1 Text — (DOCX) [file pone.0180795.s004.docx]

S1 Text. Search strategy

The search string is based on the keywords “Exposure and disorder/symptoms.” These keywords have since been specified and the search string constructed had the following appearance:

Search (((((((((“vibration”[MeSH Terms] OR vibration[Text Word])) OR Vibrations) OR Vibration adj2 expo*) OR Segmental adj2 vibration*)) AND ((((((((((((((“hand-arm vibration syndrome”[MeSH Terms] OR hand arm vibration syndrome[Text Word])) OR (“hand-arm vibration syndrome”[MeSH Terms] OR hand arm vibration syndromes[Text Word])) OR “hand-arm vibration syndrome”[MeSH Terms]) OR (“peripheral vascular diseases”[MeSH Terms] OR peripheral vascular disease[Text Word])) OR peripheral vascular diseases) OR arterial disease peripheral) OR (“Raynaud disease”[MeSH Terms] OR Raynaud disease[Text Word])) OR (“Raynaud disease”[MeSH Terms] OR Raynaud phenomenon[Text Word])) OR (“Raynaud disease”[MeSH Terms] OR Raynaud s disease[Text Word])) OR vibration syndrome) OR white adj2 finge*) OR vibration adj2 white) OR Raynaud*))) OR (((((((“vibration”[MeSH Terms] OR vibration[Text Word])) OR Vibrations) OR Vibration adj2 expo*) OR Segmental adj2 vibration*)) AND ((((nerve) OR (“peripheral nervous system diseases”[MeSH Terms] OR peripheral neuropathies[Text Word])) OR (“nerve compression syndromes”[MeSH Terms] OR nerve compression syndromes[Text Word])) OR (“carpal tunnel syndrome”[MeSH Terms] OR carpal tunnel syndrome[Text Word]))))
