# Supplementary material for: Hand-arm vibration and the risk of vascular and neurological diseases—A systematic review and meta-analysis
Source: PLoS One. 2017 Jul 13;12(7):e0180795. doi: 10.1371/journal.pone.0180795 (PMC5509149; doi:10.1371/journal.pone.0180795)
Supplement: S2 Text — Previous, extended report in Swedish. (PDF) [file pone.0180795.s005.pdf]

ARBETE OCH HÄLSA Nr 2016;49(4)
VETENSKAPLIG SKRIFTSERIE

# Systematiska kunskapsöversikter; 9. Kärl- och nervskador i relation till exponering för handöverförda vibrationer

*Tohr Nilsson*
*Jens Wahlström*
*Lage Burström*

ISBN 978-91-85971-56-5
ISSN 0346–7821

ARBETE & HÄLSA gavs ut av Arbetsmedicinska institutet från 1967 och kallades då AI-rapporterna. När institutet införlivades med Arbetaskyddsverket år 1972 fick skriftserien sitt nuvarande namn. Därefter tog Arbetsmiljöinstitutet över skriftserien år 1987, sedan Arbetslivsinstitutet 1995 och till sist Arbets- och miljömedicin vid Göteborgs universitet från 2007.
Skriftserien har sedan start varit ett viktigt instrument för de statliga verksamheterna att dokumentera och sprida forskningsresultat. I serien publiceras vetenskapliga originalarbeten, översiktsartiklar, kriteriedokument, och doktorsavhandlingar. Samtliga publikationer är refereegranskade. Arbete och Hälsa har en bred målgrupp med artiklar inom skilda områden som är relaterade till arbetsmiljö och hälsa i arbetslivet.

ANSVARIG UTGIVARE
Kjell Torén, Göteborgs universitet

REDAKTION
Maria Albin, Stockholm
Lotta Dellve, Stockholm
Henrik Kolstad, Århus
Roger Persson, Lund
Kristin Svendsen, Trondheim
Allan Toomingas, Stockholm
Marianne Törner, Göteborg

REDAKTIONSASSISTENT
OCH GRAFISK FORM
Cecilia Andreasson,
Göteborgs universitet

REDAKTIONSRÅD
Gunnar Ahlborg, Göteborg
Kristina Alexanderson, Stockholm
Berit Bakke, Oslo
Lars Barregård, Göteborg
Jens Peter Bonde, Köpenhamn
Jörgen Eklund, Linköping
Mats Hagberg, Göteborg
Kari Heldal, Oslo
Kristina Jakobsson, Göteborg
Malin Josephson, Uppsala
Bengt Järvholm, Umeå
Anette Kærgaard, Herning
Ann Kryger, Köpenhamn
Carola Lidén, Stockholm
Svend Erik Mathiassen, Gävle
Gunnar D. Nielsen, Köpenhamn
Catarina Nordander, Lund
Torben Sigsgaard, Århus
Gerd Sällsten, Göteborg
Ewa Wikström, Göteborg
Eva Vingård, Stockholm

KONTAKT / PRENUMERERA / BESTÄLL NUMMER / SKICKA MANUS
E-post: arbeteochhalsa@amm.gu.se
Telefon: 031-786 62 61
Postadress: Arbete och hälsa, Box 414, 405 30 Göteborg
Hemsidor: www.amm.se/aoh
gupea.ub.gu.se/handle/2077/3194
En prenumeration kostar 800 kr per år exklusive moms (6%).

© Göteborgs universitet & Författarna
ISSN 0346–7821
Tryckeri: Kompendiet, Göteborg

# Innehållsförteckning

Förord 1

Inledning 3
Akuta effekter 5
Bestående effekter 5

Motiv och övergripande syfte 6
Sambandet hand-arm vibration och Raynauds fenomen 6
Sambandet hand-arm vibration och neurosensorisk skada 7
Sambandet hand-arm vibration och karpaltunnelsyndrom 7

Metod 8
Systematisk litteratursökning 8
Resultat av den övergripande systematiska litteratursökningen 9
Branscher och uppmätta exponeringsnivåer 13
Metaanalys 14

Raynauds fenomen (”Vita fingrar”) 15
Definition 15
Diagnostisk tillförlitlighet och risk för bias 15
Gradering av ”kärlskadans” allvarlighetsgrad 16
Möjlig bias till följd av alternativa orsaker till Raynauds fenomen 19
Möjlig bias till följd av interaktion från andra exponeringsfaktorer 19
Resultat av den systematiska litteratursökningen för Raynauds fenomen 19
Resultat av meta-analys från studier på ”Raynauds fenomen” 23
Summering 26

Neurosensorisk skada 28
Definition 28
Diagnostiska överväganden och risk för bias 29
Gradering av skadans allvarlighetsgrad 29
Möjlig bias till följd av interaktion från andra exponerings- och individfaktorer 30
Resultat av den systematiska litteratursökningen för neurosensorisk skada 31
Resultat av meta-analys från studier på neurosensorisk skada 34
Summering 39

Karpaltunnelsyndrom 39
Definition 39
Diagnostiska överväganden och risk för bias 39
Gradering av ”karpaltunnelsyndromets” allvarlighetsgrad 40
Möjlig bias till följd av alternativa orsaker till karpaltunnelsyndrom. 40
Möjlig bias till följd av interaktion från andra exponeringsfaktorer 40
Resultat av den systematiska litteratursökningen för karpaltunnelsyndrom 40
Resultat från meta-analys på studier av karpaltunnelsyndrom 42
Summering 42

Diskussion 43
Diagnostisk tillförlitlighet 44
Analytisk tillförlitlighet 45
Riskbedömning i relation till ISO-5349-annex och Europeiska vibrationsdirektivet 48

Angelägen information som saknats vid kunskapsgenomgången 51
Uppmärksammade kunskapsluckor 51
Uppmärksammade brister i teoribildning 52
Uppmärksammade metodsvagheter 53
Uppmärksammade brister i informationsspridning och prevention 53

Referenser 55

Redaktörernas slutord 63

Appendix 65
Bilaga 1. Använd söksträng i litteraturdatabasen 67
Bilaga 2. Använda kvalitetskriterier 68
Bilaga 3. Störningar och faktorer förknippade med sekundärt Raynauds fenomen 70
Bilaga 4. Störningar och faktorer förknippade med nervskada (neuropati) 72
Bilaga 5. Störningar och faktorer förknippade med karpaltunnelsyndrom 74

# Förord

Denna utgåva ingår i den serie av systematiska kunskapssammanställningar som ges ut av Göteborgs Universitet. Dessa kunskapssammanställningar hade sin bakgrund i ett behov att ange riktlinjer hur man fastställer samband i arbetsskadeförsäkringen. Arbetet inleddes 1981 när en grupp ortopeder, yrkesmedicinare, andra arbetsmiljöforskare och läkare från LO diskuterade i Läkartidningen en modell för bedömning av vilka arbetsställningar som utgjorde skadlig inverkan för besvär i bröst och ländrygg. Gruppen pekade också på vikten av att systematiskt ställa samman kunskap inom området (Andersson 1981). Därefter publicerades flera systematiska kunskapssammanställningar med avsikt ge riktlinjer för förekomst av skadlig inverkan vid arbetsskadebedömningar (Westerholm 1995, 2002, Hansson & Westerholm 2001).

Göteborgs Universitet är nu huvudansvarigt för ett flerårigt projekt med avsikt att ta fram nya kunskapssammanställningar inom arbetsmiljöområdet. Arbetet har finansiellt stöd av AFA försäkring, Forte och Göteborgs Universitet. Dessa systematiska kunskapssammanställningar har som syfte att beskriva arbetsmiljöns betydelse för uppkomst eller försämring av sjukdom eller symptom i ett bredare perspektiv. Tillämpningen av resultaten får ske inom berörda myndigheter, arbetsplatser och försäkringsbolag.

Dessa systematiska kunskapssammanställningar genomförs av experter inom respektive området. Deras bedömning granskas sedan av andra experter inom området. Den nya serien av systematiska kunskapssammanställningar inleddes 2008 med en förnyad översikt om psykisk arbetsskada (Westerholm 2008), som sedan följdes av sammanställningar om fukt och mögel, helkroppsvibrationer och en förnyad uppdatering av arbetets betydelse för uppkomst av depression, stroke, Parkinsons sjukdom, ALS och demens (Torén 2010, Burström 2012, Lundberg 2013, Jakobsson 2013, Gunnarsson 2014, 2015a, b). Dessutom har vi tagit fram ett mycket efterfrågat dokument om hur diabetiker klarar av olika påfrestande arbetsmiljöer (Knutsson 2013). Eftersom kunskapsläget förändras finns det ett behov av uppdateringar av gamla kunskapssammanställningar samtidigt som det finns ett behov av kunskapssammanställningar inom nya områden.

Denna kunskapssammanställning belyser frågan om exponering för hand-arm överförda vibrationer orsakar kärlskada och nervskada (inklusive karpaltunnelsyndrom). Forskarnas Tohr Nilsson, Jens Wahlström och Lage Burström, samtliga vid Umeå Universitet, har genomfört denna systematiska kunskapssammanställning. Externa referenter har varit seniorprofessor Lars Barregård, Göteborgs Universitet och docent Istvan Balogh, Lunds Universitet. Vi är tacksamma för de värdefulla och konstruktiva bidrag som referenterna har tillfört detta arbete.

Göteborg, Lund och Umeå, februari 2016
Kjell Torén, Maria Albin, Bengt Järvholm

# Referenser


Andersson G, Bjurvall M, Bolinder E, Frykman G, Jonsson B, Kihlbom Å, Lagerlöf E, Michaëlsson G, Nyström Å, Olbe G, Roslund J, Rydell N, Sundell J, Westerholm P. Modell för bedömning av ryggskada i enlighet med arbetsskadeförsäkringen. Läkartidningen 1981;78:2765-2767.

Burström L, Nilsson T, Wahlström J. Exponering för helkroppsvibrationer och uppkomst av ländryggssjuklighet. I; Torén K, Albin M, Järvholm B (red). Systematiska kunskapsöversikter; 2. Exponering för helkroppsvibrationer och uppkomst av ländryggssjuklighet. Arbete och Hälsa 2012;46(2).

Gunnarsson LG, Bodin L. Systematiska kunskapsöversikter; 6. Epidemiologiskt påvisade samband mellan Parkinsons sjukdom och faktorer i arbetsmiljön. Arbete och Hälsa 2014;48(1).

Gunnarsson LG, Bodin L. Systematiska kunskapsöversikter; 7. Epidemiologiskt påvisade samband mellan ALS och faktorer i arbetsmiljön. Arbete och Hälsa 2015;49(1).

Gunnarsson LG, Bodin L. Systematiska kunskapsöversikter; 8. Epidemiologiskt påvisade samband mellan Alzheimers sjukdom och faktorer i arbetsmiljön. Arbete och Hälsa 2015;49(3).

Hansson T, Westerholm P. Arbete och besvär i rörelseorganen. En vetenskaplig värdering av frågor om samband. Arbete och Hälsa 2001:12.

Jakobsson K, Gustavsson P. Systematiska kunskapsöversikter; 5. Arbetsmiljöexponeringar och stroke – en kritisk granskning av evidens för samband mellan exponeringar i arbetsmiljön och stroke. Arbete och Hälsa 2013;47(4).

Knutsson A, Kempe A. Systematiska kunskapsöversikter; 4. Diabetes och arbete. Arbete och Hälsa 2013;47(3).

Lundberg I, Allebeck P, Forsell Y, Westerholm P. Systematiska kunskapsöversikter; 3. Kan arbetsvillkor orsaka depressionstillstånd. En systematisk översikt över longitudinella studier i den vetenskapliga litteraturen 1998-2012. Arbete och Hälsa 2013;47(1)

Torén K, Albin M, Järvholm B. Systematiska kunskapsöversikter; 1. Betydelsen av fukt och mögel i inomhusmiljön för astma hos vuxna. Arbete och Hälsa 2010;44(8).

Westerholm P. Arbetssjukdom – skadlig inverkan – samband med arbete. Ett vetenskapligt underlag för försäkringsmedicinska bedömningar (6 skadeområden). Arbete och Hälsa 1995;16.

Westerholm P. Arbetssjukdom – skadlig inverkan – samband med arbete. Ett vetenskapligt underlag för försäkringsmedicinska bedömningar (7 skadeområden). Andra, utökade och reviderade upplagan. Arbete och Hälsa 2002;15

Westerholm P. Psykisk arbetsskada. Arbete och Hälsa 2008;42:1

# Kärl- och nervskador i relation till exponering för handöverförda vibrationer

## Inledning

Handöverförda vibrationer, så kallade hand-arm vibrationer (HAV), förekommer inom arbetslivet i samband med arbete med vibrerande maskiner, verktyg eller arbetsobjekt som hålls eller stöds av handen, till exempel slipmaskiner, skruvdragare, bergborrar och mejselhammare.

HAV har kvantifierats och mätts sedan mitten av 1940-talet (1) men mätningar blev mer vanligt förekommande först på 1960-talet. Mot bakgrund av diskussioner om hur mätningarna skulle kunna standardiseras, formulerades under 1970-talet ett första förslag till en internationell standard (ISO 5349) (2) som beskriver hur mätning och analys av HAV skall genomföras. Enligt standarden mäts vibrationer inom ett frekvensområde från 5 till 1500 Hz på den vibrerande yta där den exponerade kommer i kontakt med vibrationerna. Storleken (nivån, magnituden) på vibrationerna beskrivs i storheten acceleration (A). Mätningarna sker i tre mot varandra vinkelräta riktningar x, y, och z. Uppmätt acceleration viktas (vägs) därefter för att ta hänsyn till att människans förmåga att uppfatta vibrationer är olika vid olika frekvenser. Resultatet summeras därefter för de tre riktningarna till ett frekvensvägt accelerationsmått och anges i enheten $m/s^2$.

Figur 1 illustrerar exempel på genomsnittliga exponeringsnivåer som uppmätts vid arbete med några olika handhållna maskiner. Dessa mätningar omfattar både äldre och nyare maskiner. I figuren anges även spridningen av mätvärdena för respektive maskintyp.

Figuren visar att uppmätta vibrationsnivåer varierar kraftigt mellan olika typer av handhållna maskiner samt att spridningen inom samma maskintyp kan vara påtaglig. Denna variation beror bland annat på att exponeringen uppmätts för olika maskinfabrikat med olika effekt och med både äldre och nyare maskiner. Vidare beror spridningen på att olika arbetsmoment undersökts och på att maskinen har hanterats av olika användare.

I ISO standarden från 1979 infördes även gränser för rekommenderad daglig exponeringstid beroende på vibrationernas frekvensinnehåll. Dessa gränser bygger på studier som visade att det krävs högre accelerationsnivå för att ge en upplevelse av obehag vid högre vibrationsfrekvenser. Detta underlag förenklades och generaliserades därefter av arbetsgruppen inom ISO, för att fastslå de rekommenderade gränserna för daglig exponeringstid beroende på vibrationernas frekvensinnehåll (3). Vidare antogs att upplevelsen av obehag vid vibrationsexponering var direkt relaterad till risken för vibrationsrelaterade skador. Dessa frekvenskurvor utgjorde fundamentet för den frekvensvägning som introducerats i den efterföljande standarden från 1986 för utvärdering av riskerna med exponering för HAV (4).
I den standarden infogades också ett diagram över sambandet mellan HAV-

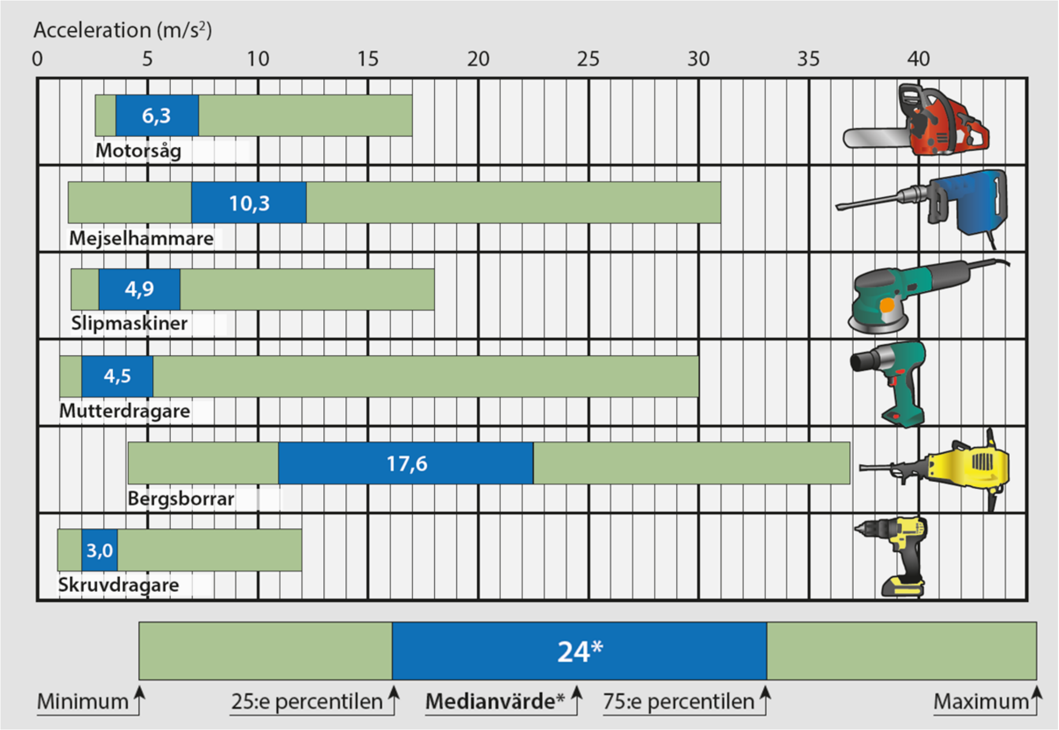


**Figur 1:** Exempel på uppmätta vibrationsexponeringsnivåer och variationsvidd i m/s$^2$ för några olika handhållna maskiner (uppgifterna hämtade från Vibrationsdatabasen, Umeå universitet http://www.vibration.db.umu.se/).

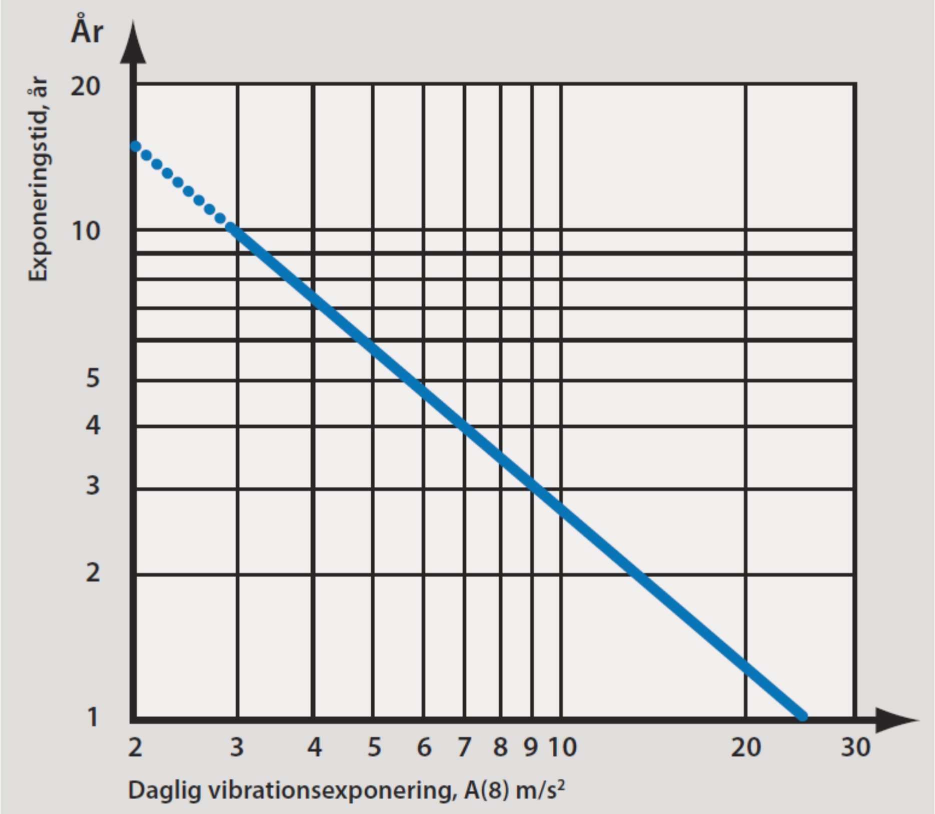


**Figur 2:** Samband mellan 10 procentig förekomst (prevalens) av vibrationsskada i form av "vita fingrar" bland vibrationsexponerade som funktion av 8-timmar ekvivalent frekvensvägd acceleration och antal år med exponering (5).

exponering, uttryckt i år med daglig exponeringsnivå, och risk för uppkomst av blodflödesstörningar av typen ”vita fingrar”. Den vibrationsbelastning som används uttrycks som 4-timmars ekvivalent frekvensvägd acceleration (A(4)) för den vibrationsriktning som uppvisar den högsta accelerationen. Denna standard användes fram till år 2001 då den reviderades (5, 6). I denna reviderade standard, ISO-5349 baseras skaderiskbedömningen på vektorsumman av de frekvensvägda accelerationsnivåerna för alla tre mätriktningarna beräknat över en arbetsdag på 8 timmar (A(8)).

Den modell för skaderiskbedömning som standarden ger (figur 2), bygger på ett samband mellan 8-timmars daglig exponering A(8) för HAV och blodflödes-störningar av typen ”vita fingrar”. Förslaget är baserat på antagandet att det finns ett samband mellan risk för skada och en kombination av vibrationsnivå och daglig exponeringstid, enligt figur 2. Standarden anger att skadeeffekterna i huvudsak antas vara ”vibrationsutlösta vita fingrar”. För övriga utfall presenteras inga samband. Denna riskbedömningsmodell är den som för närvarande tillämpas.

### Akuta effekter

Arbete med vibrerande maskiner har i experimentella studier rapporterats kunna medföra övergående förändringar i temperatur- (7) och beröringssinne (8), muskel- (9) och nervfunktion (10), reflexer samt cirkulation (11). Vid exponer-ingsvila upphör de akuta effekterna vanligen inom 15 minuter. Nuvarande kunskap ger inget stöd för att akuta effekter kvarstår längre än en timme (12). Intermittent vibrationsexponering under en hel arbetsdag medför dock en successiv övergående försämring av känselsinnet för vibrationer under dagens lopp (13). Tiden för full återhämtning påverkas bland annat av vibrations-exponeringens styrka, frekvens, och varaktighet samt den exponerades individuella känslighet.

Det är inte styrkt om graden av akutpåverkan kan användas vid bedömning av risk för bestående skada vid kraftig och långvarig vibrationsexponering.

Övergående nedsatt och förändrad känsel på grund av vibrationsinducerad blodflödesminskning och ändrad receptor- och nervfiberfunktion kan även leda till försämrad sensorimotorisk kontroll. Detta yttrar sig som svårigheter att utföra precisionsuppgifter, svårigheter att hantera maskiner, liksom en möjlig presta-tionsförsämring och en ökad risk för olyckshändelser. Vibrationsexponering kan även leda till en akut, övergående illusorisk proprioception med förändrad motorik som följd. Den störning som vibrationsexponering medför på nerv- muskelfunk-tionen, förblir vanligen omedveten för den exponerade (14).

### Bestående effekter

Längre tids exponering för handöverförda vibrationer från arbete med vibrerande maskiner har associerats med en ökad förekomst av symptom och skador på övre extremitetens vaskulära, neurologiska och muskuloskeletala system (15). Sammantaget anförs skadorna som ett hand-arm vibrationsskadesyndrom

(HAVS). Den vaskulära komponenten representeras av en ökad benägenhet för vasospasm i fingrarnas små kärl och manifesteras genom uppkomsten av ”vita fingrar” (Raynauds fenomen). Den neurologiska komponenten innefattar dels en diffus neurosensorisk skada, dels en skada med nervinklämning i handleden, ett så kallat karpaltunnelsyndrom (KTS). I båda fallen manifesteras symptomen av en störd nervfunktion. En ökad förekomst av muskelskada och påverkan på senor (bland annat Dupuytrens kontraktur) finns även rapporterat liksom skelettskador i form av artros.

## Motiv och övergripande syfte

Kunskap om samband mellan vibrationsexponering och skada bygger för närvarande enbart på beskrivande sammanställningar av vetenskapliga rapporter. För ”vita fingrar” används den riskbedömningsmodell som utgör ett annex till ISO-5349-1 (5). Neurosensorisk skada, karpaltunnelsyndrom, led- och muskelskador saknar alla separata riskbedömningsmodeller avseende vibrationsexponering. Det finns idag ingen evidensbaserad systematisk kunskapsöversikt över det vetenskapliga underlaget för hand-arm vibrationsskadesyndromet eller dess enskilda manifestationers samband med vibrationsexponering. Litteraturen innehåller inte heller någon statistisk syntes (meta-analys) av det vetenskapliga kunskapsunderlaget.

### Sambandet hand-arm vibration och Raynauds fenomen

Observationer om förhöjd förekomst av Raynauds fenomen (”vita fingrar”) har rapporterats sedan mer än 100 år tillbaka från ett flertal arbetsmiljöer där exponering för HAV förekommit (16-18).

”Vita fingrar-symptom” har i varierande omfattning tolkats som allt från en delkomponent i ett generellt utbrett syndrom till en specifik kärlfunktionsstörning med olika förslag på etiopatologisk bakgrund. Att symptomen orsakades av vibrationsexponering och att symptomen kunde utlösas av kyla befästes först under 1930-talet i samband med Seyring och medarbetares (19) studier på gjuteriarbetare. Alternativa samtidigt förekommande exponeringar (till exempel kvartsexponering, kyla) har även angivits kunna ligga bakom kärlskadan. Att arbete som medför vibrationsexponering utgör en möjlig risk för Raynauds fenomen finns väl dokumenterat, men det saknas kunskap om i vilken utsträckning vibrationsexponeringen i sig bidrar till Raynauds fenomen. Nuvarande riskbestämningsmodell för vita fingrar baseras på ISO-5349 och bygger på beräkningar av prevalenser och latenser från 7 studier publicerade under perioden 1946 till 1977. De studier som ingår rapporterar relativt höga exponeringar (A(8) ca 9 – 20 $m/s^2$) samtidigt som arbetstiden uppgick till mellan 9 och 12 timmar per dag och där exponeringen för vibrationer skedde mer eller mindre kontinuerligt med endast kortare avbrott. För lågdosområdet har risken extrapolerats utifrån de samband som påvisats för högre exponeringar (figur 2). De resultat som sambandet bygger på har hämtats i

första hand från studier inom gruvindustri (bergborrare), mekanisk industri (slipare) samt från skogsbruk med manuellt skogsarbete. Prevalensen av ”vita fingrar” i de bakomliggande studierna har varierat från 30% till 70%. Sambandet bygger även på en definition av ”vita fingrar” med varierande diagnostisk upplösning och precision. Följande systematiska kunskapsöversikt med åtföljande meta-analys försöker specifikt besvara frågan om hur stor risken är att drabbas av en förstärkt vasospasm (”Raynauds fenomen” eller ”vita fingrar”) i relation till exponering för handöverförda vibrationer.

### Sambandet hand-arm vibration och neurosensorisk skada

Observationer avseende perifer nervpåverkan i form av känselnedsättning, påverkan på motorik och nervsymptom rapporterades redan under slutet av 1900-talets andra decennium från arbetsmiljöer där exponering för HAV förekom (17). Nervskadan har, liksom kärlskada, i varierande omfattning tolkats som en allmän manifestation i ett utbrett syndrom till en specifik nervfunktionsstörning. De neurosensoriska symptomen tolkades fram till mitten av 1900-talet som ett tidigt tecken på, och som ett led i, utvecklingen av kärlskada. Kunskapen är idag otillräcklig för att kunna svara på i vilken utsträckning vibrationsexponeringen i sig bidrar till neurosensorisk skada, skild från arbetets övriga exponeringar. Följande systematiska kunskapsöversikt med åtföljande meta-analys försöker specifikt besvara frågan om hur stor risken är att drabbas av neurosensorisk skada i relation till exponering för handöverförda vibrationer.

### Sambandet hand-arm vibration och karpaltunnelsyndrom

Nervsymptom förenliga med de som kan ses vid karpaltunnelsyndrom rapporterades även tidigt från specifika arbetsmiljöer där exponering för vibrationer förekom (17). Samband mellan arbete med vibrerande verktyg och karpaltunnelsyndrom föreligger enligt ett flertal enskilda studier och har stöd även från systematiska kunskapsöversikter (till exempel (20) och (21)). Flertalet av originalrapporterna och de kunskapssammanställningar som undersökt sambandet mellan KTS och vibration tar emellertid inte hänsyn till vibrationsnivå utan indelar arbetet i om det innefattat exponering för vibrationer och möjligen under hur lång tid. Det saknas för närvarande kunskap om i vilken utsträckning vibrationsexponeringen i sig bidrar till ”karpaltunnelsyndrom”, om man tar hänsyn till arbetets övriga ergonomiska exponeringar. Följande systematiska kunskapsöversikt med åtföljande meta-analys försöker specifikt besvara frågan om hur stor risken är att drabbas av ”karpaltunnelsyndrom” i relation till exponering för handöverförda vibrationer.

# Metod

Denna systematiska kunskapsöversikt med åtföljande statistiska synteser (meta-analyser) avgränsas till hand-arm vibrationsexponering och kärl-, samt nervskada. Vi särredovisar nervskada, dels som neurosensorisk skada och dels som karpal-tunnelsyndrom. Hand-arm vibrationsskadesyndromets kärl- och nerv-manifestationer kan antingen uppträda var för sig eller tillsammans och utan inbördes relation. I följande kunskapsöversikt behandlas därför de olika utfallen separat. Endast studier där en mätning eller ett estimat på vibrationsexponeringen har rapporterats innefattas i kunskapsöversikten.

### Systematisk litteratursökning

Kunskapsgranskningen följer ”PRISMA statement” (Preferred Reporting Items for Systematic reviews and Meta-Analyses, www.prisma-statement.org) för rapportering av systematiska kunskapsöversikter och meta-analyser (22). Vi ansluter oss till PRISMAs riktlinjer avseende tillämpningen av dess 24-punkts checklista, terminologi, flöden och rapportering. Svensk terminologi för PRISMAs begrepp har hämtats från SBUs handbok för ”Utvärdering av metoder i hälso- och sjukvården” (23). Den systematiska litteraturgenomgången utgår enbart från vetenskapliga originalartiklar publicerade i refereegranskade tidskrifter.

De databaser som användes för sökningen var PubMed (U.S. National Library of Medicine, Bethesda, Maryland) och ScienceDirect (Elsevier, Amsterdam). Anledningen till de något överlappande databaserna var att de delvis indexerar artiklar från olika tidskrifter. Sökstrategin har medvetet varit så bred som möjligt för att inkludera artiklar med HAV exponering och utfall av funktionsstörning i kärl och nerver i hand/arm (i form av antingen blodflödesstörningar (Raynauds fenomen), påverkan på nerver (neurosensorisk skada) eller specifik nervpåverkan på medianusnerven i handleden (karpaltunnelsyndrom). Inledande sökning genomfördes brett, utan selektionskriterier baserade på indexering. För att inkluderas skulle studierna:

- Innehålla mätningar eller estimat av vibrationsexponering.
- Innehålla relevant hälsoutfall.
- Innehålla originaldata (ej review).
- Syfta till att studera risk för skada.
- Vara publicerade på engelska.
- Studera effekter på människa.

Artiklar publicerade på annat språk än engelska respektive studier som inte avsåg effekter på människa exkluderades manuellt. Sök-strategier finns presenterade i bilaga 1. Litteratursökningen omfattar publikationer från 1945 fram till den 31 september 2014.

Artiklarnas sammanfattningar granskades med avseende på relevans för litteraturgenomgångens syfte med krav på uppgifter om vibrationsexponering samt aktuella hälsoutfall. Samtliga artiklar granskades av två av författarna

oberoende av varandra. Vid oenighet diskuterade alla tre författare respektive artikel till dess att konsensus uppnåddes.

För varje artikelsammanfattning som befanns relevant granskades sedan den fullständiga publikationen av två granskare var för sig för att säkerställa att relevanskriterierna uppfyllts. Vid granskningen användes ett protokoll med uppställda kvalitetskriterier med poäng (bilaga 2) som fokuserade på studiernas metod och vetenskapliga kvalité i betydelsen risk för bias. Varje fullständig artikel diskuterades av de två granskarna och vid oenighet fördes diskussionen vidare till samtliga tre granskare för beslut i konsensus.

### Resultat av den övergripande systematiska litteratursökningen

Vi fann totalt 4325 sammanfattningar (figur 3), som lästes och vilkas relevans bedömdes enligt de i förväg uppsatta kriterierna. 293 artiklar granskades sedan i sin helhet för att avgöra om respektive artikel uppfyllde inklusionskriterierna. Av de 293 artiklarna uteslöts 241 (figur 3), vilket resulterade i att 52 artiklar slutligen uppfyllde de i förväg uppställda kriterierna för inklusion i den systematiska översikten.

Figur 4 visar antalet artiklar och överlappning mellan artiklar som belyser Raynauds fenomen, neurosensorisk skada och karpaltunnelsyndrom i relation till vibrationsexponering. En *artikel* kan innehålla analyser av flera hälsoutfall vilket benämns *studie*, detta innebär att det totala antalet artiklar är 52 och det totala antalet *studier* 81. I varje studie kan dessutom flera yrkesgrupper ingå. För utfallet Raynauds fenomen erhölls 41 studier, för neurosensoriskt utfall 33 studier och för utfallet karpaltunnelsyndrom 7 studier. Det innebär att bland de 52 artiklarna behandlade 24 både Raynauds fenomen och neurosensorisk skada, 2 behandlade både neurosensorisk skada och KTS, 17 artiklar studerade enbart Raynauds fenomen, medan motsvarande för neurosensorisk skada och KTS var 7 respektive 2 artiklar (figur 4).

De 52 artiklar som uppfyllde kraven på att ingå i den systematiska kunskaps-genomgången utgör undersökningar utförda huvudsakligen i den industrialiserade delen av världen (figur 5).

Mer än 2/3 av de artiklar som ingår i kunskapsöversikten kommer från Italien, Sverige eller Japan (tabell 1).

Tjugofem artiklar är publicerade före år 1997 och 27 artiklar därefter. Artiklar fokuserade på kärl- och nervskador har förekommit under perioden från 1978 till 2014, medan studier som belyser frågan om vibrationsexponering och karpal-tunnelsyndrom tillkommit först från mitten av 1990-talet och framåt (figur 6).

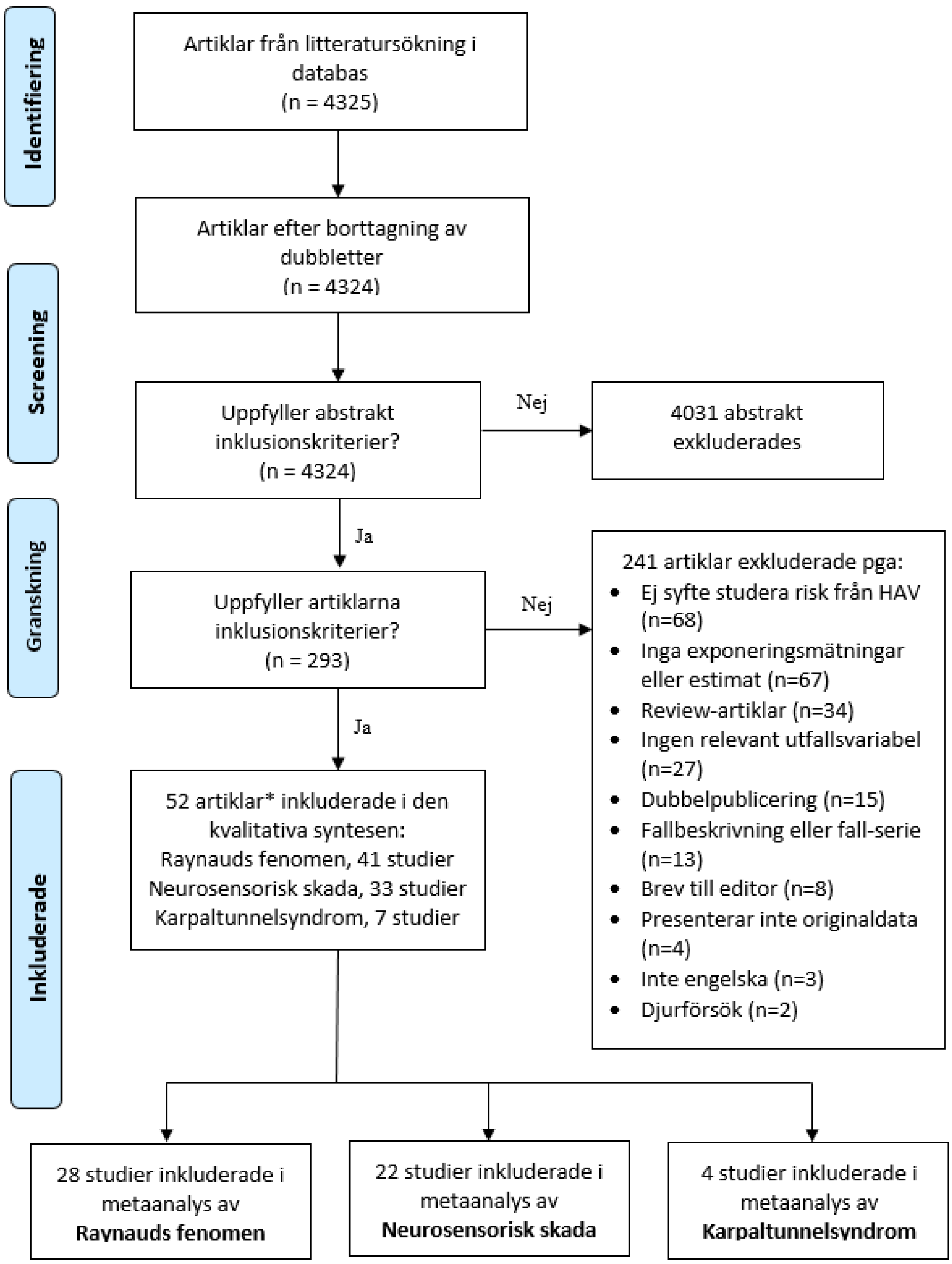


**Figur 3:** Flödesschema för sökstrategi och urval av studier för att utvärdera sambandet mellan hand-arm vibrationer och hälsoutfall (Raynauds fenomen, neurosensorisk skada, karpaltunnelsyndrom) i enlighet med PRISMA. * En artikel kan innehålla analyser av flera hälsoutfall, vilket benämns studie, och detta innebär att det totala antalet artiklar är 52 och det totala antalet studier 81.

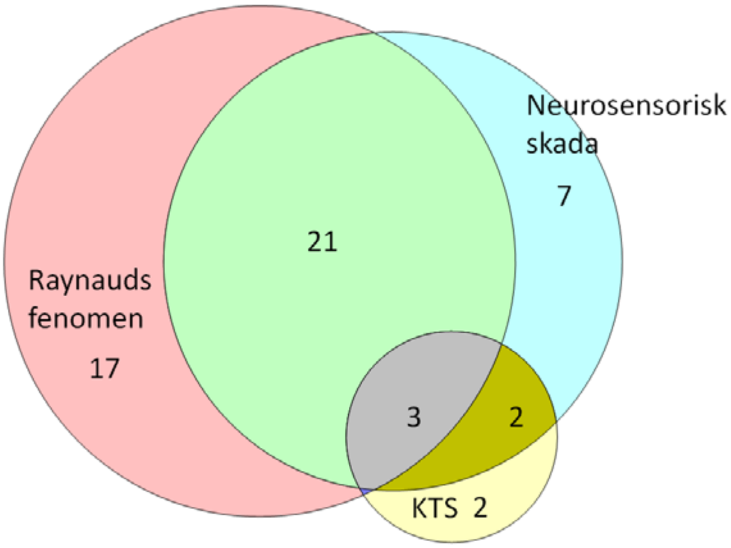


**Figur 4:** Venn-diagram över antalet artiklar som specifikt belyser Raynauds fenomen, neurosensorisk skada samt karpaltunnelsyndrom (KTS) i relation till vibrationsexponering.

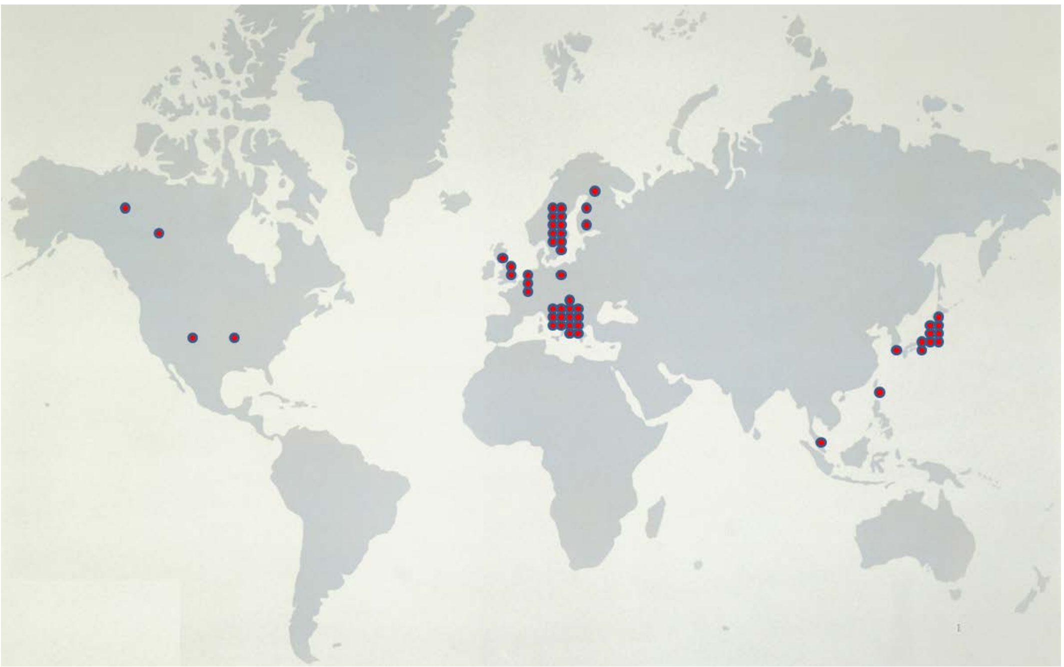

**Figur 5:** Världskarta med markering för de länder från vilka ingående artiklar i kunskapsöversikten kommer.

**Tabell 1:** Antal artiklar och procentuell andel av totala antalet artiklar som ingår i kunskapsöversikten i relation till publiceringsland.

| Land | Antal | Procent |
|---|---|---|
| Belgien | 1 | 1,9% |
| Finland | 3 | 5,8% |
| Italien | 15 | 28,8% |
| Japan | 9 | 17,3% |
| Kanada | 2 | 3,8% |
| Kina | 1 | 1,9% |
| Malaysia | 1 | 1,9% |
| Nederländerna | 2 | 3,8% |
| Polen | 1 | 1,9% |
| Storbritannien | 3 | 5,8% |
| Sverige | 11 | 21,2% |
| Sydkorea | 1 | 1,9% |
| USA | 2 | 3,8% |
| **Summa** | **52** | **100,0%** |

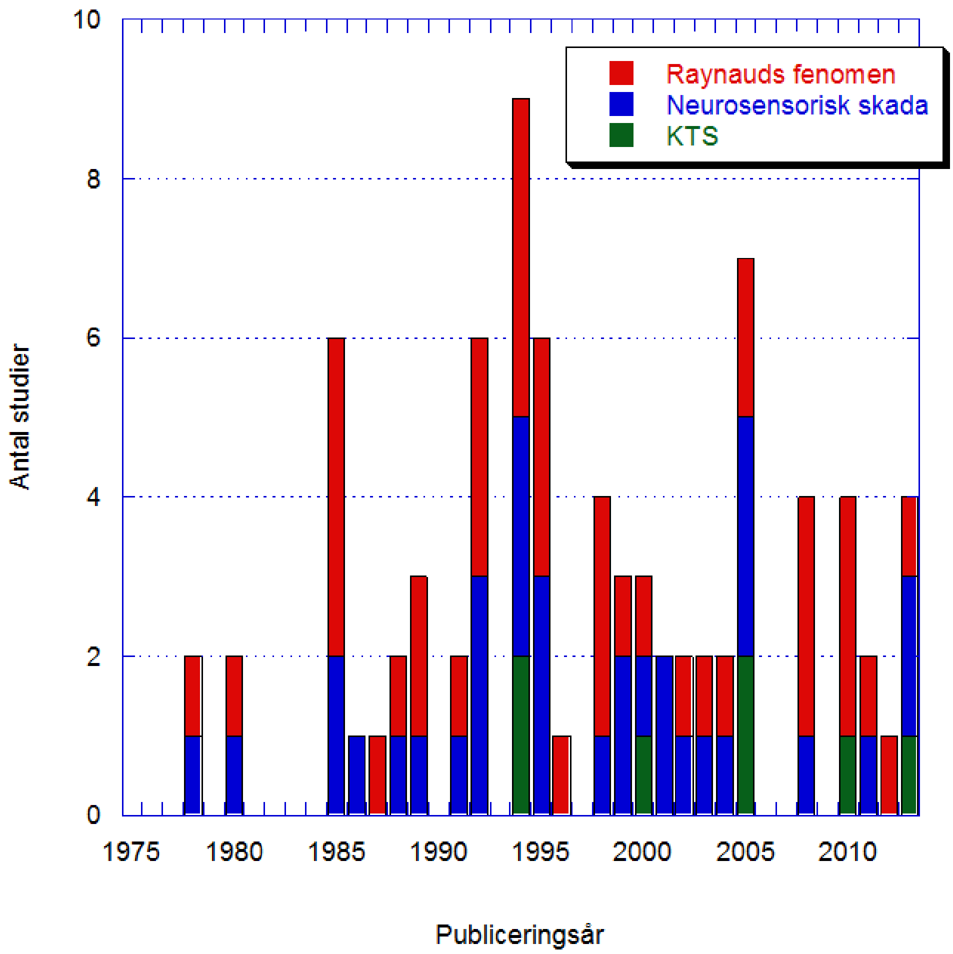


**Figur 6:** De 81 studier som belyser Raynauds fenomen, neurosensorisk skada och karpaltunnelsyndrom i relation till publiceringsår.

## Branscher och uppmätta exponeringsnivåer

Den största andelen av de ingående artiklarna har genomförts inom verkstads-industri, skogsindustri samt inom gruv- och stenindustri. Sammantaget står dessa tre branscher för mer än 75 % av artiklarna som inkluderats i denna samman-ställning (figur 7). Notera att det i artiklarna kan ha ingått flera olika yrkesgrupper där exponeringsnivåer uppmätts varför det totala antalet som presenteras i figur 7 uppgår till 63. Endast i ett fåtal artiklar har kvinnor inkluderats i den studerade populationen (24-27).

*Verkstadsindustri*

Vanligt använda maskiner, för vilka vibrationer uppmätts inom verkstads-industrin, är slipmaskiner av olika slag, men även mutterdragare, mejselhammare och nithammare. Medianvärdet från de artiklar och yrkesgrupper (n=20) där det var möjligt att beräkna ett A(8) värde var 2,8 m/s$^2$ med en spridning från 0,6-17,4 m/s$^2$ (figur 7).

*Skogsindustri*

Från skogsindustrin finns framför allt vibrationsmätningar från motorsågar men i enstaka fall har också mätningar gjorts på röjsågar. Det finns äldre mätningar från motorsågar som inte var avvibrerade och från nyare med avvibrering, där nivåerna är väsentligt lägre. Medianvärdet från de artiklar och yrkesgrupper (n=7) där det var möjligt att räkna fram ett A(8) värde, var 3,5 m/s$^2$ med en spridning från 2,4-5,4 m/s$^2$ (figur 7).

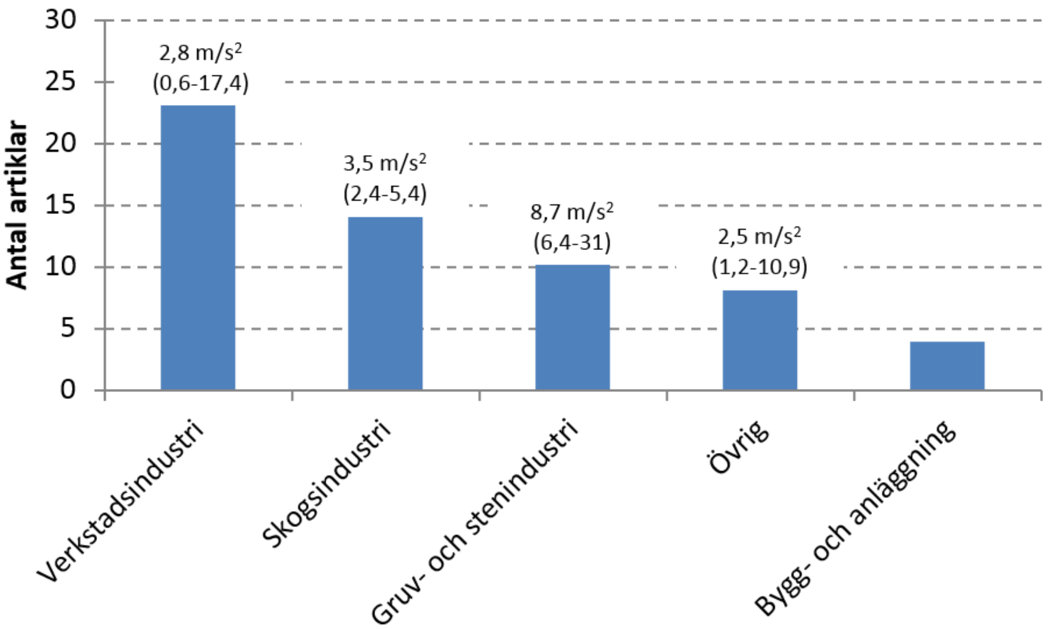


**Figur 7:** Figuren visar antalet artiklar och yrkesgrupper från olika branscher där vibrationsexponering (uttryckt som 8-timmarsekvivalent) [A(8)] kunnat beräknas. Utöver antalet redovisas också medianvärdet för vibrationsexponeringen och spridningen (min- och maxvärde inom parentes). För bygg- och anläggning anges inga exponeringsnivåer då enbart en av de fyra studierna byggde på direkta mätningar.

*Gruv- och stenindustri*
Maskiner som används inom gruv- och stenindustri är framför allt bergborrar och stora slående mejselhammare, men även slip- och kapmaskiner används i vissa sammanhang. Medianvärdet från de artiklar och yrkesgrupper (n=9) där det var möjligt att räkna fram ett A(8) värde, var 8,7 $m/s^2$ med en spridning från 6,4-31,0 $m/s^2$ (figur 7).

*Bygg- och anläggningsindustri*
Av studier inom bygg- och anläggningsindustri som inkluderats i denna översikt är två genomförda i Sverige (28, 29), en i Italien (30) och en i Malaysia (31). De enda mätningar som genomförts är från Italien i slutet på 1990-talet och det värde som presenterades för vibrationsexponering räknat över åtta timmar (A(8)) var 2,8 $m/s^2$ (30).

*Övriga industrier*
De artiklar som klassats i gruppen ”övriga industrier” innefattar bland annat möbeltillverkning (24), renskötande samer som exponerats för vibrationer via handtag på snöskoter (32) och två olika artiklar av distributionspersonal inom gasindustrin (33, 34). Medianvärdet från de artiklar där det var möjligt att räkna fram ett A(8) värde var 2,5 $m/s^2$ med en spridning från 1,2-10,9 $m/s^2$.

## Metaanalys

Studier som rapporterade en relativ risk (oddskvot) för något av de tre utfallen (Raynauds fenomen, neurosensorisk skada samt KTS) innefattades i våra respektive metaanalyser. Vidare inkluderades i metaanalyserna studier som presenterat uppgifter vilka möjliggjorde beräkning av en ojusterad oddskvot.

För att studera hur vibrationsexponeringens nivå påverkar utfallen, har studier på likartade grupper av vibrationsexponerade arbetare, men med olika exponeringsnivåer, analyserats. Den lägsta exponeringsgruppen definierades som "lågexponerad" och den högst exponerade gruppen som "högexponerad". Jämförelserna mellan olika riskestimat har gjorts enligt Altman och Bland (35). Exponeringsuppdelningen baseras på de exponeringsuppgifter som kunnat identifieras i respektive artikel. Följande fyra doser har identifierats: Dos 1 = Antal exponeringsår (år); Dos 2 = Antal exponeringstimmar (tim); Dos 3 = Daglig vibrationsexponering, A(8) ($m/s^2$); Dos 4 = Kumulerad vibrationsexponering ($mh/s^2$, $m^2h/s^4$, $m^2h^3/s^4$).

Vi genomförde ”random-effect” metaanalyser och testade samstämmighet (heterogenitet) mellan de olika studierna med hjälp av Cochrans chi-två (Q-test) och $I^2$ statistik (36, 37). Risken för bias bedömdes genom kumulativ metaanalys och genom subgruppsanalys. För kumulativ metaanalys var studierna rankade i fallande ordning efter deras respektive kvalitetspoäng (risk för bias). I resultatet redovisas för varje studie effektstorleken som oddskvot med 95 % konfidensintervall (KI) samt studiens relativa betydelse i analysen. I de fall tomma celler

förekom (endast för kärlskada), har vi lagt till ett fall till de tomma cellerna, samt ett fall i övriga celler för att möjliggöra beräkning av riskestimaten (41).

Publikationsbias undersöktes med trattdiagram (”funnel plots”). Asymmetri i trattdiagrammen bedömdes med tre statistiska metoder: rang korrelationsmetoden (38), regressionsanalys (39) och med Duval och Tweedie ”trim and fill” metod (40).

Av ingående 81 studier har 54 inkluderats i meta-analyser (figur 3). Exkluderingen betingas bland annat av att vissa studier enbart beskrivit prevalensen av skada hos de vibrationsexponerade (42-50), eller att syftet varit att jämföra exponerade med oexponerade avseende något specifikt kliniskt utfall till exempel temperaturtrösklar (27, 29, 51-54). Dessutom förekommer studier som beskriver samma studiepopulation och med snarlika uppgifter (55, 56). Vid dubbleringar har endast den publikation som bedömts vara mest relevant inkluderats i metaanalys.

Alla beräkningar har gjorts med statistikprogrammet CMA Comprehensive Meta-Analysis Version 3.3 (Biostat, Englewood, USA; (57)).

## Raynauds fenomen (”Vita fingrar”)

### Definition

Den medicinska termen ”Raynauds fenomen” beskriver en övergående attack med försämrad cirkulation i huden och där huden upplevs som ”död” och ”kall” (”vita fingrar”). Manifestationen uppkommer som följd av en förstärkt spasm i hudens små perifera blodkärl.

Kliniskt utmärks Raynauds fenomen av en avblekning distalt på fingrar och där gränsen mellan normal och avblekt vävnad framträder tydligt. Manifestationerna visar sig vid nedkylning eller under stress. En attack med Raynauds fenomen innebär störd perifer blodcirkulation i hudens små kärl, vilket i sig medför en övergående sänkning av hudtemperaturen över det ”vita området” samtidigt som känselsinnet och den finmotoriska förmågan blir nedsatt.

Efter attacken med ”vita fingrar” återfår huden normal färg och temperatur. Vid återgången skiftar ibland hudens färg till rödviolett och klarrött. När en längre tids attack med kärlspasm upphör syresätts åter vävnaden och svår smärta kan upplevas.

### Diagnostisk tillförlitlighet och risk för bias

Nedan följer en detaljerad beskrivning av begreppet ”vita fingrar” och dess begreppsutveckling med hänsyn tagen till hur det använts under olika tidsperioder. Detta motiveras av att litteratursökningen spänner över studier vitt åtskilda i tid, allt från 1945 fram till 2014 och där de slutligen selekterade vetenskapliga rapporterna publicerats från 1978 till 2013. Definitionen av ”vita fingrar” och klassificeringen av dess allvarlighetsgrad har genomgått betydande revideringar under denna tidsperiod.

Symptomen ”vita fingrar” och dess medicinska motsvarighet Raynauds fenomen är kollektivnamnet för både primär (Raynauds sjukdom) och sekundär vasospasm (Raynauds syndrom) (58). Raynauds fenomen betraktas som primär om attackerna uppkommer utan att det framkommit hållpunkter för annan sjukdom eller annan känd orsak. Vid sekundärt Raynauds fenomen förmodas etiologin vara känd. De vanliga orsakerna till sekundärt Raynauds fenomen är medicinering, reumatisk sjukdom, anatomiska särförhållanden och toxiska effekter samt vibrationsexponering (detaljerad beskrivning ges i bilaga 3).

I de fall där det föreligger attacker med entydigt avgränsad, tydlig avblekning som vid Raynauds fenomen (*kliniskt kriterium*) i fingrar eller tår utlöst av kyla eller annan stressor (*trigger kriterium*) och där hand-arm överförd vibrationsexponering (*exponeringskriterium*) föregått sjukdomsdebuten (*tidssambandskriterium*), samtidigt som det inte framkommit någon annan känd faktor som kan ge Raynauds fenomen (*exklusionskriterium*), kan diagnosen ”vibrationsutlöst Raynauds syndrom” eller ”vibrationsutlösta vita fingrar” övervägas.

Minst en episod med ”Raynauds fenomen” skall ha förekommit någon gång under de senaste 2 åren för att aktivt Raynauds syndrom skall betraktas föreligga (*aktivt syndrom kriteriet*). Vid längre tid än 2 år i remission bedöms Raynauds syndrom ha gått i regress.

Objektiv undersökning av symptom på Raynauds fenomen har i enstaka studier innefattat bestämning av det kritiska öppningstrycket (59) vid köldprovokation (”critical opening pressure” COP) (60). En minskning av blodtrycket i testfingret till mindre än 60 % av referensblodtrycket används som tecken på att reaktionen på kyla är förstärkt (61). COP-testets sensitivitet är förhöjd hos nikotinanvändare, vilket innebär att ett negativt testresultat hos icke tobaksbrukare har ett lägre prediktivt värde. COP-undersökning krävs ej för diagnosen Raynauds fenomen i de fall där det föreligger en entydig, typisk, beskrivning av symptomen (62). Registrering av återuppvärmning är ytterligare ett alternativt objektivt test på reaktionen vid köldprovokation. Testet innebär mätning av hudtemperatur under återuppvärmningsfasen med registrering av hastigheten för normalisering av hudtemperaturen efter en köldprovokation (63).

## Gradering av ”kärlskadans” allvarlighetsgrad

Gradering av Raynaud-fenomenets allvarlighetsgrad har i varierande omfattning utgått från symptomens karaktär (vasospasm), utbredning (antal fingrar, falanger), svårighetsgrad (besvär), hur frekvent symptomen uppträder (attackfrekvens), hur lätt symptomen utlöses (vid vilken temperatur, sommar, vinter), duration (attackernas duration) och återhämtningstid. Attackernas påverkan på det sociala livet respektive dess påverkan på arbetet (arbetsförmåga, arbetshinder) har även vägts in vid graderingen.

En preciserad gradering krävs för att medge bedömning av allvarlighetsgrad, förloppsutveckling, prognos samt vid utvärdering av effekterna från förändrad exponering. En gradering av skadans allvarlighetsgrad styr även storleken på den ekonomiska ersättningen vid arbetsskada

Den tidigaste graderingen klassificerade skadan i fyra stadier utifrån enbart ”allvarlighetsgrad” (tabell 2) och utan hänsyn tagen till om manifestationen representerade kärl- eller nervpåverkan (64). Klassificeringen byggde på antagandet om en generell, gemensam bakomliggande patologi för ”vibrations-sjukdomen”. ”Vibrationssjukdomen” ansågs kunna uppstå lika oavsett om vibrationerna kom från hand-arm överförda exponeringar eller från helkropps-överförda exponeringar.

Andreva och Gelinas skala omarbetades och integrerades sedermera i en ny skala, där typen av manifestation (Angiodystoniskt syndrom, Angiospastiskt syndrom, Vegetativt polyneurit syndrom, Vegetativt myofaschit syndrom, Neurit, Diencephalon syndrom och Vestibulärt syndrom) utgjorde utgångspunkt för kategoriseringen (66). Dessa skalor användes främst i de forna öststaterna. Ingen studie med denna klassificering uppfyllde dock kraven för att ingå i denna kunskapsöversikt.

Inom det västliga vetenskapssamhället styrde kulturella, medikolegala och medicinska traditioner graderingen mot en klassificering byggd på vibrations-skadesyndromets symptom och dess interferens med arbete och sociala aktiviteter (Taylor-Pelmear-skalan, tabell 3) (67, 68).

Taylor-Pelmears klassificeringsskala omarbetades i samband med ett möte i Stockholm 1986. Vid detta möte (Stockholm Workshop) om ”Symptomatology and diagnostic methods in the hand-arm vibration syndrome” konstaterades att HAVS kunde omfatta kärlskada eller nervskada, antingen var för sig eller till-sammans. Taylor-Pelmear skalan ersattes därefter av två separata skalor. En för kärl- (69) och en för nervskada (70). I 1987 års klassificeringsskala för ”vita fingrar” togs skattningen av hur mycket symptomen interfererar med fritid och arbete bort och skattningen kom i första hand att baseras på frekvens- och utbredningskriterier. Vid en förnyad workshop i Stockholm 1994 reviderades skalan för vita fingrar, utan större förändringar. Flertalet studier har enbart angett symptom som ”vita fingrar”, i andra har symptomen kompletterats med en klassificering. I de artiklar som omfattas i denna kunskapssammanställning har klassificering enligt Taylor-Pelmear-skalan och Stockholm Workshop-skalan använts i 10 respektive 14 av artiklarna.

**Tabell 2:** Klassificering av vibrationsskada i 4 stadier, enligt Andreeva-Galanina. Fritt översatt från originalet (65).

| Stadium | Beskrivning |
|---|---|
| 1 | Tidigt, helt reversibelt stadium |
| 2 | Tydliga, men måttligt markerade symptom |
| 3 | Uttalad patologi |
| 4 | Varaktigt och slutgiltigt stadium med uttalad minskning eller till och med förlust av arbetsförmåga |

Stockholm Workshop-skalan anger att antalet drabbade fingrar i olika stadier skall redovisas för vardera handen, var för sig, till exempel ”2L(2), 1R(1)”. Det innebär i detta exempel att 2 fingrar på vänster hand är drabbade av Raynaud-fenomen i stadium 2, och att skadan berör 1 finger på höger hand, med svårighetsgraden ”mild”, det vill säga stadium 1.

Griffin föreslår i sin ”Handbook of Human vibration” (71) en alternativ skala för gradering av vita fingrar, som väger samman antalet falanger drabbade av vasospasm. Denna skala har endast använts i två av de artiklar som ingår i översikten.

**Tabell 3:** Symptomklassificering för Raynaud-fenomen hos vibrationsskadade enligt Taylor-Pelmear-skalan från 1975. Fritt översatt från originalet (67).

| Stadium | Fingertillstånd | Inverkan på social aktivitet och yrke |
|---|---|---|
| 0 | Ingen avblekning | Inga besvär |
| $0_T$ | Intermittenta stickningar (”tingling”). | Ingen inverkan på aktiviteter |
| $0_N$ | Intermittent domning | Ingen inverkan på aktiviteter |
| 1 | Avblekning av en fingertopp eller flera fingertoppar med eller utan stickningar och domning. | Ingen inverkan på aktiviteter. |
| 2 | Avblekning av ett helt finger eller flera fingrar, med domning. Vanligen begränsad till vintertid. | Lätt inverkan på sociala aktiviteter. Ingen inverkan på arbetet. |
| 3 | Utbredd avblekning. Ofta förekommande attacker, sommar såväl som vinter. | Klar inverkan på yrkesverksamhet och sociala aktiviteter. Inskränkning i hobbyverksamhet. |
| 4 | Utbredd avblekning på de flesta fingrarna. Ofta förekommande attacker sommar och vinter. | Symptomens svårighetsgrad har lett till arbetsbyte för att undvika ytterligare vibrationsexponering. |

**Tabell 4:** Stockholm Workshop-skalan för klassificering av vibrationsinducerade Raynaud-fenomen utlösta av kyla. Fritt översatt från originalet (69).

| Stadium | Svårighetsgrad | Beskrivning |
|---|---|---|
| 0 | - | Inga anfall av vita fingrar |
| 1 | Mild | Anfall då och då, omfattande endast ytterfalangen på ett finger eller flera fingrar |
| 2 | Medelsvår | Anfall då och då, omfattande ytter- och mellanfalangerna (sällan också den proximala falangen) på ett finger eller flera fingrar |
| 3 | Svår | Anfall ofta, omfattande alla falanger på de flesta fingrar |
| 4 | Mycket svår | Som stadium 3 men med trofiska hudförändringar i fingertopparna |

### Möjlig bias till följd av alternativa orsaker till Raynauds fenomen

Numera anges mer än 40-talet olika orsaker kunna ge upphov till sekundärt Raynauds fenomen (72, 73). De vanligaste orsakerna till sekundärt Raynauds fenomen i en allmänbefolkning är enligt Brandt och medarbetare användande av betablockerande medicinering följt av nervkompression och immunologisk sjukdom (74). Till de kända orsakerna till sekundärt Raynauds fenomen hör förutom ovanstående även autoimmuna sjukdomar, rheologiska förändringar, vaskuliter, läkemedel, kemiska yrkesexponeringar, kärlobstruktiva respektive kärlskadande sjukdomar, nervskador samt hormonell påverkan, (detaljerad beskrivning framgår av bilaga 3).

### Möjlig bias till följd av interaktion från andra exponeringsfaktorer

Nikotin har en vasoaktiv effekt och kan förväntas samverka med effekter från vibrerande maskiner på vasospastiska symptom. Cherniack och medarbetare har följt upp arbetare som upphört med sin vibrationsexponering (n=199) (75) och analyserat utfallet på vita fingrar i relation till tobaksbruk. De fann att symptom förekom oftare bland tobaksbrukare samt att utfallet på köldprovokationstest visade tydligare patologiska värden bland tobaksbrukare jämfört med ej tobaksbrukande. Vid undersökning efter två år utan vibrationsexponering hade tobaksbrukare dubbelt så hög förekomst av Raynauds fenomen (32,2 % vs 17,4) jämfört med de som ej rökt. Motsvarande resultat finns rapporterade i ett flertal likartade studier (76, 77).

Kyla interagerar även med ”vita fingrar” symptom. Kyla kan i sig ge upphov till ökad benägenhet för vasospasm (efter förfrysning), men kyla är även en trigger för att utlösa attacker (28).

### Resultat av den systematiska litteratursökningen för Raynauds fenomen

Resultatet av den systematiska litteraturgenomgången presenteras nedan i tabellform (tabell 5) för de studier som behandlar kärlpåverkan. Av tabellen framgår dels vår skattning av risk för bias (kvalitetspoäng) avseende diagnosens tillförlitlighet för Raynauds fenomen samt dels den totala summan av kvalitetspoäng. Totalsumman är summan av kvalitetspoängen för diagnos adderad med kvalitetspoängen för ”studie-metod” och ”exponering”.

För utfallet Raynauds fenomen erhölls 41 studier som publicerats mellan åren 1978 och 2013. Sammanställningen i tabell 5 visar att de 41 studier som behandlat kärlpåverkan, har en variation av diagnossumma mellan 1 och 11 för diagnostisk tillförlitlighet av Raynauds fenomen.

*Prevalens av Raynauds fenomen i relation till publiceringsår*
I de 41 studierna finns unika uppgifter redovisade i 35 studier om prevalens av Raynauds fenomen bland vibrationsexponerade. I 4 av studierna ingår uppgifter om prevalens bland flera grupper av exponerade vilket resulterar i att det totalt

**Tabell 5:** Ingående studier på Raynauds fenomen (n=41) samt deras bedömda risk för bias (kvalitetspoäng) avseende diagnosen ”Raynauds fenomen” (Diagnos summa) samt totalsumma när kvalitetspoängen för bedömningen diagnos ”Raynauds fenomen, ”studie metod” och ”exponering” adderats (Total summa). Studierna presenteras i fallande ordning utifrån den totala poängsumman. Högre poäng indikerar högre ”kvalitet” vilket indikerar mindre möjlig risk för bias. Vidare anges studiedesign för respektive studie.

| Studie | Referens | Design | Diagnos summa | Total summa |
|---|---|---|---|---|
| Bovenzi, 1998 | (55) | Kohort | 11 | 29 |
| Bovenzi, 2008 | (56) | Kohort | 11 | 28 |
| Bovenzi, 2008* | (78) | Kohort | 11 | 27 |
| Bovenzi, 2000 | (79) | Fall-kontroll | 11 | 26 |
| Bovenzi, 2010* | (80) | Kohort | 9 | 26 |
| Bovenzi, 1995* | (81) | Tvärsnitt | 11 | 25 |
| Bovenzi, 2010* | (82) | Kohort | 9 | 25 |
| Bovenzi, 1998* | (30) | Tvärsnitt | 11 | 24 |
| Mirbod, 1992* | (83) | Fall-kontroll | 11 | 23 |
| Bovenzi, 2011* | (84) | Kohort | 8 | 23 |
| Mirbod, 1999 | (49) | Kohort | 8 | 22 |
| Aiba, 2012 | (42) | Kohort | 8 | 21 |
| Hagberg, 2008* | (85) | Kohort | 4 | 21 |
| Barregard, 2003 | (43) | Tvärsnitt | 8 | 20 |
| Bovenzi, 1988* | (86) | Tvärsnitt | 10 | 19 |
| Bovenzi, 1985 | (87) | Tvärsnitt | 9 | 19 |
| Bovenzi, 1994* | (88) | Tvärsnitt | 8 | 19 |
| Nilsson, 1989* | (89) | Tvärsnitt | 7 | 19 |
| Palmer, 1998* | (33) | Tvärsnitt | 7 | 18 |
| Futatsuka, 2005* | (90) | Tvärsnitt | 7 | 17 |
| Brubaker, 1987* | (44) | Kohort | 6 | 17 |
| Koskimies, 1992 | (48) | Tvärsnitt | 6 | 17 |
| Futatsuka, 1985 | (45) | Kohort | 2 | 17 |
| Su, 2013 | (31) | Tvärsnitt | 6 | 16 |
| Jang, 2002* | (91) | Tvärsnitt | 4 | 16 |
| Yamada, 1995* | (92) | Tvärsnitt | 9 | 15 |
| Bovenzi, 2005* | (24) | Tvärsnitt | 6 | 15 |
| Letz, 1992* | (93) | Tvärsnitt | 4 | 15 |
| Chatterjee, 1978* | (94) | Tvärsnitt | 6 | 14 |
| Harazin, 1996 | (47) | Tvärsnitt | 6 | 14 |
| Brubaker, 1985* | (95) | Tvärsnitt | 5 | 14 |
| Burdorf, 1991* | (96) | Tvärsnitt | 3 | 14 |
| Cherniack, 2004* | (97) | Tvärsnitt | 3 | 14 |
| Anttonen, 1994* | (32) | Tvärsnitt | 1 | 12 |
| Virokannas, 1995* | (98) | Tvärsnitt | 1 | 12 |
| Bovenzi, 1980* | (99) | Tvärsnitt | 2 | 10 |
| Musson, 1989 | (50) | Tvärsnitt | 1 | 10 |
| Walker, 1985* | (34) | Tvärsnitt | 1 | 9 |
| Burstrom, 2010* | (28) | Tvärsnitt | 1 | 8 |
| Mirbod, 1994* | (26) | Tvärsnitt | 1 | 7 |
| Tominaga, 1994* | (100) | Tvärsnitt | 1 | 7 |

*Studien inkluderad i meta-analys

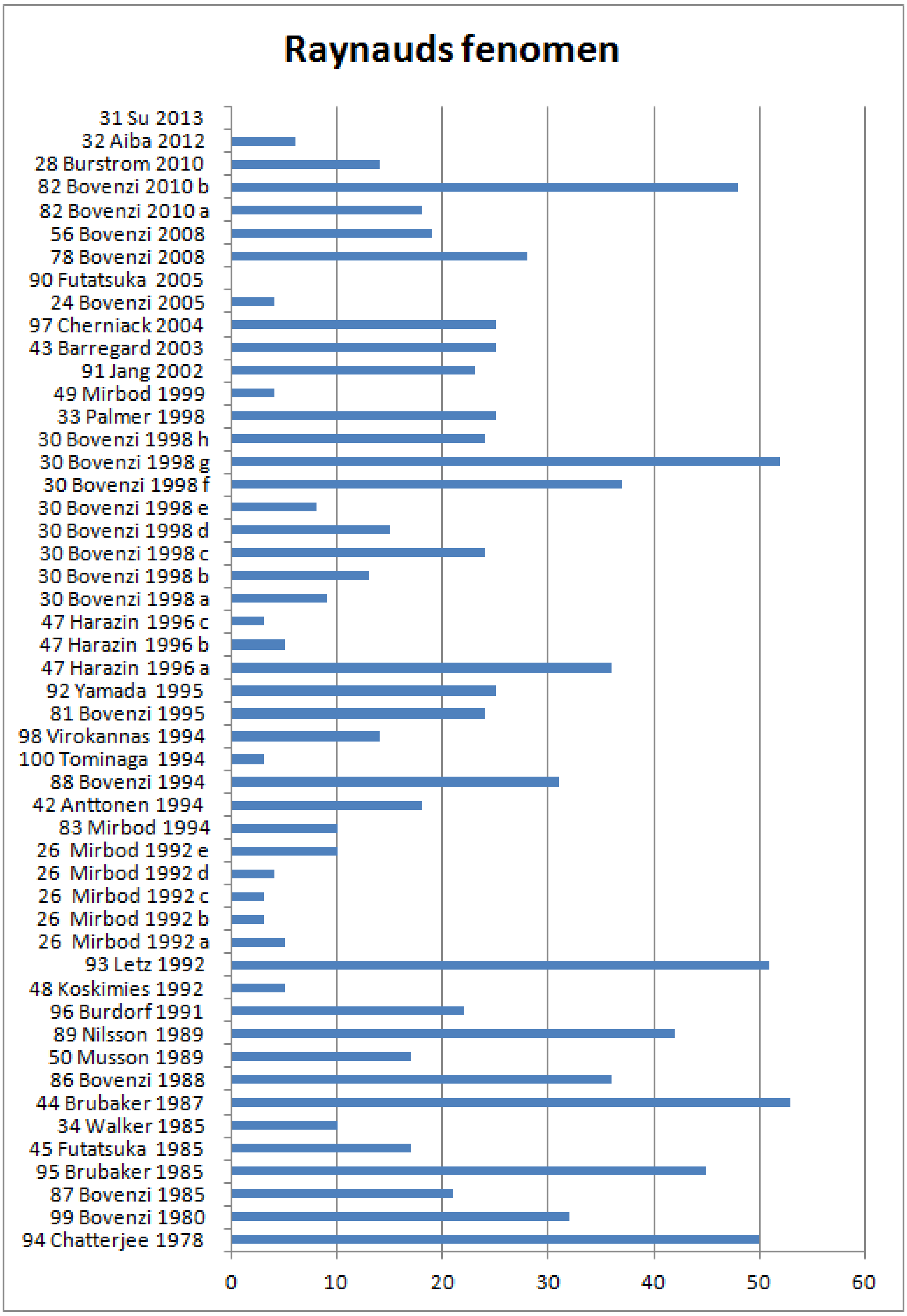


**Figur 8:** Studier med uppgift om prevalens av Raynauds fenomen i den undersökta studiepopulationen sorterade efter publiceringsår.(n=50).

finns prevalensuppgifter från 50 yrkesgrupper som presenteras i figur 8 sorterade efter publiceringsår.

Figur 8 visar att prevalensen för Raynauds fenomen varierar från 0% till 53% bland de olika studerade yrkesgrupperna med ett genomsnitt på 20%.

*Prevalens av Raynauds fenomen i relation till vibrationsexponering A(8)*
I 33 av studierna finns uppgifter om vibrationsexponeringen beskriven som daglig ekvivalent vibration A(8) eller med uppgifter som möjliggjort beräkningar av A(8). I vissa av studierna har dessutom flera grupper av exponerade ingått vilket ger ett totalt antal uppgifter på 48 där både prevalens och exponering rapporterats. I figur 9 nedan har sambandet mellan A(8) och prevalens av Raynauds fenomen sammanställts.

Figur 9 visar att prevalensen varierar mellan 0% till drygt 50% (medel 18%; median 15%) medan A(8) varierat från 0,6 m/s2 till ca 30 m/s2 (medel; 5,6 m/s2; median 3,6 m/s2). Linjär regression visar att för varje fördubbling av A(8) ökar prevalensen av Raynauds fenomen med ca 5 procentenheter. I 28 studier har även den totala medelvärdiga exponeringstiden för vibrationer i gruppen redovisats och den varierar mellan 4 år och drygt 25 år (medel 15 år; median 15 år).

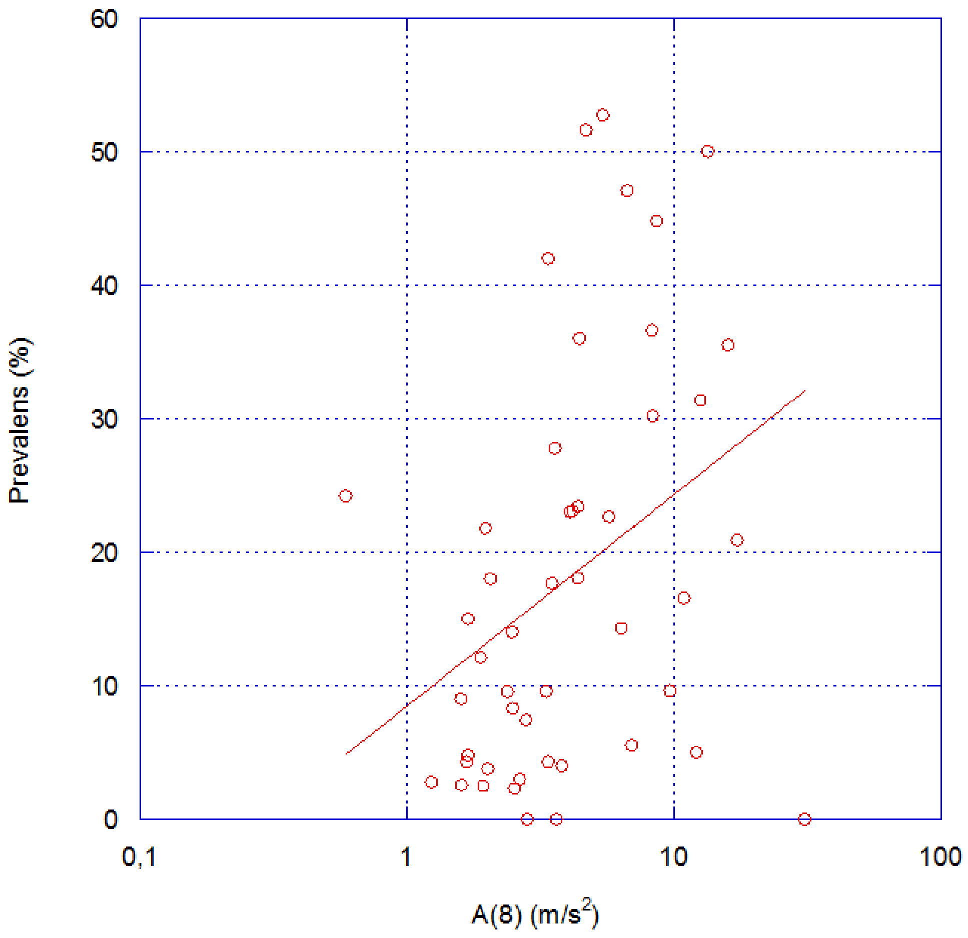


**Figur 9:** Prevalens (%) av Raynauds fenomen i relation till daglig vibrationsnivå A(8) (n=48) samt den linjära regressionslinjen (Ekvation: Prevalens (%)=8,5+15,8*$\log_{10}$(A(8)); r=0,36).

**Resultat av meta-analys från studier på ”Raynauds fenomen”**

Meta-analysen har delats upp dels mellan exponerade och icke-exponerade dels mellan "lågexponerad" och "högexponerad". I meta-analysen har totalt ingått 28 studier, 22 i den första analysen och 17 i den andra.

*Resultat av meta-analys av grupper exponerade kontra oexponerade*
Den sammanvägda risken, från de 22 studier som inkluderades i meta-analysen, för Raynauds fenomen var 4,56 (95% KI 3,00–6,95). Figur 10 gestaltar i en ”forest plot” resultaten av vår metaanalys på studier när vi jämför risken för Raynauds fenomen mellan grupper exponerade för HAV kontra oexponerade referensgrupper (figur10). Analysen har stratifierats mellan studier med lågrisk för bias (studier med högst kvalitetspoäng enligt tabell 5) kontra de med oklar risk för bias (studier med lägst kvalitetspoäng). Inom de två riskgrupperna (n=12 respektive n=10) har studierna rankats i fallande ordning från de med lägst risk för bias till de med högst risk för bias, utifrån deras totala kvalitetspoäng. Vidare framgår den relativa betydelsen varje studie har i meta-analysen.

Stratifieras metaanalysen mellan studier med låg risk för bias (studier med högst kvalitetspoäng enligt tabell 5) kontra de med otydlig/hög risk för bias (studier med lägst kvalitetspoäng) framgår stora skillnader. Gruppen av studier med låg risk för bias (n=12) redovisade en sammanvägd oddskvot på 6,85 (95% KI 4,17 – 11,25) medan guppen av studier med oklar risk (n=10) för bias hade en sammanvägd oddskvot på 2,76 (95% KI 1,69 – 4,50). Heterogeniteten var jämförbar mellan båda grupperna av studier [75% ($p < 0,01$) vs. 81% ($p< 0,01$)]. Den sammanvägda risken vid stratifierad analys är 4,32 (95% KI 3,05 – 6,12).

Stratifieras studierna utifrån publiceringsår (före respektive efter 1997; n=13 vs. n=9) noteras att sammanvägningen för äldre studier ger en högre oddskvot jämfört med nyare studier [6,03 ($p < 0,01$) vs. 3,13 ($p < 0,01$)], medan heterogeniteten är jämförbar [88 % ($p < 0,01$) vs. 85 % ($p < 0,01$)].

*Resultat av meta-analys av lågexponerad" kontra "högexponerad"*
Figur 11 beskriver resultaten av metaanalysen för de 17 studier som jämför risken för Raynauds fenomen mellan grupper exponerade för olika nivåer av HAV. Analysen har delats mellan de fyra olika kategorierna av dosmått. För respektive dosmått har studierna rangordnats i fallande ordning utifrån deras totala poäng (tabell 5). I vissa av studierna har dessutom flera exponeringsmått ingått, varför en studie kan återkomma på flera ställen.

För de olika dosmåtten varierade oddskvoten från 2,64 ($p < 0,01$) till 5,08 ($p < 0,01$). Heterogeniteten för de olika dosmåtten var 75% ($p < 0,01$), 81% ($p < 0,01$), 98% ($p < 0,01$) respektive 75% ($p < 0,01$).

| Stratifierad för: Risk för bias | Studie namn | Odds -kvot | 95%KI för oddskvot Lägre gräns | Övre gräns | Oddskvot och 95% konfidensintervall | Relativ betydelse i analysen |
|---|---|---|---|---|---|---|
| Låg | 78 Bovenzi, 2008*# | 45,04 | 5,90 | 343,67 | | 4,28 |
| Låg | 80 Bovenzi, 2010* | 3,39 | 1,55 | 7,44 | | 10,98 |
| Låg | 81 Bovenzi, 1995 | 11,80 | 4,47 | 31,12 | | 9,60 |
| Låg | 82 Bovenzi, 2010* | 3,54 | 1,74 | 7,21 | | 11,56 |
| Låg | 30 Bovenzi, 1998* | 18,63 | 7,58 | 45,83 | | 10,11 |
| Låg | 86 Bovenzi, 1988* | 6,06 | 2,17 | 16,96 | | 9,17 |
| Låg | 88 Bovenzi, 1994 | 9,33 | 4,90 | 17,76 | | 12,07 |
| Låg | 89 Nilsson, 1989 | 13,90 | 5,09 | 37,94 | | 9,35 |
| Låg | 90 Futatsuka, 2005*# | 0,39 | 0,02 | 6,43 | | 2,59 |
| Låg | 91 Jang, 2002*# | 15,25 | 2,07 | 112,08 | | 4,39 |
| Låg | 24 Bovenzi, 2005* | 0,48 | 0,14 | 1,65 | | 7,82 |
| Låg | 93 Letz, 1992* | 17,29 | 5,23 | 57,11 | | 8,07 |
| | ***Sammanvägt Låg risk*** | 6,85 | 4,17 | 11,25 | | |
| Oklar | 94 Chatterjee, 1978*# | 41,00 | 5,15 | 326,18 | | 4,04 |
| Oklar | 95 Brubaker, 1985* | 21,94 | 4,88 | 98,63 | | 6,16 |
| Oklar | 96 Burdorf, 1991* | 2,75 | 1,11 | 6,82 | | 9,76 |
| Oklar | 97 Cherniack, 2004* | 0,81 | 0,39 | 1,68 | | 11,07 |
| Oklar | 32 Anttonen, 1994* | 3,00 | 1,71 | 5,28 | | 12,32 |
| Oklar | 98 Virokannas, 1995* | 6,99 | 1,62 | 30,08 | | 6,37 |
| Oklar | 99 Bovenzi, 1980* | 6,40 | 2,20 | 18,56 | | 8,68 |
| Oklar | 28 Burstrom, 2010 | 2,02 | 1,66 | 2,46 | | 14,39 |
| Oklar | 26 Mirbod, 1994* | 1,76 | 1,21 | 2,55 | | 13,59 |
| Oklar | 100 Tominaga, 1994* | 1,01 | 0,70 | 1,46 | | 13,62 |
| | ***Sammanvägt Oklar risk*** | 2,76 | 1,69 | 4,50 | | |
| | ***Sammanvägt*** | 4,32 | 3,05 | 6,12 | | |

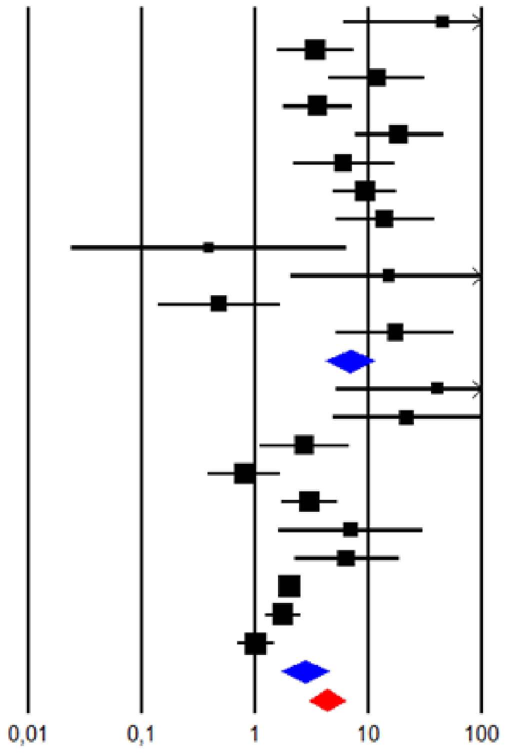


**Figur 10:** Statistik och ”forest plot” med sammanvägning från ”random – effekt” metaanalys av förekomsten av Raynauds fenomen mellan grupperna som utsätts för HAV respektive icke-exponerade referensgrupper. Storleken på fyrkanterna för de individuella studierna är proportionell mot den studiens betydelse i analysen. De blå diamanterna (romboider) visar den sammanvägda effekten för undergrupper av risk för bias och den röda diamanten anger den sammanvägda risken för alla studier. Studierna har sorterats i ordning från högsta till lägsta kvalitetspoäng enligt tabell 5. Asterisk (*) indikerar att studien presenterat data som gjort det möjligt att beräkna ojusterad oddskvot och # indikerar att studien noll-cell justerats.

| Stratifierad för: Dosmått | Studie namn | Odds-kvot | 95%KI för oddskvot Lägre gräns | Övre gräns | Relativ betydelse i analysen |
|---|---|---|---|---|---|
| Dos 1 | 80a Bovenzi, 2010 | 1,57 | 1,02 | 2,42 | 29,32 |
| Dos 1 | 95 Brubaker, 1985* | 9,09 | 3,41 | 24,23 | 18,07 |
| Dos 1 | 34 Walker, 1985* | 1,76 | 1,02 | 3,06 | 26,81 |
| Dos 1 | 92 Yamada, 1995* | 3,03 | 1,67 | 5,51 | 25,79 |
| Dos 1 | Sammanvägt Dos 1 | 2,64 | 1,44 | 4,82 | |
| Dos 2 | 80b Bovenzi, 2010 | 1,64 | 1,19 | 2,26 | 21,17 |
| Dos 2 | 97a Cherniack, 2004* | 9,45 | 3,43 | 26,03 | 16,26 |
| Dos 2 | 97b Cherniack, 2004* | 4,84 | 1,60 | 14,64 | 15,49 |
| Dos 2 | 93 Letz, 1992* | 8,98 | 3,75 | 21,47 | 17,43 |
| Dos 2 | 83 Mirbod, 1992* | 8,09 | 2,36 | 27,82 | 14,46 |
| Dos 2 | 33a Palmer, 1998 | 3,50 | 1,12 | 10,97 | 15,20 |
| Dos 2 | Sammanvägt Dos 2 | 4,90 | 2,19 | 10,96 | |
| Dos 3 | 30 Bovenzi, 1998 | 15,50 | 3,72 | 64,51 | 13,20 |
| Dos 3 | 82 Bovenzi, 2010* | 4,31 | 1,75 | 10,62 | 18,56 |
| Dos 3 | 24 Bovenzi, 2005 | 1,60 | 0,60 | 4,25 | 17,71 |
| Dos 3 | 84 Bovenzi, 2011 | 1,19 | 1,03 | 1,37 | 25,19 |
| Dos 3 | 28 Burstrom, 2010 | 3,53 | 3,22 | 3,87 | 25,33 |
| Dos 3 | Sammanvägt Dos 3 | 2,94 | 1,39 | 6,21 | |
| Dos 4 | 80c Bovenzi, 2010 | 2,19 | 1,63 | 2,94 | 25,54 |
| Dos 4 | 33b Palmer, 1998 | 2,20 | 1,13 | 4,30 | 22,09 |
| Dos 4 | 85 Hagberg, 2008 | 4,28 | 0,68 | 26,99 | 10,52 |
| Dos 4 | 88 Bovenzi, 1994 | 10,11 | 3,32 | 30,78 | 17,05 |
| Dos 4 | 81 Bovenzi, 1995 | 8,45 | 1,49 | 47,84 | 11,29 |
| Dos 4 | 91 Jang, 2002* | 30,45 | 7,04 | 131,75 | 13,51 |
| Dos 4 | Sammanvägt Dos 4 | 5,08 | 2,35 | 10,95 | |

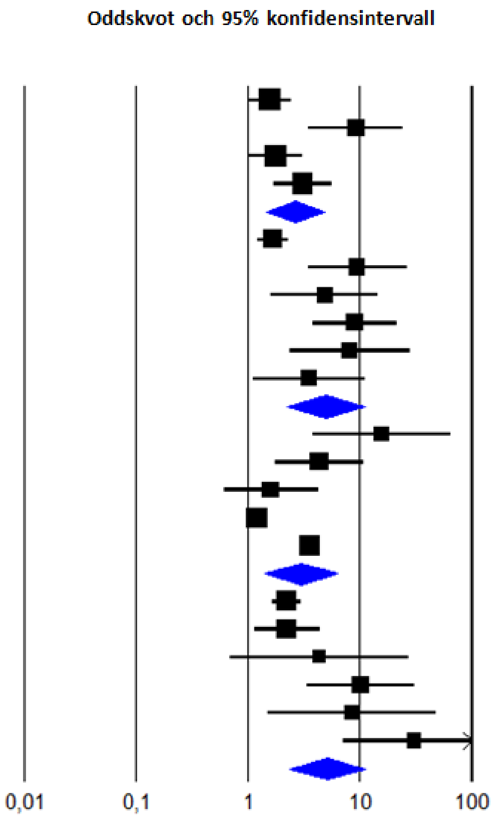


**Figur 11:** Statistik och Forest plot med sammanvägning för respektive dos för studier på Raynauds fenomen sorterat efter olika dosmått. Storleken på fyrkanten för de individuella studierna är proportionell mot den studiens betydelse i analysen. De blå diamanterna (romboider) visar den sammanvägda effekten för undergrupper av dosmått; Dos 1 = Antal exponeringsår (år); Dos 2 = Antal exponeringstimmar (tim); Dos 3 = Daglig vibrationsexponering, A(8) ($m/s^2$); Dos 4 = Kumulerad vibrationsexponering ($mh/s^2$, $m^2h^3/s^4$). Studierna har sorterats i ordning från högsta till lägsta kvalitetspoäng enligt tabell 5. Asterisk (*) indikerar att studien presenterat data som gjort det möjligt att beräkna ojusterad oddskvot.

*Resultat av meta-regressionsanalys*
Figur 12 visar resultatet av meta-regressionsanalys av sambandet mellan den dagliga accelerationen, A(8) och naturliga logaritmen för oddskvoten av Raynauds fenomen hos vibrationsexponerade. Resultatet bygger på uppgifter från 14 av de 22 studierna i figur 10. I dessa 14 studier finns uppgifter som beskriver den daglig ekvivalent vibration A(8) eller med uppgifter som möjliggjort beräkningar av A(8).

Analysen visar att sambandet är tillförlitligt ($p<0{,}001$) och koefficienten för A(8) är 0,09, vilket betyder att för varje ökning med 1 $m/s^2$ ökar oddskvoten för Raynauds fenomen med 9%. Resultaten visar också att det finns en signifikant ($p<0{,}001$) variation mellan studierna, vilket indikerar att även om A(8) är densamma i två studier varierar prevalensen för Raynauds fenomen. Modellen förklarar 16% av variationen mellan studierna.

Trattdiagram (”funnel plot”) på de i metaanalysen ingående studierna presenteras i figur 13. Resultat där studierna fördelas symmetriskt kring den beräknade effekten tyder på liten effekt för publikationsbias.

## Summering

Kunskapsöversikten identifierade 41 studier som uppfyllde inklusionskriteriena för att belysa sambandet mellan Raynauds fenomen och vibrationsexponering. Av dessa var 11 kohortstudier, 2 fall-kontrollstudier och 28 tvärsnittsstudier. Studier med låg risk för bias (hög kvalité) fanns inom alla tre undersökningsdesignerna.

Prevalensen av Raynauds fenomen varierade från 0% till 53% bland de vibrationsexponerade. De studier där ingen av de vibrationsexponerade haft Raynauds fenomen har uteslutande utförts i varmt klimat. Studierna visade på ett dos-respons samband, där högre prevalenser för Raynauds fenomen var relaterade till högre vibrations-exponeringsnivåer.

För de 22 studier som medgav statistisk sammanvägning visade 18 studier på en signifikant överrisk. Den sammanvägda risken för vibrationsexponerade var 4,56 (95% KI 3,00 – 6,95) jämfört med oexponerade. För studier med låg risk för bias var motsvarande sammanvägd risk 6,83 (95% KI 3,71 – 12,56). Exponerings-bestämning med antal exponeringstimmar/dag resulterade i en sammanvägd oddskvot på 4,90 (95% KI 2,19 – 10,96), medan en något högre sammanvägd oddskvot erhölls när vibrationsexponeringen togs med men med större variation. Meta- regressionsanalys indikerar ett dos-responssamband för Raynauds fenomen i relation till vibrationsexponeringsnivå. Enligt denna analys medför en exponeringsökning på 1 $m/s^2$ att relativa risken för vita fingrar ökar med 9%

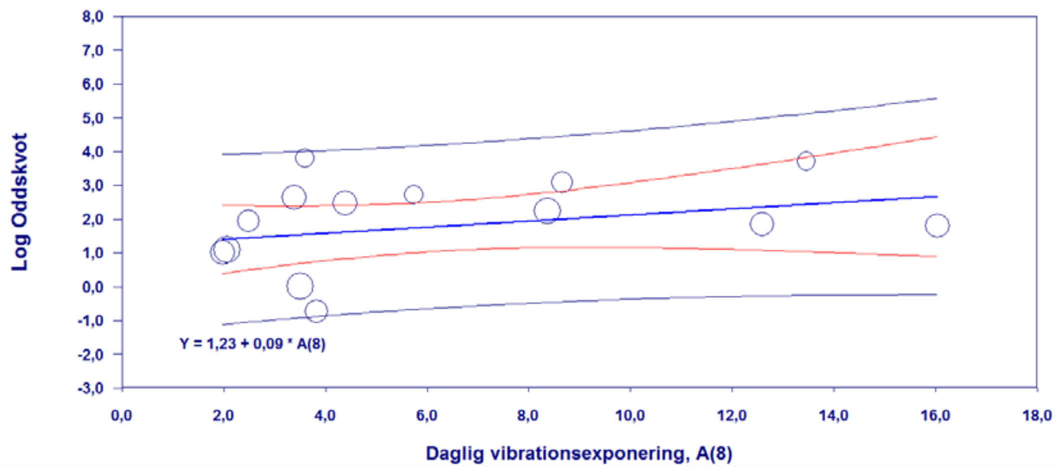


**Figur 12:** Metaregressionsanalys av sambandet mellan logaritmen för oddskvoten av Raynauds fenomen hos vibrationsexponerade och vibrationsexponeringen uttryckt som A(8) (random effekt; n=14). I figuren framgår också regressionslinjens 95% konfidens-intervall. Cirklarnas storlek beskriver respektive studies vikt vid beräkningar av regressionen.

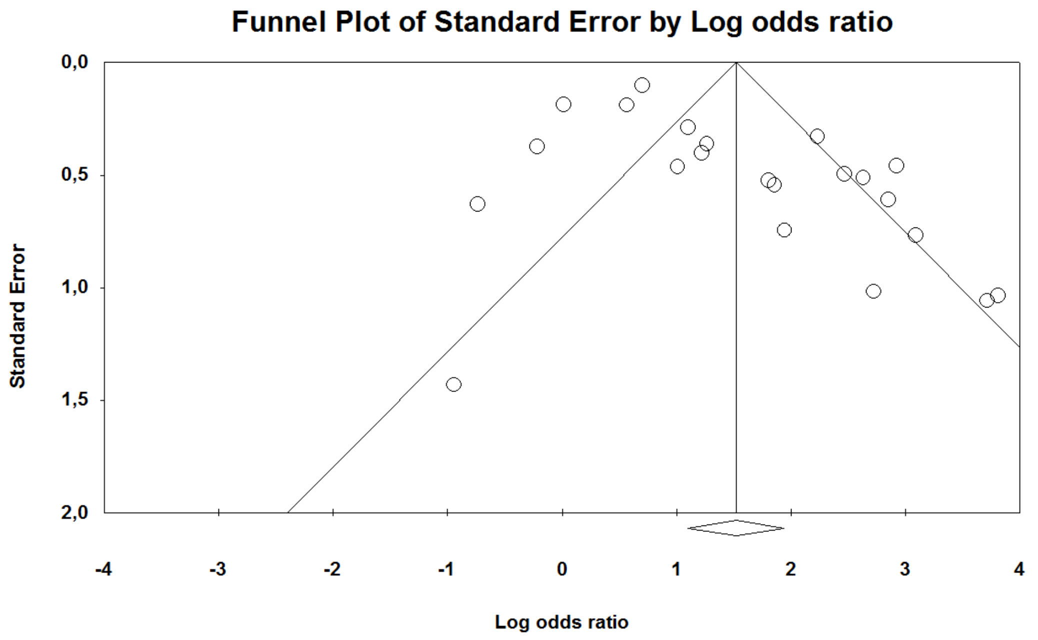


**Figur 13:** Trattdiagram (”funnel plot”) med pseudo 95% konfidensintervall för publikationsbias i studier av samband mellan förekomsten av Raynauds fenomen bland grupper som utsätts för HAV respektive icke-exponerade referensgrupper (Beggs test p = 0.14; Eggers test p<0.01; Trim och fyll metod räknade tre saknade studier till vänster om medelvärdet (random-effekt-modellen).

## Neurosensorisk skada

**Definition**

Arbetare som använde luftdrivna bergborrar rapporterade redan vid 1900-talets början besvär i form av ”stickningar” och ”känselnedsättning” (18) förutom av attacker av ”vita fingrar” (se föregående avsnitt om Raynauds fenomen). Alice Hamilton (17) beskriver 1918 att hon informerats att kalkstensbrytare som använde luftdrivna bergborrar riskerade att även drabbas av ”neurasteni, förlamning och sinnessjukdom”, förutom av tuberkulos och ”döda” fingrar. Skadade arbetare beskrev själva symptom i form av en ökad tendens att ”händerna somnar”, särskilt om händerna ”håller i en tidning eller i en bok”. De klagade även över ”försämrad känsel och en försämrad finmotorik, som medförde svårigheter att knäppa knappar, knyta skosnören samt att skilja olika mynt från varandra med enbart känsel”. Allmänna läkarundersökningar bekräftade fynd av nedsatt känsel i händerna hos ett antal av dessa kalkstensarbetare (17).

Dessa tidigt rapporterade manifestationer innefattar bortfall av känsel, retningssymptom och en försämring vid vissa aktiviteter, allt förenligt med perifer neurosensorisk skada innefattande även misstanke på karpaltunnelsyndrom.

Neurosensorisk skada innebär störning i det perifera nervsystemets sensoriska enheters (ändorgan med afferent nerv- och cellkropp) funktion eller på störningar i det centrala nervsystemet.

Allmän neurosensorisk skada manifesterar sig som känselnedsättning (negativ manifestation), tillkomst av retningsmanifestationer såsom symptom på ”smärta”, ”pirrningar”, ”sockerdrickskänsla”, eller” domningsförnimmelser” (positiva manifestationer) eller som ökad känslighet vid provokation (provocerbara manifestationer).

Negativa manifestationer kan diagnostiseras med elektrodiagnostik (nervledningsundersökning av motoriska-, A-β och A-δ fibrer samt med elektromyogram) samt med kvantitativ sensorisk testning (QST) av berörings-sinne (känselkropp/ändorgan för vibration och A-β fibrer), temperatursinne för kyla (ändorgan och A- δ fibrer), för värme (ändorgan och C-fibrer) samt för smärta (C-fibrer). Kraftnedsättning representerar nedsättning i motoriska enhetens (muskelfibrer och β-fibrer) funktion. Neuromotorisk störning kan även innefatta rörelsestörningar i form av tremor.

Positiva manifestationer, det vill säga symptom på stickningar, sockerdrickskänsla och smärta, kan inte verifieras med elektrodiagnostiska metoder eller med laboratorieprov.

Provocerbara manifestioner kan framkallas med kliniska test som Tinells tecken, Phalens test och Roos test (abduction external rotation test).

En provokation med lokalt tryck eller en mekanisk retning medför akuta nervsymptom som vanligen är övergående. Tolkning av manifestationer från nervsystemet kräver att akuta symptom särskiljs från tecken på bestående skada.

Funktionstest används ibland för att dokumentera den integrerade funktionen av sensorisk och motorisk funktion. Purdue pegboard-testet är ett exempel på ett

sådant diagnostiskt funktionsprov och har tillsammans med andra undersökningar redovisats i enstaka av våra ingående studier.

### Diagnostiska överväganden och risk för bias

Diagnostisk ambition och krav på diagnostisk upplösning betingas av vilka åtgärder en specifik diagnos medför. Hand-arm vibrationsskadesyndromets neurosensoriska skador saknar, förutom för karpaltunnelsyndrom, verksam medicinsk behandling och saknar därför medicinska behandlingskonsekvenser och har följaktligen haft låga krav på diagnostisk precision. Primärprevention med reducerad exponering utgör den enda och viktigaste åtgärden vid neurosensorisk skada till följd av arbete med vibrerande verktyg.

Vilka diagnoskriterier för nervskada som tillämpas varierar med frågeställning och varierar därmed mellan olika studier. Epidemiologiska studier, fall-serier av patienter, arbetsskadefrågeställningar tillämpar alla olika kriterier för skada och grad av nervskada. Diagnostiskt särskiljs akuta manifestationer (*trigger kriterium*) från bestående skada. Sensoriska skador särskiljs på modalitet (beröring, temperatur) och om symptomen är bortfallssymptom (känsel- eller kraftnedsättning) eller symptom på retning (nytillkomna symptom) (*kliniskt kriterium*). Nervskada kan yttra sig både som ett känselbortfall (exempelvis ”känner inte värme”) och som en ökad känslighet (”tål inte kyla”) eller som att en normal stimulering medför smärta (”kyla gör ont”). För att vibrationsrelaterad neurosensorisk skada skall kunna övervägas, skall tillräcklig hand-arm överförd vibrationsexponering (*exponeringskriterium*) ha föregått sjukdomsdebuten (*tidssambandskriterium*), samtidigt som det inte framkommit någon annan känd faktor som kan orsaka neurosensorisk skada (*exklusionskriterium*).

### Gradering av skadans allvarlighetsgrad

Synen på vibrationsrelaterad sjukdom uppvisar historiskt och globalt två huvudinriktningar, den *östliga* där alla olika manifestationer tolkas som del i en ”vibrationssjukdom” samt den *västliga* där olika yttringar hänförs till ett hand-arm vibrations syndrom. I västvärldens tidiga begreppsbildning tolkades symptom på stickningar och domningar som utslag av cirkulationspåverkan och sågs följaktligen som tidiga tecken på Raynauds fenomen snarare än som en specifik nervskada, allt förenligt med begreppet hand-arm vibrationsskade-syndromet.

Bland det flertal av manifestationer som ingår i den ryska stadieindelningen (66) innefattas perifer nervskada av ”vegetativt polyneurit syndrom” och neurit, medan den västerländska stadieindelningen tidigt begränsades till stickningar (positiva manifestationer), känselnedsättning (negativa manifestationer) och vita fingrar, men allvarlighetsgraden klassificerades utifrån symptomens interaktion med arbete och handikapp.

Tidiga kliniska tecken på HAVS-skada, i form av Raynuds fenomen, betraktades länge som harmlösa. Detta då tecknen knappt medförde några besvär, symptomen kunde förbli ouppmärksammade av den drabbade och behövde ej

interferera med arbete eller fritidsaktiviteter (67). Lindriga ”stickningar” och / eller ”nedsatt känsel” kunde följa i ett senare skede. Med denna föreställning om sjukdomsförloppet, tillsammans med erfarenheter från undersökningar 1969 av skogsarbetare, kom graderingen av Raynauds fenomen att utformas i enlighet med hur besvären yttrar sig och i vilken mån det medför handikapp eller interfererar med det sociala livet och arbete (67, 68) (tabell 3).

Taylor-Pelmear-skalan reviderades så att $0_{TN}$ kom att markera samtidig förekomst av både stickningar (”tingling”) och känselnedsättning (”numbness”).

1986 bröts den gemensamma stadieindelningen upp i en skala för kärl (69) och en skala för neurosensorisk stadieindelning (70). I de studier som omfattas av kunskapsöversikten klassificeras neurosensoriska symptoms allvarlighetsgrad enligt den tidiga Taylor-Pelmear-klassificeringen (tabell 3) i 5 studier och enligt Stockholm Workshop-skalan (tabell 6) i 11 studier.

**Tabell 6:** Gradering av symptomen vid neurosensorisk skada hos vibrationsexponerade (Stockholm Workshop-skalan för sensorisk funktionsnedsättning). Översättning från originalet (70).

| Stadium[a] | Symptom |
|---|---|
| 0SN | Exponerad för vibrationer men inga symptom |
| 1SN | Intermittent domning, med eller utan stickningar (tingling) |
| 2SN | Intermittent eller varaktig domning; nedsatt sensorisk perception |
| 3SN | Intermittent eller varaktig domning; nedsatt taktil diskriminationsförmåga och/eller nedsatt finmotorik |

[a]Graderingen skall anges separat för båda händerna.

## Möjlig bias till följd av interaktion från andra exponerings- och individfaktorer

Ett flertal medicinska förhållanden och exponeringar (bilaga 4) kan relateras till neurosensoriska besvär. Rhizopati (rotpåverkan), eller mononeuropati (påverkan på en enstaka perifer nerv) i övre extremitet i form av tryck (entrapment av ex. n. radialis vid överarm eller armbåge, n. ulnaris och n. medianus vid armbåge eller handled) kan liksom en polyneuropati ge symptom som vid neurosensoriska skador orsakade av vibrationer. Polyneuropati medför oavsett genes vanligen en likartad symptombild där manifestationerna uppvisar ett längstberoende. I varierande grad förekommer motoriska och sensoriska bortfallssymptom och retningssymptom, liksom autonom dysfunktion. En låggradig bakomliggande polyneuropati behöver ej vara symptomgivande. Såväl polyneuropati som monopati förekommer. De vanligaste bakomliggande orsakerna till perifer polyneuropati hänförs till diabetes, ämnesomsättningsstörning, behandling för malign cancersjukdom och överkonsumtion av alkohol. Polyneuropati av konkurrerande genes kan dessutom hänföras till intoxikationer, systemsjukdomar,

infektioner, bristtillstånd, immunopatier, och ärftliga tillstånd (detaljerad beskrivning framgår av bilaga 4).

Ett flertal individrelaterade sjukdomar, omständigheter och exponeringar har samband med neurosensorisk skada. I vilken mån ingående studier angett att de tagit hänsyn till andra möjliga orsaker till nervpåverkan utifrån läkarundersökning, laboratoriescreening eller nervledningsundersökningar har tagits med i vår bedömning av möjlig risk för diagnostisk bias.

**Resultat av den systematiska litteratursökningen för neurosensorisk skada**

Resultatet av den systematiska litteraturgenomgången för de studier som behandlat neurosensorisk skada framgår av tabell 7. Av tabellen framgår dels vår skattning av risk för bias (kvalitetspoäng) avseende diagnosens tillförlitlighet och den totala summan av kvalitetspoäng. Totalsumman är summan av kvalitetspoängen för diagnos adderat till kvalitetspoängen för ”studie-metod” och ”exponering”.

För utfallet neurosensorisk skada innefattar kunskapsöversikten 33 studier varav 7 studier är unika för neurosensorisk skada. Sju studier ingår för karpaltunnelsyndrom varav 2 är unika för KTS. Fem studier tar upp både neurosensorisk skada och karpaltunnelsyndrom (figur 4). För enbart utfallet neurosensorisk skada ingår studier publicerade från 1978 till 2013.

Sammanställningen (tabell 7) visar att de 33 studierna behandlat nervpåverkan med en variation av diagnossumma mellan 1 och 11 för diagnostisk tillförlitlighet (kvalitetskriterier) gällande neurosensorisk skada.

*Prevalens av neurosensorisk skada i relation till publiceringsår*
I de 33 studierna finns unika uppgifter redovisade i 22 studier om prevalens av neurosensorisk skada bland vibrationsexponerade. I en av studierna ingår uppgifter om prevalens bland flera grupper av exponerade. De sammanställda 26 uppgifter presenteras i figur 14 sorterade efter publiceringsår.

Figur 14 visar att förekomsten av neurosensorisk skada har varierat från 7% till 79% i de olika studierna med ett genomsnitt på 39%.

*Prevalens av neurosensorisk skada i relation till vibrationsexponering A(8)*
I 19 av studierna finns uppgifter om vibrationsexponering beskriven som daglig ekvivalent vibration A(8) eller så finns uppgifter som möjliggjort beräkningar av A(8). I vissa av studierna har dessutom flera grupper av exponerade ingått vilket ger ett totalt antal uppgifter på 23 där både prevalens och exponering rapporterats figur 15 visar sambandet mellan A(8) och prevalens av neurosensorisk skada.

Figur 15 visar att prevalensen varierat från 6% till knappt 80% (medel 39%; median 39%) medan A(8) varierat från 0,6 m/s$^2$ till ca 30 m/s$^2$ (medel; 5,8 m/s$^2$; median 2,7 m/s$^2$). Linjär regression visar att för varje fördubbling av A(8) ökar prevalensen av neurosensorisk skada med ca 7 procentenheter. I 17 studier har även medelvärdet av den totala exponeringstiden för vibrationer i gruppen redovisats och den varierar mellan 4 år och knappt 23 år (medel 13 år; median 12 år).

**Tabell 7:** Ingående studier på neurosensorisk skada samt deras bedömda risk för bias (kvalitetspoäng) avseende falldefinitionen för denna skada (diagnos summa) samt totalsumma när kvalitetspoängen för bedömningen diagnos neurosensorisk skada, ”studie metod” och ”exponering” adderats (Total summa). Studierna presenteras i fallande ordning utifrån den totala poängsumman. Högre poäng indikerar högre ”kvalitet”, vilket indikerar mindre möjlig risk för bias. Vidare anges studiedesign för respektive studie. n=

| Studie | Referens | Design | Diagnos summa | Total summa |
|---|---|---|---|---|
| Bovenzi, 2011* | (101) | Kohort | 11 | 25 |
| Bovenzi, 2000 | (79) | Fall-kontroll | 9 | 24 |
| Mirbod, 1992* | (83) | Fall-kontroll | 11 | 23 |
| Mirbod, 1999 | (49) | Kohort | 9 | 23 |
| Nilsson, 2008 | (29) | Tvärsnitt | 9 | 20 |
| Barregard, 2003 | (43) | Fall-kontroll | 8 | 20 |
| Nilsson, 2001 | (53) | Tvärsnitt | 8 | 20 |
| Ho, 1986 | (51) | Tvärsnitt | 11 | 19 |
| Su, 2013* | (31) | Tvärsnitt | 9 | 19 |
| Bovenzi, 1994* | (88) | Tvärsnitt | 8 | 19 |
| Futatsuka, 2005* | (90) | Tvärsnitt | 8 | 18 |
| Edlund, 2013* | (102) | Kohort | 2 | 18 |
| Bovenzi, 1988* | (86) | Tvärsnitt | 8 | 17 |
| Bovenzi, 1985* | (87) | Tvärsnitt | 7 | 17 |
| Palmer, 1998* | (33) | Tvärsnitt | 6 | 17 |
| Yamada, 1995 | (92) | Tvärsnitt | 10 | 16 |
| Lundstrom, 1999 | (52) | Tvärsnitt | 6 | 16 |
| Koskimies, 1992 | (48) | Tvärsnitt | 5 | 16 |
| Jang, 2002* | (91) | Tvärsnitt | 4 | 16 |
| Matsumoto, 1995* | (25) | Tvärsnitt | 9 | 15 |
| Gerhardsson, 2005 | (46) | Tvärsnitt | 6 | 15 |
| Letz, 1992* | (93) | Tvärsnitt | 4 | 15 |
| Chatterjee, 1978* | (94) | Tvärsnitt | 6 | 14 |
| Malchaire, 2001* | (103) | Tvärsnitt | 4 | 14 |
| Burdorf, 1991* | (96) | Tvärsnitt | 3 | 14 |
| Cherniack, 2004* | (97) | Tvärsnitt | 3 | 14 |
| Brubaker, 1985* | (95) | Tvärsnitt | 4 | 13 |
| Anttonen, 1994* | (32) | Tvärsnitt | 1 | 12 |
| Virokannas, 1995* | (98) | Tvärsnitt | 1 | 12 |
| Bovenzi, 2005* | (24) | Tvärsnitt | 2 | 11 |
| Musson, 1989 | (50) | Tvärsnitt | 1 | 10 |
| Bovenzi, 1980* | (99) | Tvärsnitt | 1 | 9 |
| Mirbod, 1994* | (26) | Tvärsnitt | 1 | 7 |

*Studien är med i meta-analysen

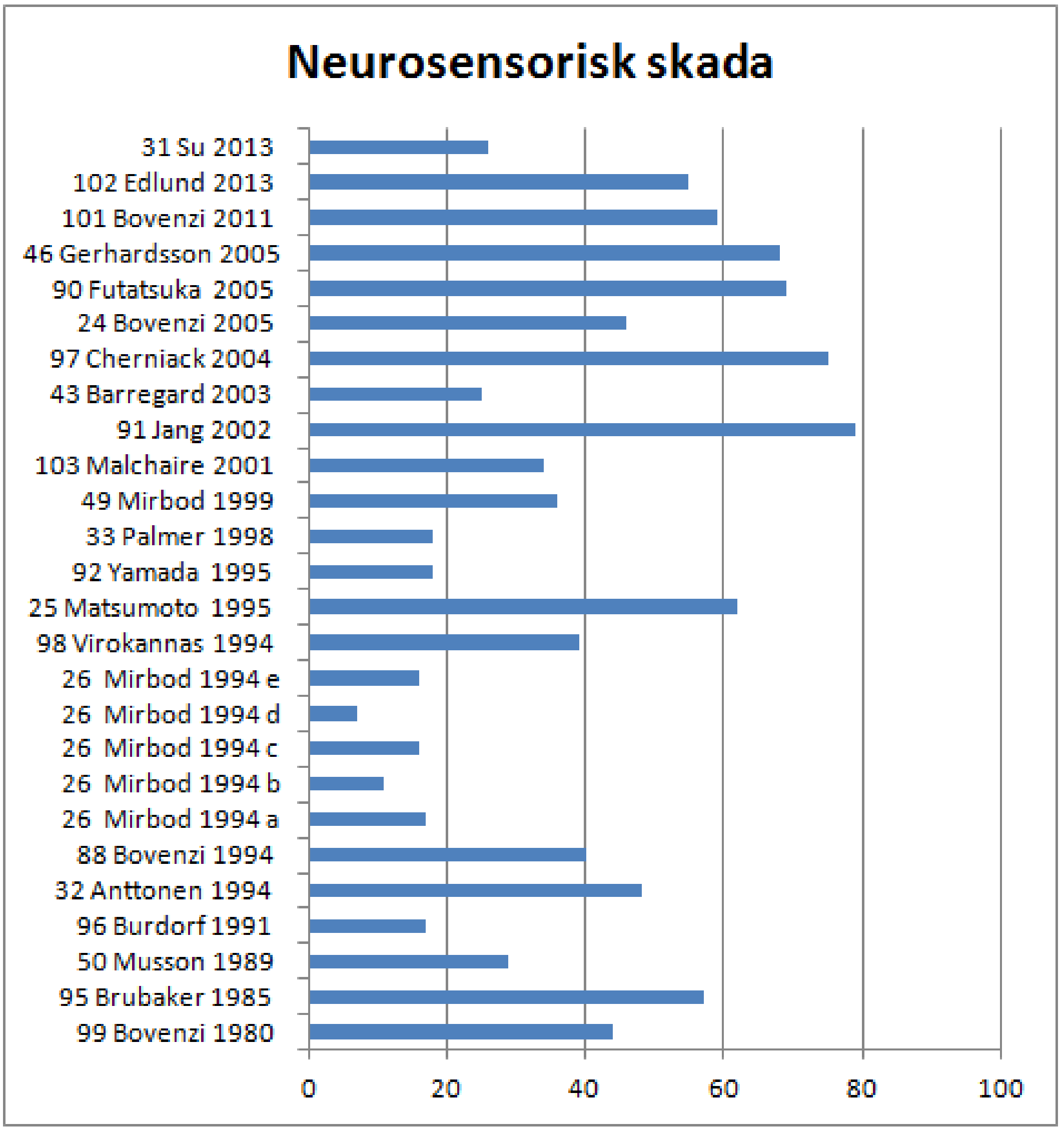


**Figur 14:** Studier med uppgift om prevalens för neurosensorisk skada i den undersökta studiepopulationen sorterade efter publiceringsår. n=26

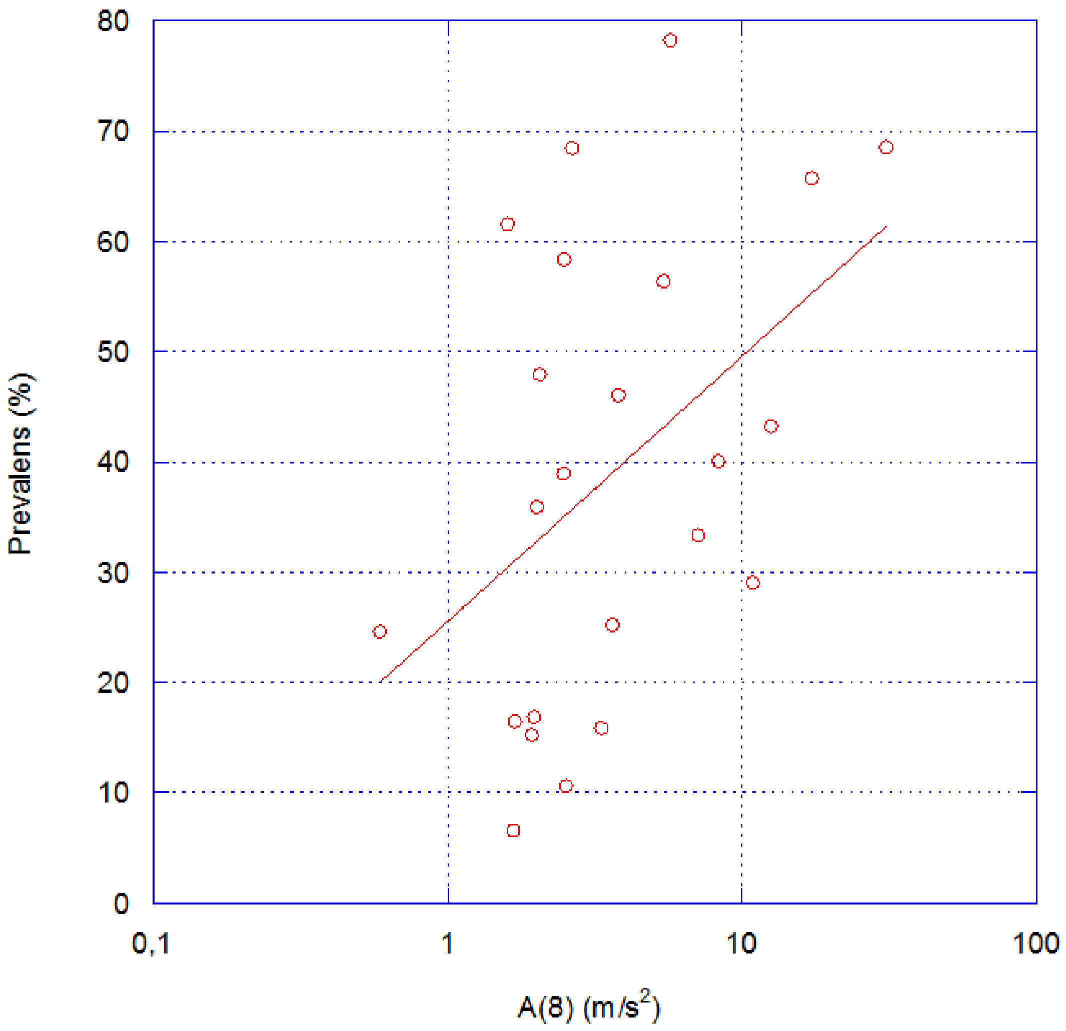


**Figur 15:** Prevalens (%) av neurosensorisk skada i relation till vibrationsnivå A(8) (n=23) samt den linjära regressionslinjen (Ekvation: Prevalens (%)=25,6+24,1*$\log_{10}$(A(8)); r=0,45). n=23

### Resultat av meta-analys från studier på neurosensorisk skada

Meta-analysen har delats upp dels mellan exponerade och icke-exponerade dels mellan "lågexponerad" och "högexponerad". I meta-analysen har totalt ingått 22 studier, 18 i den första analysen och 7 i den andra.

*Resultat av meta-analys av grupper exponerade kontra oexponerade*

Den sammanvägda risken, från de 18 studier som inkluderades i meta-analysen, för neurosensorisk skada var 4,58 (95% KI 3,28–6,38) med en heterogenitet av 76 % ($p < 0,01$). Figur 16 gestaltar i en ”forest plot” resultaten av vår metaanalys på studier när vi jämför risken för neurosensorisk skada mellan grupper exponerade för HAV kontra oexponerade referensgrupper (figur16). Analysen har

stratifierats mellan studier med lågrisk för bias (studier med högst kvalitetspoäng enligt tabell 7) kontra de med oklar risk för bias (studier med lägst kvalitets-poäng). Inom de två riskgrupperna (n=8 respektive n=10) har studierna rankats i fallande ordning från de med lägst risk för bias till de med högst risk för bias, utifrån deras totala kvalitetspoäng. Vidare framgår den relativa betydelsen varje studie har i meta-analysen.

Stratifieras meta-analysen mellan studier med låg risk för bias (studier med högst kvalitetspoäng enligt tabell 7) kontra de med otydlig/hög risk för bias (studier med lägst kvalitetspoäng) framgår vissa skillnader. Gruppen av studier med låg risk för bias (n=8) redovisade en sammanvägd oddskvot på 7,37 (95% KI 4,39 – 12,37) medan guppen av studier med oklar risk (n=10) för bias hade en sammanvägd oddskvot på 3,31 (95% KI 2,17 – 5,06). Heterogeniteten var densamma för båda grupperna av studier [74 % (p < 0,01) vs. 74 % (p< 0,01)]. Den sammanvägda risken vid stratifierad analys är 4,32 (95% KI 3,05 – 6,12).

Stratifieras studierna utifrån publiceringsår (före resp. efter 1997; n=12 respektive n=6) noteras att sammanvägningen inte medför någon större skillnad i risk estimat mellan tidiga respektive senare studier [5,08 (p < 0,01) vs. 4,46 (p < 0,01)] och heterogeniteten är snarlik [76 % (p < 0,01) vs. 78 % (p < 0,01)].

*Resultat av meta-analys av lågexponerad" kontra "högexponerad"*

Figur 17 beskriver resultaten av metaanalysen av de 7 studier som jämför risken för neurosensorisk skada mellan grupper exponerade för olika nivåer av HAV. Analysen har också delats mellan de två olika kategorierna av dosmått som presenterats. För respektive dosmått har studierna rankats i fallande ordning utifrån deras totala poäng. I två studier har dessutom flera exponeringsmått ingått varför studien kan återkomma på flera ställen.

För de två dosmåtten varierade oddskvoten från 2,67 (p < 0,01) till 4,77 (p < 0,01). Heterogeniteten för Dos 2 var 76% (p < 0,01) och för Dos 4, 15% (p=0,32).

*Resultat av meta-regressionsanalys*

I figur 18 presenteras resultatet av metaregressionsanalys av sambandet mellan A(8) och naturliga logaritmen för oddskvoten av neurosensorisk skada hos vibrationsexponerade. Resultatet bygger på uppgifter från 15 av de 18 artiklarna i figur 16. I dessa 15 artiklar finns uppgifter som beskriver den daglig ekvivalent vibration A(8) eller med uppgifter som möjliggjort beräkningar av A(8).

Analysen visar att sambandet är statistiskt signifikant (p=0,001) och koefficienten för A(8) är 0.08, vilket betyder att för varje ökning med 1 $m/s^2$ ökar oddskvoten med 8%. Resultaten visar också att det finns en signifikant (p<0,001) variation mellan studierna, vilket indikerar att även om A(8) är densamma i två studier varierar prevalensen för neurosensorisk skada. Modellen förklarar 57% av variationen mellan studierna.

| Stratifierad för: Risk för bias | Studie namn | Odds-kvot | 95%KI för oddskvot Lägre gräns | Övre gräns | Relativ betydelse i analysen |
|---|---|---|---|---|---|
| Låg | 101 Bovenzi, 2011* | 21,94 | 4,88 | 98,63 | 7,75 |
| Låg | 88 Bovenzi, 1994 | 3,49 | 2,36 | 5,16 | 19,83 |
| Låg | 90 Futatsuka, 2005* | 29,35 | 6,43 | 134,03 | 7,65 |
| Låg | 86 Bovenzi, 1988* | 2,49 | 1,16 | 5,36 | 15,01 |
| Låg | 87 Bovenzi, 1985* | 42,09 | 9,35 | 189,40 | 7,75 |
| Låg | 91 Jang, 2002* | 6,97 | 3,74 | 13,01 | 16,88 |
| Låg | 25 Matsumoto, 1995* | 4,80 | 1,45 | 15,94 | 10,16 |
| Låg | 93 Letz, 1992* | 9,51 | 4,41 | 20,54 | 14,96 |
| | ***Sammanvägt Låg risk*** | 7,37 | 4,39 | 12,37 | |
| Oklar | 94 Chatterjee, 1978* | 30,47 | 6,45 | 144,04 | 4,94 |
| Oklar | 103 Malchaire, 2001* | 1,98 | 0,77 | 5,12 | 8,49 |
| Oklar | 96 Burdorf, 1991* | 2,87 | 1,01 | 8,18 | 7,78 |
| Oklar | 97 Cherniack, 2004* | 2,62 | 1,34 | 5,15 | 10,79 |
| Oklar | 95 Brubaker, 1985* | 2,90 | 1,10 | 7,68 | 8,32 |
| Oklar | 32 Anttonen, 1994* | 2,09 | 1,52 | 2,86 | 13,73 |
| Oklar | 98 Virokannas, 1995* | 1,86 | 0,96 | 3,60 | 10,91 |
| Oklar | 24 Bovenzi, 2005 | 2,70 | 1,63 | 4,48 | 12,26 |
| Oklar | 99 Bovenzi, 1980* | 6,84 | 2,79 | 16,78 | 8,91 |
| Oklar | 26 Mirbod, 1994* | 5,43 | 4,03 | 7,31 | 13,86 |
| | ***Sammanvägt Oklar risk*** | 3,31 | 2,17 | 5,06 | |
| | ***Sammanvägt*** | 4,56 | 3,29 | 6,33 | |

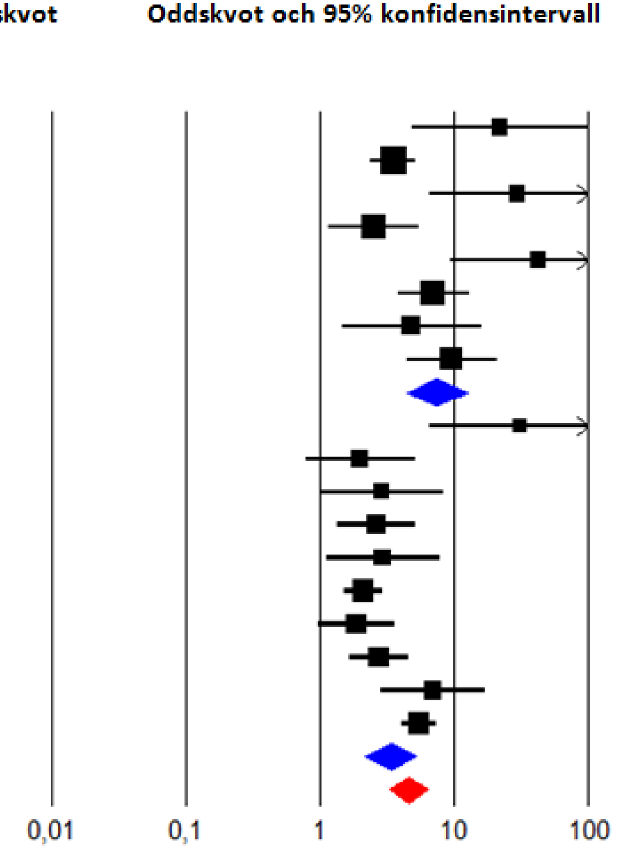


**Figur 16:** Statistik och ”forest plot” med sammanvägning från ”random – effekt” metaanalys av förekomsten av neurosensorisk skada mellan grupperna som utsätts för HAV respektive icke-exponerade referensgrupper. Storleken på fyrkanterna för de individuella studierna är proportionell mot den studiens betydelse i analysen. De blå diamanterna (romboider) visar den sammanvägda effekten för undergrupper av risk för bias och den röda diamanten anger den sammanvägda risken för alla studier. Studierna har sorterats i ordning från högsta till lägsta kvalitetspoäng enligt tabell 5. Asterisk (*) indikerar att studien presenterat data som gjort det möjligt att beräkna ojusterad oddskvot och # indikerar att studien noll-cell justerats.

| Stratifierad för: Dosmått | Studie namn | Odds-kvot | 95%KI för oddskvot Lägre gräns | Övre gräns | Oddskvot och 95% konfidensintervall | Relativ betydelse i analysen |
|---|---|---|---|---|---|---|
| Dos 2 | 97 Cherniack, 2004a* | 4,34 | 1,95 | 9,67 | | 20,62 |
| Dos 2 | 97 Cherniack, 2004b* | 4,00 | 1,52 | 10,53 | | 18,63 |
| Dos 2 | 93 Letz, 1992* | 17,18 | 6,62 | 44,61 | | 18,78 |
| Dos 2 | 83 Mirbod, 1992* | 5,58 | 2,06 | 15,12 | | 18,28 |
| Dos 2 | 33 Palmer, 1998a | 1,90 | 1,11 | 3,24 | | 23,68 |
| Dos 2 | ***Sammanvägt Dos 2*** | 4,77 | 2,25 | 10,13 | | |
| Dos 4 | 33 Palmer, 1998b | 2,20 | 1,18 | 4,12 | | 28,54 |
| Dos 4 | 102 Edlund, 2013 | 3,00 | 1,32 | 6,82 | | 18,27 |
| Dos 4 | 31 Su, 2013 | 3,34 | 1,26 | 8,88 | | 13,43 |
| Dos 4 | 88 Bovenzi, 1994 | 1,61 | 0,77 | 3,37 | | 21,81 |
| Dos 4 | 91 Jang, 2002* | 5,05 | 2,21 | 11,58 | | 17,96 |
| Dos 4 | ***Sammanvägt Dos 4*** | 2,67 | 1,83 | 3,90 | | |

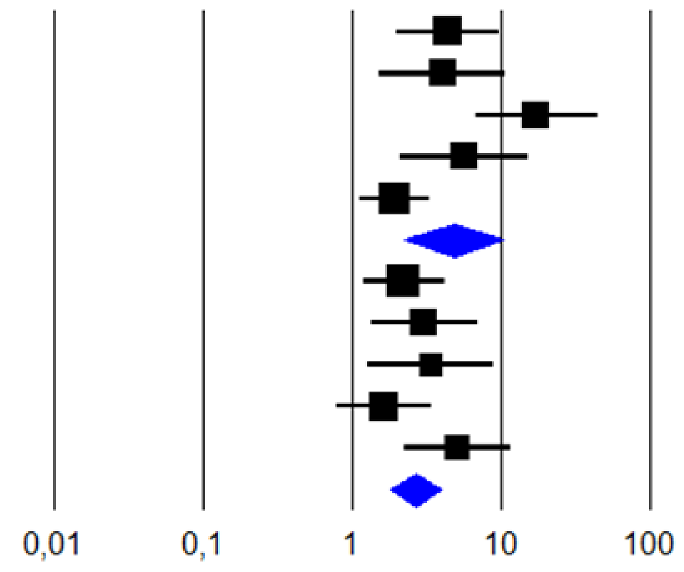


**Figur 17:** Statistik och Forest plot med sammanvägning för respektive dos för studier på neurosensorisk skada sorterat efter olika dosmått. Storleken på fyrkanten för de individuella studierna är proportionell mot den studiens betydelse i analysen. De blå diamanterna (romboider) visar den sammanvägda effekten för undergrupper av dosmått; Dos 2 = Antal exponeringstimmar (tim); Dos 4 = Kumulerad vibrationsexponering ($mh/s^2$, $m^2h^3/s^4$). Studierna har sorterats i ordning från högsta till lägsta poäng kvalitetspoäng enligt tabell 7. Asterisk indikerar att studien presenterat data som gjort det möjligt att beräkna ojusterad oddskvot.

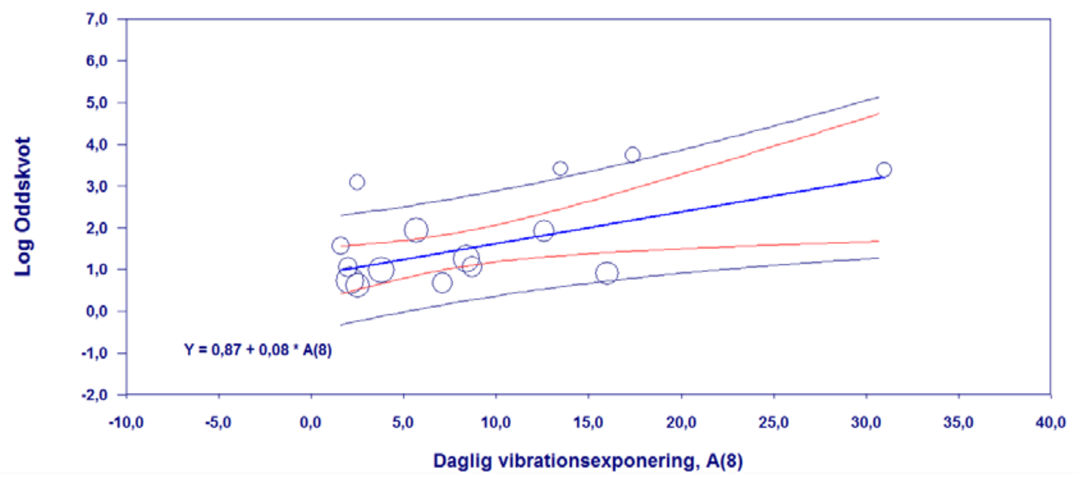


**Figur 18:** Metaregressionsanalys av sambandet mellan logaritmen för oddskvoten av neurosensorisk skada hos vibrationsexponerade och vibrationsexponeringen uttryckt som A(8) (random effekt; n=15). I figuren framgår också regressionslinjens 95% konfidensintervall. Cirklarnas storlek beskriver respektive studies vikt vid beräkningar av regressionen.

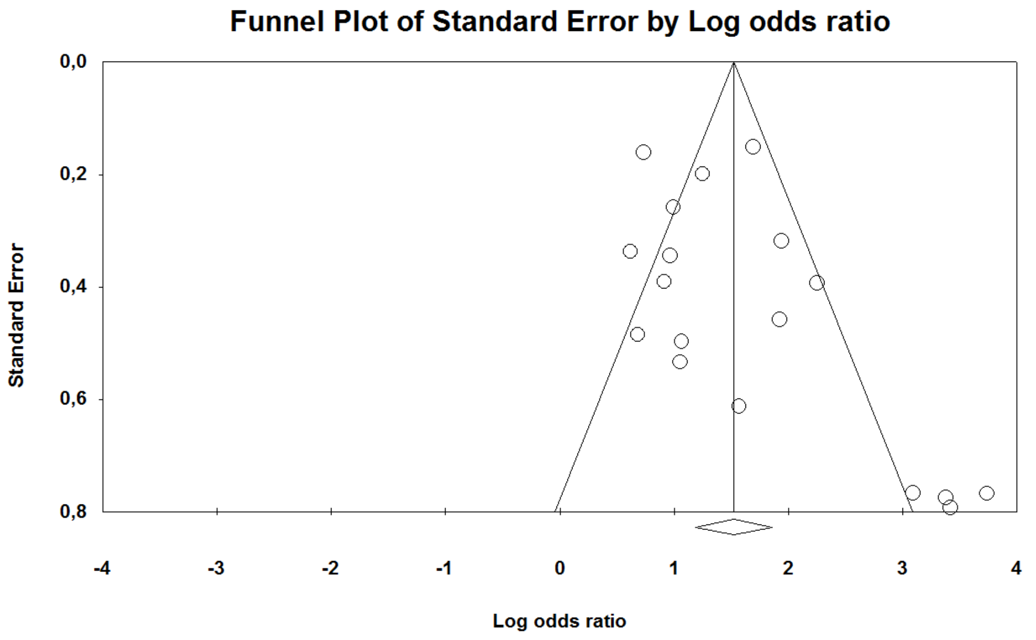


**Figur 19:** Trattdiagram (”funnel plot”) med pseudo 95% konfidensintervall för publikationsbias i studier av samband mellan förekomsten av neurosensorisk skada bland grupper som utsätts för HAV respektive icke-exponerade referensgrupper (Beggs test p = 0.04; Eggers test p=0.07; Trim och fyll metod beräknade att ingen studie saknas (random-effekt-modellen).

### Summering

Kunskapsöversikten identifierade 33 studier som uppfyllde inklusionskriteriena för att belysa sambandet mellan neurosensorisk skada och vibrationsexponering. Av dessa studier var 3 kohortstudier, 3 fall-kontrollstudier och 27 tvärsnitts-studier. Högkvalitativa studier finns inom alla tre undersökningsdesigner.

Prevalensstudierna visade att neurosensorisk skada förekom med en prevalens från 6% till 79% bland de vibrationsexponerade. Studierna visade på ett dos-responssamband där högre prevalenser för neurosensorisk skada var relaterade till högre vibrationsexponeringsnivåer. I metaanalysen inkluderades18 studier. Den sammanvägda risken för vibrationsexponerade var 4,58 (95% KI 3,28 – 6,38) jämfört med icke vibrationsexponerade. För de högkvalitativa studierna var motsvarande 7,78 (95% KI 4,28 – 14,15). Exponeringsbestämning med antal exponeringstimmar/dag gav den högsta oddskvoten (4,77; 95% KI 2,25 – 10,13) medan kumulerad dos gav lägre riskestimat. Meta-regressionsanalys indikerar ett dos-respons samband för neurosensorisk skada i relation till vibrations-exponeringsnivå. Enligt denna analys medför en exponeringsökning på 1 $m/s^2$ att relativa risken för neurosensorisk skada ökar med 8%.

## Karpaltunnelsyndrom

### Definition

Diagnosen ”karpaltunnelsyndrom” (KTS) sammanfattar det symptomkomplex och de kliniska fynd som uppkommer vid tryck på medianusnerven i handleden (karpaltunneln). Påverkan på medianusnerven kan uppkomma när nerven till-sammans med ett flertal hand/fingersenor passerar karpaltunnelns begränsade utrymme. Mekaniskt tryck från dessa senor eller ökad vävnadsvolym i tunneln från exempelvis ödem, vätskeretention, inflammation, muskelhypertrofi eller ganglion medför minskat utrymme för nerven. Skelettförändringar eller deformiteter i skelettet kan även minska kanalens utrymme.

### Diagnostiska överväganden och risk för bias

KTS innefattar i varierande grad manifestationer i form av smärta, parestesier, ”domningar”, känselnedsättning, kraftnedsättning och eventuell muskelatrofi i medianusinnerverat område. I de fall där det föreligger neurogena manifestationer med en utbredning förenlig med medianusnervens utbredningsområde distalt om karpaltunneln, alternativt i tidiga fall endast nattliga domningar samt en ökad nervretbarhet, kan det *kliniska kriteriet* för KTS vara uppfyllt. Elektrodiagnostiska undersökningar fraktionerat över karpaltunneln som påvisar en försämrad funk-tion distalt om karpaltunneln stärker diagnosen. Tillräcklig hand-arm överförd vibrationsexponering (*exponeringskriterium*) skall ha föregått skadan (*tidssam-bandskriterium*), samtidigt som det inte framkommit någon annan känd faktor som kan öka risken för KTS (*exklusionskriterium*).

### Gradering av ”karpaltunnelsyndromets” allvarlighetsgrad

KTS medicinska allvarlighet stadieindelas i tidiga (early), mer avancerade (intermediate) och sent stadium (late) (104). Klassificeringen kan alternativt även anges som stadier från 1 till 3 (105). Tidigt stadium kännetecknas av intermittenta symptom, nattliga domningar och väsentligen besvärsfrihet under dagtid. Mer avancerad KTS kännetecknas av bestående symptom innefattande parestesier och domningar även dagtid. Sent stadium kännetecknas av bestående nedsättning av sensorisk och motorisk funktion, ev. med muskelatrofi och smärta. Elektrodiagnostik (nervledningshastighetsmätning, elektromyografi) medger möjlighet att kvantifiera graden av den elektrodiagnostiska avvikelsen. Ingen av de ingående studierna i denna översikt har graderat allvarlighetsgraden på KTS-syndromet. I de fall elektrodiagnostik ingått ger resultatet ett mått på graden av entrapment. Grunddefinitionen av KTS varierar även mellan olika kliniska företrädare och mellan olika vetenskapliga rapporter (106). Flertalet epidemiologiska studier använder den konsensus-definition som Rempel och medarbetare föreslagit (107). Falldefinitionen bygger på följande fyra kriterier: 1. Förekomst av symptom förenliga med KTS graderat enligt symptomutbredning (klassiska symptom/troligen förenligt med KTS, symptom möjligen förenliga med KTS respektive symptom sannolikt ej förenliga med KTS), 2. Symptom nattetid, 3. Kliniska undersökningsfynd vid Phalens test respektive Tinells test samt 4. Elektrodiagnostiska undersökningsresultat.

### Möjlig bias till följd av alternativa orsaker till karpaltunnelsyndrom.

Ett antal individfaktorer och exponeringar som kan förekomma i arbetslivet har i systematiska kunskapsöversikter relaterats till en ökad risk för karpaltunnelsyndrom. Dessa exponeringsfaktorer innefattar arbete med vibrerande maskiner, upprepade, repetitiva kraftgrepp och kraftiga handledsrörelser (20, 21). Systematiska kunskapsöversikter med meta-analys bekräftar dessa samband (108).

### Möjlig bias till följd av interaktion från andra exponeringsfaktorer

Individfaktorer associerade till KTS innefattar övervikt, kön, hormonstatus, ålder (109) och tobaksbruk (110). Individer med neuropati till följd av till exempel diabetes, ämnesomsättningsrubbningar eller vitaminbrist (en detaljerad beskrivning framgår av bilaga 5) har även en ökad risk och sårbarhet för KTS. Personer med inflammatoriska reumatiska sjukdomar uppvisar även en ökad risk för KTS. Ett möjligt samband mellan specifikt Raynauds syndrom och KTS har även diskuterats (111).

### Resultat av den systematiska litteratursökningen för karpaltunnelsyndrom

Resultatet av den systematiska litteraturgenomgången för de studier som behandlat KTS presenteras nedan i tabell 8. Av tabellen framgår dels vår skattning av risk för bias (kvalitetspoäng) avseende diagnosens tillförlitlighet

för ”karpaltunnelsyndrom” samt dels den totala summan av kvalitetspoäng. Totalsumman är summan av kvalitetspoängen för diagnos adderat till kvalitetspoängen för ”studie-design” och ”exponering”.

Sammanställningen (tabell 8) visar att de 7 studier som behandlat KTS har en variation av diagnossumma mellan 1 och 11 för KTS.

**Tabell 8:** Ingående studier på karpaltunnelsyndrom samt deras bedömda risk för bias (kvalitetspoäng) avseende diagnosen ”KTS” (diagnos summa) samt totalsumma när kvalitetspoängen för bedömningen diagnos KTS, ”studie metod” och ”exponering” adderats (Total summa). Studierna presenteras i fallande ordning utifrån den totala poängsumman. Högre poäng indikerar högre ”kvalitet” vilket indikerar mindre möjlig risk för bias. Vidare framgår studiedesign för respektive studie.

| Studie | Referens | Design | Diagnos summa | Total summa |
|---|---|---|---|---|
| Bovenzi, 2000* | (79) | Fall-kontroll | 11 | 26 |
| Sanden, 2010 | (54) | Tvärsnitt | 11 | 22 |
| Nilsson, 1994 | (27) | Tvärsnitt | 11 | 24 |
| Bovenzi, 2005* | (24) | Tvärsnitt | 10 | 19 |
| Bovenzi, 1994* | (88) | Tvärsnitt | 6 | 17 |
| Edlund, 2013* | (102) | Kohort | 1 | 17 |
| Gerhardsson, 2005 | (46) | Tvärsnitt | 6 | 15 |

*Studien är med i meta-analysen

*Prevalens av karpaltunnelsyndrom i relation till publiceringsår*

Figur 20 visar prevalens för KTS från de fyra studier där uppgifter om förekomst redovisats eller kunnat beräknas. KTS har varierat mellan 7% och 35% i de olika studierna med ett genomsnitt på ca 18%.

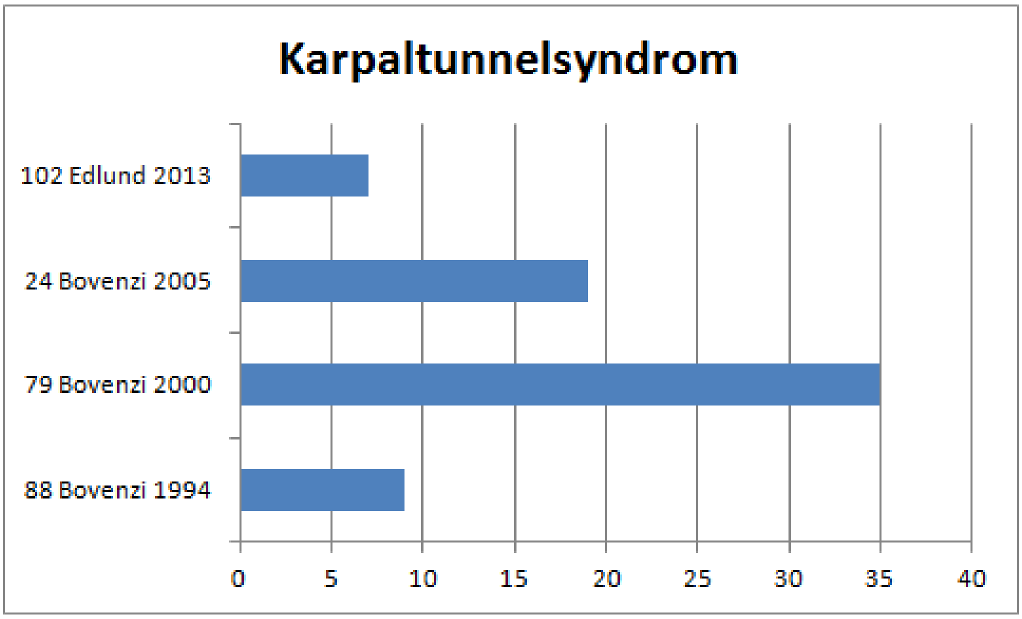


**Figur 20:** Studier med uppgift om prevalens av karpaltunnelsyndrom i den undersökta studiepopulationen sorterade efter publiceringsår.

## Resultat från meta-analys på studier av karpaltunnelsyndrom

*Resultat av meta-analys av grupper exponerade kontra oexponerade*

I meta-analysen inkluderas 4 av de 7 studierna på KTS. Den sammanvägda risken gav en oddskvot på 2,93 med en heterogenitet av 0 % (*p*= 0,93) (figur 21). Figuren visar också i en ”forest plot” resultaten från metaanalysen på studier när vi jämför risken för KTS mellan grupper exponerade för HAV kontra oexponerade referensgrupper. Vidare framgår den relativa betydelsen varje studie har i meta-analysen.

Trattdiagram (”funnel plot”) för de studier som ingår i vår metaanalys presenteras i figur 22. Resultatet visar att studierna fördelas något osymmetriskt kring den beräknade effekten men inga statistiska beräkningar har genomförts eftersom antalet studier är för få.

## Summering

Kunskapsöversikten identifierade 7 studier som uppfyllde inklusionskriteriena för att belysa sambandet mellan karpaltunnelsyndrom och vibrationsexponering. Av dessa studier var 1 kohortstudie, 1 fall-kontrollstudie och 5 tvärsnittsstudier. Prevalensen av KTS varierade från 7% till 35% bland de vibrationsexponerade.

I meta-analysen inkluderades 4 studier. Den sammanvägda relativa risken för vibrationsexponerade var 2,93 (95% KI 1,74 – 4,95) jämfört med oexponerade.

| Studie namn | Odds-kvot | 95%KI för oddskvot Lägre gräns | Övre gräns | Oddskvot och 95% konfidensintervall | Relativ betydelse i analysen |
|---|---|---|---|---|---|
| 79 Bovenzi, 2000* | 3,05 | 1,03 | 9,02 | | 23,31 |
| 24 Bovenzi, 2005* | 2,70 | 1,12 | 6,49 | | 35,51 |
| 88 Bovenzi, 1994 | 3,43 | 1,42 | 8,28 | | 35,25 |
| 102 Edlund 2013* | 1,62 | 0,19 | 13,84 | | 5,94 |
| ***Sammanvägt*** | 2,93 | 1,74 | 4,95 | | |

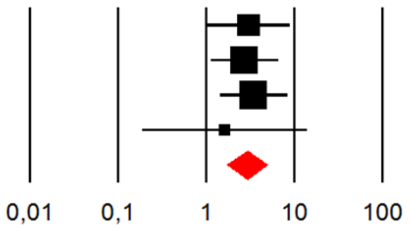


**Figur 21:** Statistik och ”forest plot” med sammanvägning från ”random – effekt” metaanalys av förekomsten av KTS mellan grupperna som utsätts för HAV respektive icke-exponerade referensgrupper. Storleken på fyrkanterna för de individuella studierna är proportionell mot den studiens betydelse i analysen. Den röda diamanten anger den sammanvägda risken för alla studier. Studierna har sorterats i ordning från högsta till lägsta kvalitetspoäng enligt tabell 8. Asterisk (*) indikerar att studien presenterat data som gjort det möjligt att beräkna ojusterad oddskvot.

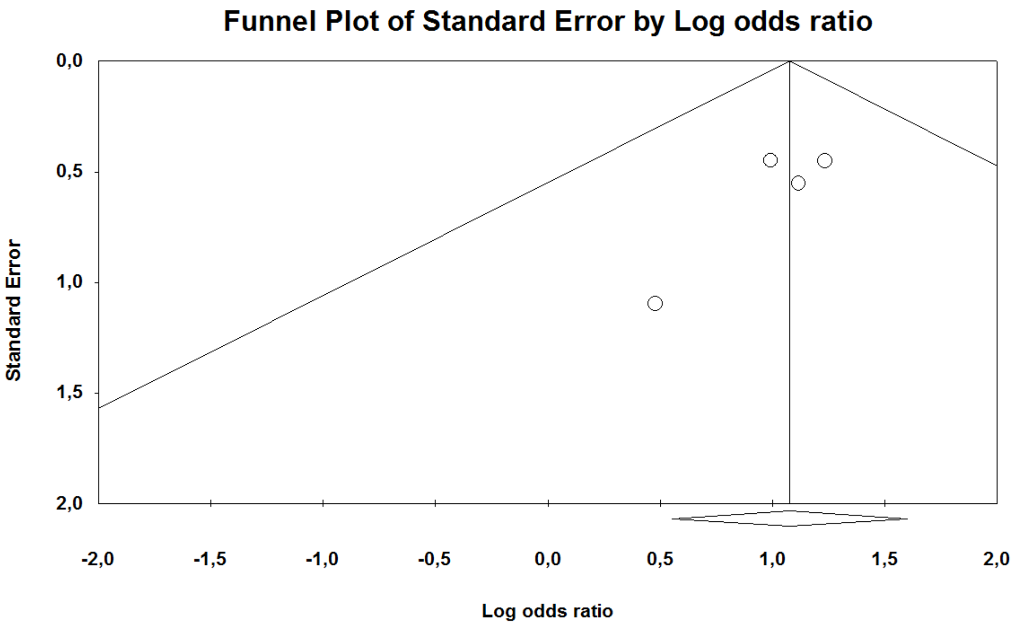


**Figur 22:** Trattdiagram (”funnel plot”) med pseudo 95% konfidensintervall för publiceringsbias i studier av samband mellan förekomsten av KTS bland grupper som utsätts för HAV respektive icke-exponerade referensgrupper

# Diskussion

Den beskrivande sammanvägningen av resultaten liksom den statistiska syntesen av sambandet mellan exponering för hand-arm överförda vibrationer och kärl-skada (Raynauds fenomen) befäster i båda fallen ett risksamband. Motsvarande stöd får sambandet mellan vibrationsexponering och neurosensorisk skada. Karpaltunnelsyndrom och vibrationsexponering indikerar även ett risksamband, men för detta samband är antalet studier ingående i det vetenskapliga underlaget lågt. Detta gör sistnämnda samband mer känsligt för bias. Riskernas storlek estimeras i denna kunskapsöversikt, som täcker den vetenskapliga litteraturen fram till september 2014, till 6,9 för de studier på Raynauds fenomen som bedömts ha låg risk för bias. Motsvarande risk för neurosensorisk skada är 7,4 medan motsvarande för karpaltunnelsyndrom är 2,9. Meta-regressionsanalys indikerar ett dos-respons samband för Raynauds fenomen i relation till vibrations-exponeringsnivå. Analysen visar att sambandet är tillförlitligt och enligt denna analys är koefficienten för A(8) 0,09, vilket betyder att för varje ökning med 1 m/s$^2$ ökar oddskvoten för Raynauds fenomen med 9%. Motsvarande riskökning för neurosensorisk skada är 8%. Sambandet uttrycker relationen mellan förekomst av skada och exponeringsnivå. Dos-respons samband visar relationen mellan exponeringsnivå och antal drabbade individer med Raynauds fenomen, neuro-sensorisk skada och karpaltunnelsyndrom. Dos-respons sambandet speglar därmed risken för skada i undersökningsgruppen. Ett motsvarande samband

mellan dos och effekt speglar relationen mellan exponeringsnivå och olika typer av utfall och/eller utfallets ”allvarlighetsgrad” hos enskild exponerad. Kunskapsunderlaget tillåter inte bedömning av dos-effekt samband. Detta innebär att kunskapsöversikten inte ger svar på frågan om hur svår skadans omfattning blir i det enskilda fallet, när exponeringen ökar.

Rapporten utgör det första exemplet inom området HAVS på systematisk evidensbaserad kunskapsöversikt. Den är dessutom den första sammanställningen där statistisk syntes (meta-analys inklusive meta-regression) använts på HAVS. Den här aktuella kunskapsöversikten skiljer sig därför från flertalet tidigare översikter, främst genom att den följt etablerade metodkriterier för evidensbaserad litteraturgenomgång samt genom att vi lagt stor vikt vid redovisad vibrationsexponering. Uppgifter om definierad vibrationsexponering har varit ett inklusionskriterium för att ingå i kunskapssammanställningen. Studier har endast inkluderats där en mätning eller ett estimat på vibrationsexponeringen funnits.

## Diagnostisk tillförlitlighet

*Möjliga utfallsbias*

Definitionen av ”skada” uppvisar betydande glidningar i diagnosdefinitioner från de tidiga studierna till senare tids studier. Inledningsvis användes ett kollektivbegrepp för syndromet ”HAVS”. Efterhand kom diagnoserna att specificeras i undergrupper av organdiagnoser (kärl-, respektive nervskador), för att därefter ytterligare undergrupperas. Nervskada har exempelvis kategoriserats i grovtrådsoch fintrådsneuropati samt i entrapmentsyndrom (KTS). Tidiga studier på nervskador torde därmed ha innefattat den skada som i senare tids studier benämns som karpaltunnelsyndrom. I vårt urval av artiklar finner vi studier på karpaltunnelsyndrom från mitten av 1990-talet och framöver (figur 6).

Under den studerade tidsperioden har även laboratorieundersökningar med elektrodiagnostik och köldprovokationstest utvecklats. Skadepanoramat har även förändrats. Ingående tidiga studier visar höga förekomster av kärlskada medan senare studier rapporterar en relativt högre förekomst av nervskada (figur 23). En observation som kan spegla en verklig förändring i skadeförekomst, men som även kan bero på uppmärksamhetsbias liksom publikationsbias.

Den diagnostiska upplösningen varierar även mellan olika studier och över tid. Ett flertal studier definierar utfallet enbart utifrån symptombeskrivning, andra använder även semiobjektiva respektive objektiva test vid diagnos. Ett antal studier skiljer på lindrig skada mot allvarlig skada genom klassificering, men huvuddelen av studierna anger endast förekomst av symptom, vilket medför att mer avancerad skada, i vissa fall kan komma att jämställas med lindrigare skada.

Sammantaget innebär detta att utfallen mellan olika tidsperioder uppvisar (figur 8 och 14) betydande variabilitet. Risken för begreppsglidning eller diagnosbias har vi i kunskapsöversikten försökt kontrollera vid bedömningen av kvalitetspoäng. Metaanalysen visar att det föreligger skillnader mellan studier med lägre risk för bias (=hög kvalitet) jämfört med studier där risken för bias kan vara större.

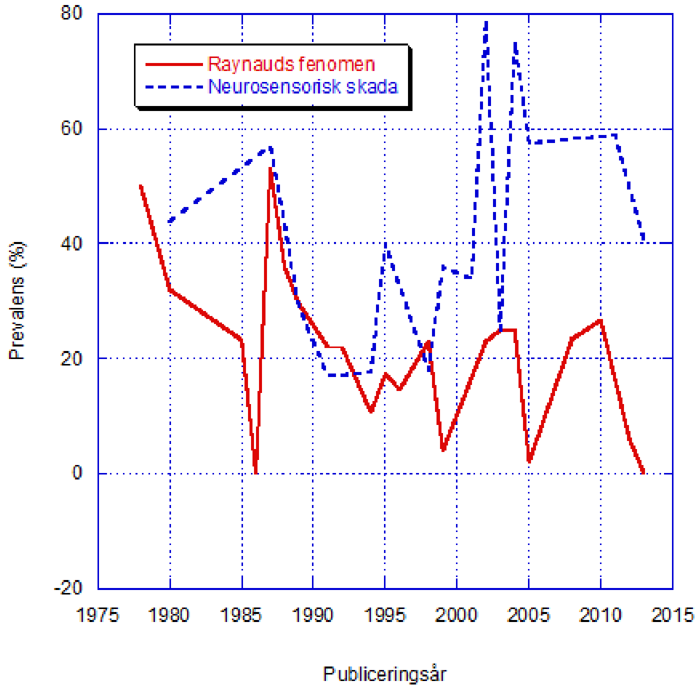


**Figur 23:** Prevalens i de studier som studerat Raynauds fenomen respektive neurosensorisk skada i relation till publiceringsår. I de fall flera studier publicerats samma år har medelvärdet av prevalensen beräknats.

För studier med lägre risk för bias finner vi en sammanvägd risk för Raynauds fenomen på 6,8 medan motsvarande för studier med lägre kvalitet är 3,6. Relationen är likartad för neurosensorisk skada med 7,8 mot 3,3. Vi finner däremot obetydliga skillnader i risknivåer vid sammanvägningen mellan tidiga studier jämfört med senare studier. Ett resultat som kan ha påverkats av låg precision på tids-uppgiften, då publiceringdatum och tidpunkt för undersökningens genomförande i många exempel skiljer sig kraftigt åt. Många publikationer redovisar studier som genomförts för längre tid sedan.

## Analytisk tillförlitlighet

*Möjliga exponeringsbias*

Vibrationens fysikaliska karaktäristika definieras av dess egenskaper i frekvens, intensitet och karaktär. Mätinstrument, elektronik och mätmetodik har varierat över tid, men har sedan 1976 standardiserats enligt ISO (ISO-5349). De tydligaste metodskillnaderna utgår från mätning av dominerande riktning till summavektor, uttryckt som initialt 4-timmars ekvivalent och sedermera 8-timmars ekvivalent värde och med eller utan frekvensvägning. I rapporten har vi genomgående räknat om alla exponeringar till 8-timmars ekvivalent frekvensvägd vibration, vilket medger jämförbarhet mellan olika studier. Senare tids epidemiologiska- (112), laboratorie- (113) och djurexperimentella (114) forskningsstudier ifrågasätter emellertid nuvarande frekvensvägning.

Exponeringsnivån för olika studier har beräknats som gruppmedelvärde och ej som individuell kumulativ dos. Detta innebär att den som uppvisar symptom kan ha erhållit dessa i en tidigare exponering, alternativt tidigare varit allt från kraftigt till obetydligt exponerad. Vidare framgår av redovisningen att exponeringen skett vid många olika exponeringsnivåer vilket innebär att de som i en studie betraktas som lågexponerade i en annan studie skulle betraktats som högexponerade. Dessutom har vi i vår meta-analyser utnyttjat riskkvoter som varit både justerade och ojusterade för andra kända riskfaktorer

Kronologiskt tidiga studier på sambandet mellan vibrationsexponering och skada omfattar främst studier på gruvarbetare, slipare och skogsarbetare. Manuellt skogsarbete utfördes tidigare företrädesvis på vintern, varvid exponering för kyla och väta, motoravgaser och lokal belastning på handen utgjorde samtidiga exponeringar. Vid introduktionen av tryckluftsborrar tillkom en tryckluftsrelaterad köldexponering av händerna. Den ogynnsamma exponeringen för såväl vibrationer som kyla ökade, när tekniken utvecklades så att maskinerna kunde användas fritt handhållna. Vi finner även vid litteraturgenomgången att skador och besvär tidsmässigt börjar rapporteras när de tryckluftsdrivna maskinerna blev handhållna och inte från den tid när de första ”släpbara” maskinerna kom i bruk. Handintensivt arbete, kyla, tryck och kraftgrepp kan alla utgöra samverkande faktorer för uppkomst av kärl- och nervskada. Kyla är, förutom en confounder för uppkomst av båda Raynauds fenomen och neurosensorisk skada, även en utlösande faktor för attacker av vita fingrar och köldintolerans.

Väsentligen alla studier har genomförts i den klimatzon (figur 5) som uppvisar fyra årstider; sommar, höst, vinter och vår. Medeltemperaturen ligger under den kallaste månaden vanligen under +6° Celsius, och under den varmaste över +10° Celsius. Studier har visat att ”vita fingrar” inte manifesterar sig i varmt klimat (90). Däremot kan nervskada uppstå. Avsaknad av HAVS-studier från varmare klimatzoner kan dölja en underrapportering av vibrationsskador från dessa områden.

Vi har i rapporten identifierat de branscher och övergripande nivåer (figur 7) som exponeringsmätningar i dessa branscher uppvisar och eftersökt ifall riskbedömningsmodellerna tagit hänsyn till eventuella förväxlingsfaktorer (confounders). För att undersöka huruvida andra individrelaterade faktorer kan förklara uppkomst av skada, har vi eftersökt om studierna tagit hänsyn till andra effektmodifierande faktorer eller förväxlingsfaktorer. Vi har sökt uppgifter i studierna om författarna tagit hänsyn till andra sjukdomar och ifall vanliga laboratoriescreeningar för inflammatorisk sjukdom respektive polyneuropati har förekommit. Resultatet har vi uttryckt i termer av hög eller låg risk för diagnosbias (kvalitetsskattning).

*Möjliga uppmärksamhetsbias*

Tecken på ”vita fingrar” (Raynauds fenomen) uppmärksammas lättare än tecken på nervskada av både den drabbade och av sjukvården. Litteraturen kan i och med detta spegla ett utfalls-bias. En neurosensorisk störning kan föreligga utan

samtidiga besvär i form av ”vita fingrar” och utan att den drabbade själv uppmärksammar nervstörningen.

*Möjliga metodbias*

Vid vår primära systematiska litteratursökning av publikationer följde vi konventionell, elektronisk databasbaserad litteratursökning i ett flertal olika sökmotorer (exempelvis PubMed). Erfarenhet liksom rekommendation från PRISMAs riktlinjer säger att en väl preciserad fördefinierad söksträng bör användas vid sådan litteratursökning. Trots bred sökning med överlappande termer fann vi vid efterföljande kvalitetsanalys, att de primära sökresultaten utelämnat ett stort antal väsentliga referenser. Vår totala sökningsprecision uttryckt som NNR (Number Needed to Read) var 83.

Uppföljande analys visade att sökning med filter för engelsk text (”language”) respektive för ”human” medförde att ca 10 % av artiklarna kom att filtreras bort beroende på att de ej indexerats för språk. De artiklar som initialt inte kom med hade inte uteslutits på grund av att de inte uppfyllde övriga söktermer utan på grund av kravet på indexering för språk. Efter manuell genomgång kom den slutliga sökningen och därmed de som rapporteras i denna rapport att innefatta ytterligare 16 relevanta referenser som uppfyllde sökkriterierna. Valda söktermer innefattar textsökning samt indexerad sökning även högt i sökträdet. Sökningen har syftat till att ge hög sensitivitet, vilket balanserats av att artiklar successivt exkluderats utifrån våra kriterier. För manuell kontroll av artiklar utan indexering för språk var NNR = 100.

Genom detta breda angreppssätt, våra kvalitetstest och förnyade sökningar kompletterat med manuell genomgång för ej indexerade rapporter bedömer vi, att den slutliga litteratursökningen ger en valid bild av kunskapsunderlaget som det speglas i den västerländska vetenskapliga litteraturen. Vår begränsning till enbart engelskspråkig litteratur medför eventuellt att relevant östeuropeisk, fransk, tysk, spansk och kinesisk vetenskap förbisetts. En serie av återkommande internationella HAVS-möten inleddes 1972 i Dundee i Skottland och har sedan dess genomförts 12 gånger med deltagare från stora delar av världen. Dessa återkommande internationella hand-arm vibrationskonferenser bedömer vi ha medfört att flertalet studier kommit att publicerats även på engelska.

Litteratursökningsprocessen har försvårats av duplicerings-bias och citerings-bias. Ett fåtal författare har upprepat publicerat rapporter baserat på samma studiepopulation. Vid dubbleringar har endast den publikation som vi i konsensus bedömt som mest relevant inkluderats i metaanalyser. Härigenom har ett antal publikationer från främst italienska undersökningar utgått. För att ge läsaren transparent information om publiceringsbias, har studiernas publicerings-bias gestaltats som ”funnel-plots” för standard error i relation till log odds ratio som effect-size för vart utfall. Detta med förbehållet att standard-error är relaterat till undersökningspopulationens log-odds (115). Tecken på publicerings bias framkommer för vita fingrar och nervskada. Detta kan tala för att riskestimatet möjligen överskattas. Tydligast framgår detta i ett flertal publikationer med få deltagare men med höga risker.

Den statistiska syntesen bygger på meta-analys enligt ”Comprehensive meta analyses” och i de fall där utfall saknas i någon cell har en konservativ metod med addering av ”1” i alla celler utnyttjats. Metaanalyser där justering enligt denna metod använts gäller studier där det exempelvis inte förekom några fall med ”vita fingrar” (exempelvis studier på förekomst av ”vita fingrar” i tropikerna, (90). Beräkningar där studier med 0-celler uteslutits och där addering av ett fall i samtliga celler skett, visar att vi får en förändring i risk för Raynauds fenomen från det sammantagna värdet på 3,6 till 3,1 och för subgruppen med studier med hög kvalitet från 6,8 till 6,5.

Den vetenskapliga dokumentationen på vibrationsskador domineras av tvärsnittsstudier. Vid beskrivande bedömning av enskilda studiers riskstorlek har vi funnit höga prevalenstal som utmanar användning av oddskvot. Den överskattning av risk som detta kan ha inneburit har vi beaktat i den sammanvägda bedömningen. Vi har i vår kvalitetsgradering vägt typ av studie och vid metaanalys tagit hänsyn till design vid bestämning av effektstorlek.

### Riskbedömning i relation till ISO-5349-annex och Europeiska vibrationsdirektivet

Kunskapssammanställningen visar på en ökad risk för skada (kärl- och nervskada) med ökad vibrationsexponering. Vid sammanställningen av förekomsten av skada mot exponeringsnivå visar neurosensorisk skada ett starkare samband jämfört med motsvarande för Raynauds fenomen. För Raynauds fenomen har ett flertal studier rapporterat mycket låg förekomst trots höga exponeringsnivåer (figur 9). Motsvarande kan inte ses för neurosensorisk skada (figur 17). En trolig förklaring till denna skillnad är temperatur- och klimateffekter, men det kan även finnas ytterligare faktorer som påverkat detta. Bedömning av risken för Raynauds fenomen kan därför behöva kompletteras med studier på andra effektmodifierande faktorer.

Riskbedömningen i annexet till ISO-5349 bygger på prevalens av Raynauds fenomen i relation till vibrationsnivå och exponeringsår. Exponeringen uttrycks som ekvivalent 8-timmars frekvensvägd exponering. Bedömningen utgår från latenstid, där ingående gruppers exponeringsnivåer har ett A(8) som ligger mellan 9 och 20 $m/s^2$ med dagliga exponeringstider på mellan 9 och12 timmar med endast kortare avbrott. Den dagliga exponeringstiden var därmed relativt konstant. Problem uppstår därmed i den interpolering som sker till lågdosområdet.
ISO 5349 presenterar ett samband för att fastställa ekvivalent 8-timmars frekvensvägd exponering när exponeringstiden avviker från 8 timmar. I denna beräkning ges exponeringen större betydelse än exponeringstiden. Vibrationsnivån går att mäta med teknisk utrustning, medan tiden antingen skattas subjektivt av arbetstagaren eller objektivt genom mätningar. Diskrepansen mellan dessa bestämningar av exponeringstiden kan vara stor och då speciellt vid kortare exponeringstider. Genom att ISO 5349 bygger på ett samband som definierats utifrån heldagsexponeringar är det för närvarande att föredra om exponeringstiden kan bestämmas så korrekt som möjligt om den modellen används. Senare tids

forskning har dock visat att hälsorisker kan påvisas vid exponeringsnivåer under de nivåer som riskbedömningsmodellen täcker. En prospektiv studie har visat ökad risk för Raynauds fenomen vid så låga nivåer som 1,0 $m/s^2$ (A(8) värde) (85) och en tvärsnittsstudie vid motsvarande 0,6 $m/s^2$ (A(8) (43). ISO-standarden bygger på latenstider och är därigenom pseudokohortbaserat. Våra resultat visar att dos-respons sambanden mellan vibrationsexponering och Raynauds fenomen respektive neurosensorisk skada uppvisar skilda funktioner. En bedömning av risk för neurosensorisk skada utifrån den riskbedömningsmodell som presenteras i annexet till ISO-5349 är därmed vilseledande. Det finns följdaktligen ett stort behov av att utveckla en riskbedömningsmodell specifikt anpassad för neurosensorisk skada.

Resultaten från vår sammanställning medger att följande alternativa samband till ISO-5349 mellan HAV exponering och Raynauds fenomen och neurosensorisk skada kan beräknas. Sambandet bygger på uppgifter om de olika studiernas dagliga vibrationsexponering uttryckt som A(8), prevalensen av besvär samt gruppens medelvärdiga exponeringstid (år). Vi har antagit ett linjärt samband vid utvecklingen av besvär. Exempelvis i en grupp exponerade är prevalensen för besvär 40% och gruppens medelvärdiga exponeringstid för HAV är 20 år. Tid innan 10% av exponerade uppvisade besvär kan då beräknas till 5 år. Uppgiften om A(8) och antal år till 10% prevalens kan därmed användas för att konstruera ett samband. Detta estimerade samband presenteras i figur 24 för Raynauds fenomen respektive neurosensorisk skada. Vidare har i figuren motsvarande kurva för ISO 5349-1 (figur 2) tagits med.

Av figuren framgår att spridning i beräknade värden är stor. Vid de antaganden vi gjort framgår utifrån beräknade regressionslinjer att vid en daglig exponeringsnivå på 10 $m/s^2$ uppträder en tioprocentig prevalens av Raynauds fenomen efter 6 år och för neurosensorisk skada efter 2 år, skillnaden är således en faktor 3. Vidare framgår att båda regressionslinjerna löper i stort sett parallella och har en dålig överensstämmelse gentemot prediktionen utifrån ISO 5349-1.

ISO 5349-1 är grunden för det europeiska vibrationsdirektiv (116) som används i Sverige och inom EU för värdering av huruvida riskerna med exponering för HAV är acceptabla från samhällets perspektiv. Det europeiska vibrationsdirektivet beskriver två exponeringsvärden, ett insatsvärde och ett gränsvärde. Värdena gäller daglig, frekvensvägd exponering under en period av 8 timmar. Om insatsvärdet överskrids är arbetsgivaren tvungen att utarbeta och genomföra ett program för tekniska och/eller organisatoriska åtgärder. Syftet med programmet ska vara att minska exponeringen för vibration till ett minimum. Arbetstagare får inte utsättas för vibrationsnivåer som ligger över gränsvärdet. Gränsvärdet för den dagliga exponeringen, normaliserat till en referensperiod på 8 timmar, är 5,0 $m/s^2$ och insatsvärdet 2,5 $m/s^2$. Arbetsmiljöverket har överfört vibrationsdirektivet till en svensk föreskrift (117).

Ibland presenteras vibrationsbelastningen som kumulativ exponering för varje exponerad över hela dennes livstid som år multiplicerad med dagliga accelerationen. Detta utvärderingssätt är fortfarande under utveckling. Våra resultat visar

att hälsorisker kan uppkomma vid nivåer betydligt under de riktlinjer som vibrationsdirektivet idag förordar.

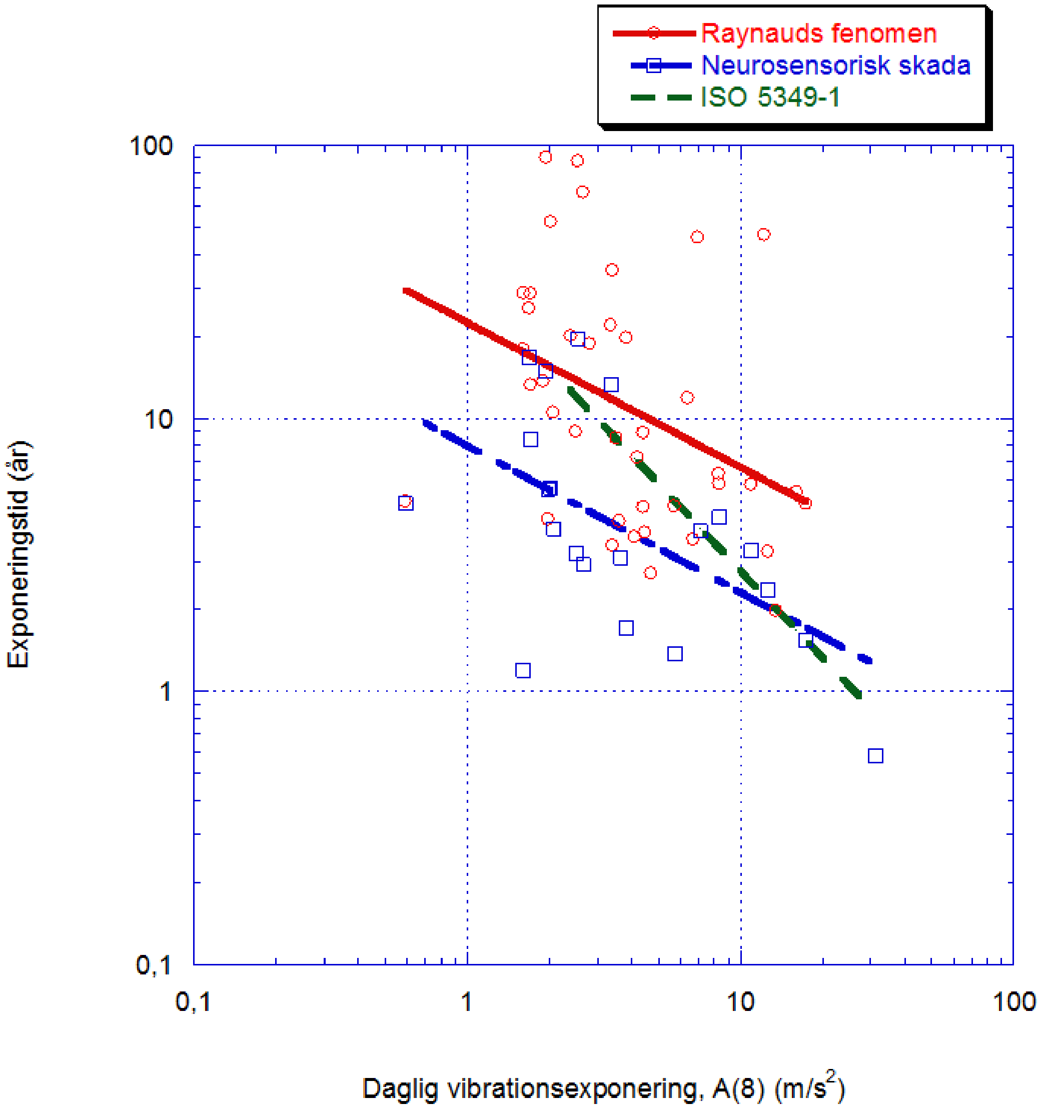


**Figur 24:** Beräknat samband mellan predicerad 10 % prevalens för Raynauds fenomen (25 studier 40 yrkesgrupper) respektive neurosensorisk skada (17 studier; 21 yrkesgrupper) som funktion av 8-timmar ekvivalent frekvensvägd acceleration och antal år med exponering. I figuren framgår samt den linjära regressionslinjen för de båda utfallen samt motsvarande kurva för ISO 5349-1 (figur 2) (Ekvationer:
$Raynauds\ fenomen\ (\%) = 10^{1.35+\ log_{10}(A(8)^{-0,53})}$, r=0.39;
Prevalens $Neurosensorisk\ skada\ (\%) = 10^{0.9+\ log_{10}(A(8)^{-0,54})}$, r=0,55)

## Angelägen information som saknats vid kunskapsgenomgången

### Uppmärksammade kunskapsluckor

Etiologin till flertalet manifestationer på vibrationsrelaterade skador pekar på påverkan /störning av det autonoma nervsystemet. Kunskapsöversikten över nervskador har inte kunnat identifiera någon studie som specifikt adresserat frågan om störningar i det autonoma nervsystemet. Störningar i neurosensorisk funktion för perception av vibrationer, värme och kyla förekommer däremot. Autonoma nerver inkluderande C-fibrer utgör tillsammans med A- δ fibrer och A- β fibrer sammantaget ca 70-80% av alla kroppens nervfibrer. Trots dessa nervers stora förekomst, de fina trådarnas skörhet och avsaknaden av skyddande myelin, så har störningar av det autonoma nervsystemet inte studerats systematiskt. Överlappningen i funktion mellan informationsöverföringen i C-fibrer för smärta och perception av värme, liksom manifestationer av köldöverkänslighet motiverar ett ökat fokus även på smärtproblematik, köldöverkänslighet och autonoma manifestationer vid vibrationsskada.

Litteraturgenomgången avslöjar även att det saknas forskning om interaktioner mellan olika sjukdomar och vibrationsskadesyndromets olika manifestationer. Effekten av sådan samsjuklighet (co-morbidity, multi-morbidity) med vibrationsskada har under senare decennier fått ökad klinisk betydelse då förekomsten av kroniska sjukdomar ökar i en befolkning som arbetar till högre ålder. Interaktion kan uppstå inte bara mellan HAVS och olika sjukdomar utan även till den medicinering som ges för medicinska riskfaktorer (exempelvis behandling av blodtryck). Kunskap om hur olika sjukdomar samverkar (samsjuklighet) med uppkomst respektive försämring av HAVS liksom inverkan av basala kroppsfunktioner som sömn och kondition saknas för närvarande i litteraturen.

En fråga nära kopplad till samsjuklighet är frågan hur enskilda individers eventuella sårbarhet för vibrationsexponering modifierar utvecklingen av HAVS. Samsjuklighet, åldersrelaterade modifierande faktorer för såväl unga som äldre, samt eventuell interaktion med läkemedel liksom andra vasoaktiva eller nervstörande exponeringar har förbisetts i forskningen. Riskuppgifter om faran av att arbeta med vibrerande maskiner som åldrad arbetare eller som mycket ung saknar helt evidensbaserad kunskap. Kunskap om könshormoners inverkan och andra könsrelaterade effektmodiferare (till exempel blodtryck och antropometri) saknas även varför effekten av vibrationsexponering på män och kvinnor behöver studeras separat. Sårbarhetsfaktorer för uppkomst av HAVS-syndromets olika manifestationer behöver identifieras. Forskning med inriktning mot diagnostik med biomarkörer kan vara en sådan utvecklingslinje. En frågeställning nära kopplad till sårbarhetsfaktorer är prognos för skada när exponering upphör eller reduceras. Denna frågeställning ingick inte i vårt primära uppdrag för kunskapssammanställningen, men vi har vid vår systematiska litteraturgenomgång noterat att det finns en stor brist på välgjorda studier om prognos.

Interaktion mellan olika miljöexponeringar och HAVS finns i viss mån representerade i ett fåtal studier. Interaktionseffekter mellan vibrationsexponering

och bullerexponering, respektive vibrationsexponering och ergonomiskt belastande arbete i relation till Raynauds fenomen, är sådana exempel. Det saknas särskilt systematiska studier på kylans betydelse för uppkomst av både nerv- och kärlskada.

Endokrina systemets interaktion med uppkomst av kärl- och nervskador finns ej belyst i litteraturen. Det är känt att attacker av vita fingrar lättare uppträder vid den tid på dygnet när kortisol-systemet har ett ökat påslag. I vilken omfattning skaderisken för kärl- och nervskada är relaterad till när på dygnet man arbetar, i vilken mån cirkadisk rytm, respektive störd rytm till följd av nattarbete eller skiftarbete kan inverka, framgår inte av litteraturen. Kunskap om könshormoners inverkan och andra könsrelaterade effektmodiferare saknas i stor utsträckning och behöver studeras separat.

### Uppmärksammade brister i teoribildning

Väsentligen inga av de epidemiologiska studier som analyserats har satt resultaten i ett teoretiskt sammanhang. Hypotesbildning och teorier om samband mellan nerv- och kärlskada och dess etiopatofysiologi saknas väsentligen. En förenklad icke biologisk begreppsbildning om vibrationers effekt på kroppen dominerar. Effekten av exponering av vibrationer på kroppens organ tolkas främst i termer av krafter och energiöverföring. Exponeringens biologiska effekter förbises till följd av tolkningsföreträde från mekanistiskt teoretiserande, trots att det till exempel är känt sedan länge att lokal exponering ger lokal kärlpåverkan åtföljt av effekter även i övriga delar av kroppen (11). Förutom en lokal kärlpåverkan vid akut vibrationsexponering uppträder samtidigt effekter i hela kärlträdet. Vid den breda litteraturgenomgången av kärlskador fann vi enstaka studier som visade på ogynnsamma kardiovaskulära hälsoeffekter i form av hjärtinfarkt och slaganfall (stroke). Detta samband skulle kräva ytterligare forskning för att denna kärleffekt skall kunna utvärderas.

I litteraturen saknas studier som systematiskt sökt bestämma mekanismen för nerv- respektive kärlskada och som försökt bestämma skadans lokalisation. Det saknas till exempel studier som specifikt belyser vibrationers påverkan på perifera sensoriska enheter genom analyser av hudbiopsier, histopatologi av nerver, undersökning av postganglionär nervfunktion liksom beaktandet av centralnervös funktion och förändringar i hjärnans plasticitet. Endast ett mycket litet antal studier finns rapporterade som adresserar frågan om centralnervösa förändringar vid användning av vibrerande maskiner.

Hållpunkter har framkommit om att vibrationsskadans vaskulära komponent kan ingå i en generellt utbredd endotelskada, men etiopatologisk forskning på detta saknas. Histologisk metod har däremot kunnat påvisa mikroskopiska förändringar på kärl och kärlväggar. Det finns i den allmänna medicinska litteraturen stöd för att karpaltunnelsyndrom, triggerfinger, Dupuytrens kontraktur och tendinovaginiter alla kan ha en vaskulär komponent i sin patogenes. Vår litteratursökning har visat att synen på exempelvis Dupuytrens kontraktur i

relation till vibrationsexponering kommit att omvärderas i flera omgångar och att denna skada kan vara relaterad till arbete med vibrerande maskiner.

### Uppmärksammade metodsvagheter

Diagnostisk tillförlitlighet kräver metoder för standardiserade effektmätningar. Det saknas idag helt uppgifter om kvantitativa sensoriska mätmetoders prediktiva värde. Vi finner vid litteraturgenomgången att testmetodiken dessutom varierar mellan olika forskare samt att kvalitetssäkrade ”normalvärden” respektive definitioner på vad som kliniskt skall bedömas som ”patologiskt” saknas. Mot bakgrund av vibrationsexponeringens stora omfattning i Sverige och den bristande harmoniseringen mellan olika enheter vid testning och bedömning av skada krävs betydande forskning för att ta fram ”normalvärden” vars prediktiva värde bestäms utifrån enhetliga definitioner på skada.

Frekvensvägning ingår i nuvarande standard för exponeringsbestämningen av vibrationsnivåer. Frekvensvägningen bygger på uppgifter om människans upplevelse av vibrationer. Validiteten i relationen upplevelse av vibration och skada har i det internationella vetenskapssamhället ifrågasatts bland annat vid Ottawa 2011 frequency weightings workshop (112, 113, 114).

Tillämpbarheten av eventuell frekvensvägning behöver även belysas utifrån olika medicinska hälsoutfall. Det finns skäl att anta att etiopatologin skiljer sig mellan nerv- och kärlskada. Nuvarande ISO-standard utgår från att antal exponerade år med samma exponering, lika dag för dag, utgör den skadliga faktorn för Raynauds fenomen. Studier behövs för att visa om detta antagande om skadeuppkomst är giltigt även för neurosensorisk skada, karpaltunnelsyndrom och artros förutom för Raynauds fenomen.

Vibrationsexponering uttrycks i vår sammanställning som A(8) på gruppnivå. ISO-standarden anger exponering som antal år med daglig exponering enligt A(8). Effekten av exponering i termer av kumulerad exponering behöver beskrivas specifikt för de olika hälsoutfallen; kärl, nerver, muskler leder och senor. Frågan om i vilken utsträckning det är den högsta exponeringen som skadar eller om det är en kumulering av exponering över år som är den kritiska exponeringen står obesvarad efter vår genomgång av litteraturen, på samma sätt som frågan om inverkan från stötar och slag kan jämföras med kontinuerlig vibrationsexponering.

### Uppmärksammade brister i informationsspridning och prevention

Forskningsområdet om vibrationer saknar beforskning om hur kunskap kan spridas avseende:
att det idag endast finns begränsad symptomatisk lindring vid vibrationsskada,
att det för närvarande inte finns någon medicinsk åtgärd som kan bota skadan,
att prognosen för en etablerad vibrationsskada är utomordentligt dålig även om exponeringen upphör samt
att skador i stor utsträckning drabbar unga och att risken därför särskilt måste förebyggas.

Medicinsk screening och medicinska kontroller regleras enligt föreskrifter. Det saknas underlag för att evidensbaserat kunna föreslå på vilket sätt, för vilka funktioner, med vilka verktyg och hur ofta dessa undersökningar skall genomföras. Resultatbaserade rekommendationer för metoder och verktyg som kan användas av företag och företagshälsovård behöver utarbetas, liksom framtagning av uppgifter om hur risker kan kommuniceras med arbetsgivare, anställda, fackliga företrädare och skyddsorganisationer.

# Referenser


1. Agate JN, Druett HA, Tombleson JB. Raynaud's phenomenon in grinders of small metal castings; a clinical and experimental study. Br J Ind Med. 1946 Jul;3:167-74.
2. ISO/DIS 5349. Principles for the measurement and the evaluation of human exposure to vibration transmitted to the hand. Genevé, Schweiz: International Organization for Standardization1979.
3. Griffin MJ. Foundations of hand-transmitted vibration standards. Nagoya J Med Sci. 1994;57(Suppl.):147-64.
4. ISO 5349. Mechanical vibration - Guidelines for the measurement and the assessment of human exposure to hand-transmitted vibration. International Organization for Standardization. Genevé, Schweiz: International Organization for Standardization1986. Report No.: ISO 5349:1986(E).
5. ISO 5349-1. Mechanical vibration - Measurement and evaluation of human exposure to hand-transmitted vibration - Part 1: General guidelines. Draft International Standard. Genevé, Schweiz: International Organization for Standardization 2001 May 1999.
6. ISO 5349-2. Mechanical vibration - Measurement and evaluation of human exposure to hand-transmitted vibration - Part 2: Practical guidelines for measurement in the workplace. Draft International Standard. Genevé, Schweiz: International Organization for Standardization2001 May 1999.
7. Hirosawa I, Nishiyama K, Watanabe S. Temporary threshold shift of temperature sensation caused by vibration exposure. Int Arch Occup Environ Health. 1992;63(8):531-5.
8. Malchaire J, Piette A, Rodriguez DL. Temporary threshold shift of the vibration perception threshold following a short duration exposure to vibration. Ann Occup Hyg. 1998;42(2):121-7.
9. Rohmert W, Wos H, Norlander S, Helbig R. Effects of vibration on arm and shoulder muscles in three body postures. European journal of applied physiology and occupational physiology. 1989;59(4):243-8.
10. Malchaire J, Rodrigueq Diaz LS, Piette A, Goncalves Amaral F, de Schaetzen D. Neurological and functional effects of short-term exposure to hand-arm vibration. International Archives of Occupational and Environmental Health. 1998;71(4):270-6.
11. Bovenzi M, Griffin MJ. Haemodynamic changes in ipsilateral and contralateral fingers caused by acute exposures to hand transmitted vibration. Occupational and environmental medicine. 1997;54:566-76.
12. Burstrom L, Lundstrom R, Hagberg M, Nilsson T. Vibrotactile perception and effects of short-term exposure to hand-arm vibration. Ann Occup Hyg. 2009 Jul;53(5):539-47.
13. Bovenzi M, Apostoli P, Alessandro G, Vanoni O. Changes over a workshift in aesthesiometric and vibrotactile perception thresholds of workers exposed to intermittent hand transmitted vibration from impact wrenches. Occupational and environmental medicine. 1997;54:577-87.
14. Gerard MJ, Martin BJ. Post-effects of long-term hand vibration on visuo-manual performance in a tracking task. Ergonomics. 1999 Feb;42(2):314-26.
15. Lawson I, Burke F, McGeoch K, Nilsson T, Proud G. Hand-arm vibration syndrome. In: Baxter P, Aw T, Cockcroft A, Durrington P, Harrington J, editors. Hunters Diseases of Occupations. 10th ed. London: Hodder Arnold; 2010. p. 489 -512.
16. Hamilton A. A study of spastic anaemia in the hands of stonecutters. Industrial Accident Hygiene Service Bulletin. 1918:53-66.
17. Hamilton A. Exploring The Dangerous Trades - The Autobiography Of Alice Hamilton, M.D. Boston: Little Brown; 1943.

18. Loriga G. II lavoro con i martelli pneumatici. Bollettino del Ispettorato del Lavoro. 1911;2:35-60.

19. Seyring M. Maladies from work with compressed air drills. Bulletin of Hygiene. 1931;6(25).

20. van Rijn RM, Huisstede BM, Koes BW, Burdorf A. Associations between work-related factors and the carpal tunnel syndrome--a systematic review. Scand J Work Environ Health. [Research Support, Non-U.S. Gov't Review]. 2009 Jan;35(1):19-36.

21. Palmer KT. Carpal tunnel syndrome: the role of occupational factors. Best practice & research Clinical rheumatology. 2011 Feb;25(1):15-29.

22. Liberati A, Altman DG, Tetzlaff J, Mulrow C, Gotzsche PC, Ioannidis JP, Clarke M, Devereaux PJ, Kleijnen J, Moher D. The PRISMA statement for reporting systematic reviews and meta-analyses of studies that evaluate health care interventions: explanation and elaboration. Journal of clinical epidemiology. 2009 Oct;62(10):e1-34.

23. SBU. Utvärdering av metoder i hälso-och sjukvården: En handbok.: Statens beredning för medicinsk forskning (SBU); 2013.

24. Bovenzi M, Della Vedova A, Nataletti P, Alessandrini B, Poian T. Work-related disorders of the upper limb in female workers using orbital sanders. Int Arch Occup Environ Health. 2005 May;78(4):303-10.

25. Matsumoto T, Fukaya Y, Sakakibara H, Shibata E. Health status of the workers using mechanized hand tools in an electric parts' maker. Cent Eur J Public Health. 1995;3 Suppl:93-6.

26. Mirbod SM, Yoshida H, Komura Y, Fujita S, Nagata C, Miyashita K, Inaba R, Iwata H. Prevalence of Raynaud's phenomenon in different groups of workers operating hand-held vibrating tools. Int Arch Occup Environ Health. 1994;66(1):13-22.

27. Nilsson T, Hagberg M, Burstrom L, Kihlberg S. Impaired nerve conduction in the carpal tunnel of platers and truck assemblers exposed to hand-arm vibration. Scand J Work Environ Health. 1994 Jun;20(3):189-99.

28. Burstrom L, Jarvholm B, Nilsson T, Wahlstrom J. White fingers, cold environment, and vibration--exposure among Swedish construction workers. Scand J Work Environ Health. 2010 Nov;36(6):509-13.

29. Nilsson T, Burstrom L, Hagberg M, Lundstrom R. Thermal perception thresholds among young adults exposed to hand-transmitted vibration. Int Arch Occup Environ Health. 2008 Apr;81(5):519-33.

30. Bovenzi M. Vibration-induced white finger and cold response of digital arterial vessels in occupational groups with various patterns of exposure to hand-transmitted vibration. Scand J Work Environ Health. 1998 Apr;24(2):138-44.

31. Su AT, Maeda S, Fukumoto J, Darus A, Hoe VC, Miyai N, Isahak M, Takemura S, Bulgiba A, Yoshimasu K, Miyashita K. Dose-response relationship between hand-transmitted vibration and hand-arm vibration syndrome in a tropical environment. Occup Environ Med. 2013 Jul;70(7):498-504.

32. Anttonen H, Virokannas H. Hand-arm vibration in snowmobile drivers. Arctic Med Res. 1994;53 Suppl 3:19-23.

33. Palmer K, Crane G, Inskip H. Symptoms of hand-arm vibration syndrome in gas distribution operatives. Occup Environ Med. 1998 Oct;55(10):716-21.

34. Walker DD, Jones B, Ogston S, Tasker EG, Robinson AJ. A study of white finger in the gas industry. Br J Ind Med. 1985 Oct;42(10):672-7.

35. Altman DG, Bland JM. Interaction revisited: the difference between two estimates. BMJ. 2003 Jan 25;326(7382):219.

36. Ioannidis JP, Patsopoulos NA, Evangelou E. Uncertainty in heterogeneity estimates in meta-analyses. BMJ. 2007 Nov 3;335(7626):914-6.

37. Petitti DB. Approaches to heterogeneity in meta-analysis. Stat Med. 2001 Dec 15;20(23):3625-33.

38. Begg CB, Mazumdar M. Operating characteristics of a rank correlation test for publication bias. Biometrics. 1994 Dec;50(4):1088-101.

39. Egger M, Davey Smith G, Schneider M, Minder C. Bias in meta-analysis detected by a simple, graphical test. BMJ. 1997 Sep 13;315(7109):629-34.

40. Duval S, Tweedie R. Trim and fill: A simple funnel-plot-based method of testing and adjusting for publication bias in meta-analysis. Biometrics. 2000 Jun;56(2):455-63.

41. Carmeli Y, Samore MH, Huskins C. The association between antecedent vancomycin treatment and hospital-acquired vancomycin-resistant enterococci: a meta-analysis. Archives of internal medicine. 1999 Nov 8;159(20):2461-8.

42. Aiba Y, Yamamoto K, Ohshiba S, Ikeda K, Morioka I, Miyashita K, Shimizu H. A Longitudinal Study on Raynaud's Phenomenon in Workers Using an Impact Wrench. J Occup Health. 2012 Apr 29;54(2):96-102.

43. Barregard L, Ehrenstrom L, Marcus K. Hand-arm vibration syndrome in Swedish car mechanics. Occup Environ Med. 2003 Apr;60(4):287-94.

44. Brubaker RL, Mackenzie CJ, Hertzman C, Hutton SG, Slakov J. Longitudinal study of vibration-induced white finger among coastal fallers in British Columbia. Scand J Work Environ Health. 1987 Aug;13(4):305-8.

45. Futatsuka M, Ueno T. Vibration exposure and vibration-induced white finger due to chain saw operation. Journal of occupational medicine : official publication of the Industrial Medical Association. 1985 Apr;27(4):257-64.

46. Gerhardsson L, Balogh I, Hambert PA, Hjortsberg U, Karlsson JE. Vascular and nerve damage in workers exposed to vibrating tools. The importance of objective measurements of exposure time. Appl Ergon. 2005 Jan;36(1):55-60.

47. Harazin B, Langauer-Lewowicka H. Raynaud's phenomenon in different groups of workers using hand-held vibrating tools. Cent Eur J Public Health. 1996 May;4(2):130-2.

48. Koskimies K, Pyykko I, Starck J, Inaba R. Vibration syndrome among Finnish forest workers between 1972 and 1990. Int Arch Occup Environ Health. 1992;64(4):251-6.

49. Mirbod SM, Akbar-Khanzadeh F, Onozuka M, Jamali M, Watanabe K, Inaba R, Iwata H. A four-year follow-up study on subjective symptoms and functional capacities in workers using hand-held grinders. Ind Health. 1999 Oct;37(4):415-25.

50. Musson Y, Burdorf A, van Drimmelen D. Exposure to shock and vibration and symptoms in workers using impact power tools. Ann Occup Hyg. 1989;33(1):85-96.

51. Ho ST, Yu HS. A study of neurophysiological measurements and various function tests on workers occupationally exposed to vibration. Int Arch Occup Environ Health. 1986;58(4):259-68.

52. Lundstrom R, Nilsson T, Burstrom L, Hagberg M. Exposure-response relationship between hand-arm vibration and vibrotactile perception sensitivity. Am J Ind Med. 1999 May;35(5):456-64.

53. Nilsson T, Lundstrom R. Quantitative thermal perception thresholds relative to exposure to vibration. Occup Environ Med. 2001 Jul;58(7):472-8.

54. Sanden H, Jonsson A, Wallin BG, Burstrom L, Lundstrom R, Nilsson T, Hagberg M. Nerve conduction in relation to vibration exposure - a non-positive cohort study. Journal of occupational medicine and toxicology (London, England). 2010;5:21.

55. Bovenzi M, Alessandrini B, Mancini R, Cannava MG, Centi L. A prospective study of the cold response of digital vessels in forestry workers exposed to saw vibration. Int Arch Occup Environ Health. 1998 Oct;71(7):493-8.

56. Bovenzi M, D'Agostin F, Rui F, Negro C. A longitudinal study of finger systolic blood pressure and exposure to hand-transmitted vibration. Int Arch Occup Environ Health. 2008 Apr;81(5):613-23.

57. Borenstein M, Hedges L, Higgins J, Rothstein H. Introduction to Meta-Analysis. Chichester: John Wileys & Sons Ltd; 2009.

58. Ho M, Belch JJ. Raynaud's phenomenon: state of the art 1998. Scandinavian journal of rheumatology. 1998;27(5):319-22.

59. ISO14835. Mechanical vibration and shock—cold provocation tests for the assessment of peripheral vascular function—Part 2: Measurement and evaluation of finger systolic blood pressure. Geneva: International Organization for Standardization2005b.

60. Olsen N. Diagnostic aspects of vibration-induced white finger. Int Arch Occup Environ Health. 2002 Jan;75(1-2):6-13.

61. Bovenzi M. Digital arterial responsiveness to cold in healthy men, vibration white finger and primary Raynaud's phenomenon. Scand J Work Environ Health. 1993 Aug;19(4):271-6.

62. Griffin MJ, Bovenzi M. The diagnosis of disorders caused by hand-transmitted vibration: Southampton Workshop 2000. Int Arch Occup Environ Health. 2002 Jan;75(1-2):1-5.

63. ISO14835-1. Mechanical vibration and shock—cold provocation tests for the assessment of peripheral vascular function—Part 1: Measurement and evaluation of finger skin temperature. Geneva: International Organization for Standardization,2005a.

64. Andreeva-Galanina YT. Vibration and its significance in labour. Hygine, Leningrad. 1956.

65. Drogichina EA, Metlina NB. [On the clinical picture of vibration sickness]. Klinicheskaia meditsina. 1959 Sep;37:104-10.

66. Drogichina EA, Metlina NB. A contribution to the vibration disease classification. Professionalnye Zabolevanija. 1967;12(5):27-31.

67. Taylor W, Pelmear P. Vibration White Finger in Industry. London: Academic Press; 1975.

68. Taylor W, Pelmear PL. The hand-arm vibration syndrome: an update. British journal of industrial medicine. 1990;47:577-9.

69. Gemne G, Pyykko I, Taylor W, Pelmear PL. The Stockholm Workshop scale for the classification of cold-induced Raynaud's phenomenon in the hand-arm vibration syndrome (revision of the Taylor-Pelmear scale). Scand J Work Environ Health. 1987 Aug;13(4):275-8.

70. Brammer AJ, Taylor W, Lundborg G. Sensorineural stages of the hand-arm vibration syndrome. Scand J Work Environ Health. 1987 Aug;13(4):279-83.

71. Griffin MJ. Handbook of Human Vibration. London: Academic Press; 1990.

72. Cooke JP, Marshall JM. Mechanisms of Raynaud's disease. Vasc Med. 2005 Nov;10(4):293-307.

73. Turton EP, Kent PJ, Kester RC. The aetiology of Raynaud's phenomenon. Cardiovascular surgery. 1998 Oct;6(5):431-40.

74. Brand FN, Larson MG, Kannel WB, McGuirk JM. The occurrence of Raynaud's phenomenon in a general population: the Framingham Study. Vasc Med. 1997 Nov;2(4):296-301.

75. Cherniack M, Clive J, Seidner A. Vibration exposure, smoking, and vascular dysfunction. Occup Environ Med. 2000 May;57(5):341-7.

76. Ekenvall L, Lindblad LE. Effect of tobacco use on vibration white finger disease. Journal of occupational medicine : official publication of the Industrial Medical Association. 1989 Jan;31(1):13-6.

77. Petersen R, Andersen M, Mikkelsen S, Nielsen SL. Prognosis of vibration induced white finger: a follow up study. Occup Environ Med. 1995 Feb;52(2):110-5.

78. Bovenzi M. A follow up study of vascular disorders in vibration-exposed forestry workers. Int Arch Occup Environ Health. 2008 Feb;81(4):401-8.

79. Bovenzi M, Giannini F, Rossi S. Vibration-induced multifocal neuropathy in forestry workers: electrophysiological findings in relation to vibration exposure and finger circulation. Int Arch Occup Environ Health. 2000 Nov;73(8):519-27.

80. Bovenzi M. A prospective cohort study of exposure-response relationship for vibration-induced white finger. Occup Environ Med. 2010 Jan;67(1):38-46.

81. Bovenzi M, Franzinelli A, Mancini R, Cannava MG, Maiorano M, Ceccarelli F. Dose-response relation for vascular disorders induced by vibration in the fingers of forestry workers. Occup Environ Med. 1995 Nov;52(11):722-30.

82. Bovenzi M. A longitudinal study of vibration white finger, cold response of digital arteries, and measures of daily vibration exposure. Int Arch Occup Environ Health. 2010 Mar;83(3):259-72.

83. Mirbod SM, Yoshida H, Nagata C, Inaba R, Komura Y, Iwata H. Hand-arm vibration syndrome and its prevalence in the present status of private forestry enterprises in Japan. Int Arch Occup Environ Health. 1992;64(2):93-9.

84. Bovenzi M, Pinto I, Picciolo F, Mauro M, Ronchese F. Frequency weightings of hand-transmitted vibration for predicting vibration-induced white finger. Scand J Work Environ Health. 2011 May;37(3):244-52.

85. Hagberg M, Burstrom L, Lundstrom R, Nilsson T. Incidence of Raynaud's phenomenon in relation to hand-arm vibration exposure among male workers at an engineering plant a cohort study. Journal of occupational medicine and toxicology (London, England). 2008;3:13.

86. Bovenzi M, Franzinelli A, Strambi F. Prevalence of vibration-induced white finger and assessment of vibration exposure among travertine workers in Italy. Int Arch Occup Environ Health. 1988;61(1-2):25-34.

87. Bovenzi M, Giansante C, Fiorito A, Calabrese S. Relation of haemostatic function, neurovascular impairment, and vibration exposure in workers with different stages of vibration induced white finger. Br J Ind Med. 1985 Apr;42(4):253-9.

88. Bovenzi M. Hand-arm vibration syndrome and dose-response relation for vibration induced white finger among quarry drillers and stonecarvers. Italian Study Group on Physical Hazards in the Stone Industry. Occup Environ Med. 1994 Sep;51(9):603-11.

89. Nilsson T, Burstrom L, Hagberg M. Risk assessment of vibration exposure and white fingers among platers. Int Arch Occup Environ Health. 1989;61(7):473-81.

90. Futatsuka M, Shono M, Sakakibara H, Quoc Quan P. Hand arm vibration syndrome among quarry workers in Vietnam. J Occup Health. 2005 Mar;47(2):165-70.

91. Jang JY, Kim S, Park SK, Roh J, Lee TY, Youn JT. Quantitative exposure assessment for shipyard workers exposed to hand-transmitted vibration from a variety of vibration tools. AIHA J (Fairfax, Va). 2002 May-Jun;63(3):305-10.

92. Yamada S, Sakakibara H, Futatsuka M. Vibration dose, disturbance stage, and examination results and subjective symptoms in vibration syndrome. Nagoya J Med Sci. 1995 Mar;58(1-2):1-12.

93. Letz R, Cherniack MG, Gerr F, Hershman D, Pace P. A cross sectional epidemiological survey of shipyard workers exposed to hand-arm vibration. Br J Ind Med. 1992 Jan;49(1):53-62.

94. Chatterjee DS, Petrie A, Taylor W. Prevalence of vibration-induced white finger in fluorspar mines in Weardale. Br J Ind Med. 1978 Aug;35(3):208-18.

95. Brubaker R, MacKenzie C, Hutton S. A study of vibration white finger disease among rock drillers. J Low Freq Noise Vibration. 1985;4:66-80.

96. Burdorf A, Monster A. Exposure to vibration and self-reported health complaints of riveters in the aircraft industry. Ann Occup Hyg. 1991 Jun;35(3):287-98.

97. Cherniack M, Morse TF, Brammer AJ, Lundstrom R, Meyer JD, Nilsson T, Peterson D, Toppila E, Warren N, Fu R, Bruneau H, Croteau M. Vibration exposure and disease in a shipyard: a 13-year revisit. Am J Ind Med. 2004 Jun;45(6):500-12.

98. Virokannas H, Anttonen H, Niskanen J. Vibration syndrome in railway track maintenance workers. Cent Eur J Public Health. 1995;3 Suppl:109-12.

99. Bovenzi L, Petronio M, DiMarino F. Epidemiological survey of shipyard workers exposed to hand-arm vibration. International Archives of Occupational and Environmental Health. [doi: 10.1016/0022-460X(81)90483-1]. 1980;46:251–66.

100. Tominaga Y. Vibration exposure and symptoms in postal carriers using motorbikes. Nagoya J Med Sci. 1994 May;57 Suppl:235-9.

101. Bovenzi M, Ronchese F, Mauro M. A longitudinal study of peripheral sensory function in vibration-exposed workers. Int Arch Occup Environ Health. 2011 Mar;84(3):325-34.

102. Edlund M, Burstrom L, Gerhardsson L, Lundstrom R, Nilsson T, Sanden H, Hagberg M. A prospective cohort study investigating an exposure-response relationship among vibration-exposed male workers with numbness of the hands. Scand J Work Environ Health. 2013 Sep 25.

103. Malchaire J, Piette A, Cock N. Associations between hand-wrist musculoskeletal and sensorineural complaints and biomechanical and vibration work constraints. Ann Occup Hyg. 2001 Aug;45(6):479-91.

104. Lundborg G. Nerve injury and repair: Regeneration, Reconstruction and cortical remodeling. 2nd ed. Edinburgh; New York: Churchill Livingstone; 2004.

105. Ibrahim I, Khan WS, Goddard N, Smitham P. Carpal tunnel syndrome: a review of the recent literature. The open orthopaedics journal. 2012;6:69-76.

106. Descatha A, Dale AM, Franzblau A, Coomes J, Evanoff B. Comparison of research case definitions for carpal tunnel syndrome. Scand J Work Environ Health. 2011 Jul;37(4):298-306.

107. Rempel D, Evanoff B, Amadio PC, de Krom M, Franklin G, Franzblau A, Gray R, Gerr F, Hagberg M, Hales T, Katz JN, Pransky G. Consensus criteria for the classification of carpal tunnel syndrome in epidemiologic studies. American journal of public health. [Consensus Development Conference Research Support, Non-U.S. Gov't Review]. 1998 Oct;88(10):1447-51.

108. Barcenilla A, March LM, Chen JS, Sambrook PN. Carpal tunnel syndrome and its relationship to occupation: a meta-analysis. Rheumatology (Oxford). [Meta-Analysis]. 2012 Feb;51(2):250-61.

109. Harris-Adamson C, Eisen EA, Dale AM, Evanoff B, Hegmann KT, Thiese MS, Kapellusch JM, Garg A, Burt S, Bao S, Silverstein B, Gerr F, Merlino L, Rempel D. Personal and workplace psychosocial risk factors for carpal tunnel syndrome: a pooled study cohort. Occup Environ Med. [Research Support, U.S. Gov't, P.H.S.]. 2013 Aug;70(8):529-37.

110. Pourmemari MH, Viikari-Juntura E, Shiri R. Smoking and carpal tunnel syndrome: A meta-analysis. Muscle & nerve. 2013 Jun 12.

111. Hartmann P, Mohokum M, Schlattmann P. The association of Raynaud's syndrome with carpal tunnel syndrome: a meta-analysis. Rheumatology international. 2012 Mar;32(3):569-74.

112. Bovenzi M. Epidemiological evidence for new frequency weightings of hand-transmitted vibration. Ind Health. 2012;50(5):377-87.

113. Griffin MJ. Frequency-dependence of psychophysical and physiological responses to hand-transmitted vibration. Ind Health. 2012;50(5):354-69.

114. Krajnak K, Riley DA, Wu J, McDowell T, Welcome DE, Xu XS, Dong RG. Frequency-dependent effects of vibration on physiological systems: experiments with animals and other human surrogates. Ind Health. 2012;50(5):343-53.

115. Rothstein H, Sutton A, Borenstein M. Publication bias in meta.analysis. Prevention, Assessment and Adjustments. Chichester: John Wiley & Sons Ltd; 2005.

116. Directive 2002/44/EC. Directive 2002/44/EC of the European parliament and of the Council of 25 June 2002 on the minimum health and safety requirements regarding the exposure of workers to the risks rising from physical agents (vibration) (sixteenth individual Directive within the meaning of Article 16(1) of Directive 89/391/EEC). Off J Europe Communities. 2002;L177(13-19).

117. AFS 2005:15. Föreskrift om Vibrationer. Stockholm, Sweden: Arbetsmiljöverket2005.

# Redaktörernas slutord

Hälsorisker förknippade med handöverförda vibrationer beskrevs redan i början av förra seklet. Alice Hamilton beskrev 1918 hur arbetare i ett kalkbrott i Indiana, USA utveckade en ovanlig sjukdom med stelhet och vitnade fingrar (Hamilton 1918). Sextio år senare, 1978, genomfördes en studie i samma kalkbrott och då hade 80 % av stenarbetarna vita fingrar och känselbortfall (Taylor 1984). Exponering för handöverförda vibrationer är fortfarande en vanlig exponering i arbetslivet, och skador av vibrationer är också vanliga. Dessutom, har vi sedan Alice Hamiltons undersökning, blivit varse att hand-överförda vibrationer även ger upphov till nervpåverkan och värktillstånd. Dessa skador kan med få undantag botas, och kan leda till nedsatt arbetsförmåga och livslånga svåra besvär.

Målsättningen med denna översikt är att bedöma i vilken grad exponering för handöverförda vibrationer i arbetslivet påverkar risken för vita fingrar (Raynaud's fenomen), neurosensorisk skada (känselnedsättning, värk och nedsatt kraft) och karpaltunnelsyndrom baserat på en kritisk och systematisk genomgång av litteraturen.

Författarna, liksom tidigare kunskap, visar tydligt att exponering för hand-överförda vibrationer ökar risken för vita fingrar, och att risken ökar med ökande exponering. Till exempel framgår att en av tio (10 %) utvecklar vita fingrar om de dagligen, under tio år, arbetar med ett verktyg som ger en exponering på 5 $m/s^2$ (figur 24).

Vita fingrar är köldutlösta och därför ovanligt i varma länder. Det finns bara någon enstaka studie från tropiska eller subtropiska områden. Sammanfattningsvis bekräftar den genomförda systematiska analysen av litteraturen, att risken är hög att drabbas av vita fingrar om man arbetar med vibrerande verktyg under flera år vid nivåer kring dagens gränsvärde.

Handöverförda vibrationer kan även ge upphov till neurosensorisk skada, vilket är en kunskap som växt fram under senare decennier. Författarna visar att risken att utveckla sådan skada är mycket hög efter exponering för handöverförda vibrationer. Förekomsten av neurosensorisk skada var mellan 7 % och 79 % i de sammanställda studierna med ett genomsnitt på 39 %. Tyvärr är kunskapen otillräcklig för att säkert kunna säga risken vid en given nivå och tid för en exponering, men det är uppenbart att risken är oacceptabelt hög i många fall.

Att exponering för handöverförda vibrationer ökar risken för karpaltunnel-syndrom, det vill säga skada på medianusnerven i handleden beskrevs redan på 1940-talet av Alice Hamilton (Hamilton 1943). Författarna bekräftar detta i sin litteraturgenomgång, men det saknas studier för att skapa en säker dos-respons modell.

Arbete med vibrerande verktyg är vanligt. Enligt senaste arbetsmiljöunder-sökningarna arbetar 3 % av kvinnorna och 14 % av männen minst en fjärdedel av arbetsdagen med handhållna vibrerande verktyg (totalt cirka 400 000 personer). Den utbredda användningen förklaras av att vibrerande verktyg underlättar arbetet

i många fall. Det är dock en teknologisk utmaning att behålla produktiviteten som de vibrerande verktygen ger möjlighet till, och samtidigt eliminera hälsoriskerna.

Detta dokument visar på ett mycket övertygande sätt att exponering för hand-överförda vibrationer orsakar allvarlig ohälsa hos de som arbetar med verktyg som till exempel huggmejslar, bilmaskiner, mutterdragare, borrmaskiner eller slip-maskiner. Det är också vanligt att skada uppträder redan vi nivåer kring gräns-värdet, redan efter att man arbetat kortare tid än 10 år. De beskrivna överriskerna skulle inte accepteras i andra delar av arbetslivet.

Vi menar att arbetslivets aktörer (myndigheter, arbetsgivare, fackföreningar) tydligare behöver reagera på de höga hälsoriskerna med vibrerande verktyg och vi hoppas att denna kunskapsöversikt ska vara ett incitament till detta. Det finns i dagsläget existerande kunskap om vilka maskiner som vibrerar minst och gällande förordning kräver att arbetsgivaren skall göra en riskvärdering på den enskilda arbetsplatsen (AFS 2005:15).

Vi menare att existerande kunskap om hälsorisker dock tillämpas i alltför låg utsträckning. Det finns ett behov av forskning om mekanismer för effektiv implementering och för att utveckla metoder för effektiv implementering av befintlig kunskap (Torén och Sterner, 2003, MacEachen 2016).

Göteborg, Lund och Umeå, februari 2016
Kjell Torén
Maria Albin
Bengt Järvholm

## Referenser


Arbetsmiljöverkets författningssamling. Vibrationer. AFS 2005:15

Hamilton A. A study of spastic anemia in the hands of stonecutters. Washington: Government Printing Office, 1918 (Bull US Bureau of Labor Statistics No 236: Ind Accidents and Hygiene Series, No 191:53-66.

Hamilton A. Exploring the dangerous trades – An autobiography of Alice Hamilton. M.D. Boston: Little Brown, 1943.

MacEachen E, Kosny A, Ståhl C, O'Hagen F, Redgrift L, Sanford S, Tompa E, Mahood Q. Systematic review of qualitative literature on occupational health and safety legislation and regulatory enforcement planning and implementation. Scand J Work Environ Health 2016;42:3-16.

Taylor W, Wasserman D, Behrens V, Reynolds D, Samueloff S. Effect of the air hammer on the hands of stonecutters. The limestone quarries of Bedford, Indiana, revisited. Br J Ind Med 1984;41:289-295.

Torén K, Sterner T. How to promote prevention – economic incentives or legal regulations or both? Scand J Work Environ Health 2003;29:239-245.

# Appendix

# Bilaga 1. Använd söksträng i litteraturdatabasen

Söksträngen bygger på sökorden *”Exponering och Sjukdom/Symtom”*. Dessa sökord har sedan specificerats och söksträngen som konstruerats fick följande utseende:

Search (((((((("vibration"[MeSH Terms] OR vibration[Text Word])) OR Vibrations) OR Vibration adj2 expo*) OR Segmental adj2 vibration*)) AND ((((((((((((("hand-arm vibration syndrome"[MeSH Terms] OR hand arm vibration syndrome[Text Word])) OR ("hand-arm vibration syndrome"[MeSH Terms] OR hand arm vibration syndromes[Text Word])) OR "hand-arm vibration syndrome"[MeSH Terms]) OR ("peripheral vascular diseases"[MeSH Terms] OR peripheral vascular disease[Text Word])) OR peripheral vascular diseases) OR arterial disease peripheral) OR ("raynaud disease"[MeSH Terms] OR raynaud disease[Text Word])) OR ("raynaud disease"[MeSH Terms] OR raynaud phenomenon[Text Word])) OR ("raynaud disease"[MeSH Terms] OR raynaud s disease[Text Word])) OR vibration syndrome) OR white adj2 finge*) OR vibration adj2 white) OR Raynaud*))) OR ((((((("vibration"[MeSH Terms] OR vibration[Text Word])) OR Vibrations) OR Vibration adj2 expo*) OR Segmental adj2 vibration*)) AND ((((nerve) OR ("peripheral nervous system diseases"[MeSH Terms] OR peripheral neuropathies[Text Word])) OR ("nerve compression syndromes"[MeSH Terms] OR nerve compression syndromes[Text Word])) OR ("carpal tunnel syndrome"[MeSH Terms] OR carpal tunnel syndrome[Text Word]))))

# Bilaga 2. Använda kvalitetskriterier

**Tabell.** Kriterier för kvalitetsgranskning av studierna

| **Raynaud's fenomen** | | |
|---|---|---|
| **Kriterium** | **Alternativ** | **Poäng** |
| Subjektiv symptom-beskrivning | Läkaranamnes<br>Anamnes alt färgbild<br>Enkät<br>Saknas | 3<br>2<br>1<br>0 |
| Klinisk undersökning | Läkarundersökning<br>Saknas | 2<br>0 |
| Objektiv undersökning | COP, FSBP<br>Uppvärmning<br>Saknas | 2<br>1<br>0 |
| Kontroll av diagnostiska metoder | Metod-, individ-, miljö<br>Saknas | 1<br>0 |
| Kontroll för diff. diagnos alternativt orsak | Läkar-u.s., lab screening, medicin<br>Saknas | 1<br>0 |
| Stadium klassificerat | Om stadieindelat<br>Saknas | 2<br>0 |

| **Neurosensoriska symtom** | | |
|---|---|---|
| **Kriterium** | **Alternativ** | **Poäng** |
| Subjektiv symptombeskrivning | Läkaranamnes<br>Anamnes<br>Enkät<br>Saknas | 3<br>2<br>1<br>0 |
| Klinisk undersökning | Läkarundersökning<br>Saknas | 2<br>0 |
| Objektiv undersökning | Morfologisk eller Elektrodiagnostisk<br>Saknas | 2<br>0 |
| Semi-objektiva undersökningar | QST: vibration, temperatur, annan<br>Saknas | 2<br>0 |
| Kontroll av diagnostiska metoder | Metod-, individ-, miljö<br>Saknas | 1<br>0 |
| Kontroll av diff. diagnos, eller alt. orsak | Läkar-u.s., lab screening (ej mono- eller poly n.p.)<br>Saknas | 1<br>0 |
| Stadium klassificerat | Om stadium indelat<br>Saknas | 2<br>0 |

## Karpaltunnelsyndrom

| Kriterium | Alternativ | Poäng |
|---|---|---|
| Subjektiv symptom-beskrivning | Läkaranamnes<br>Anamnes<br>Enkät<br>Saknas | 3<br>2<br>1<br>0 |
| Klinisk undersökning | Läkar-u.s. Phalen, Tinell<br>Saknas | 2<br>0 |
| Objektiv undersökning | Morfologi, eller Elektrodiagnostik<br>Saknas | 4<br>0 |
| Kontroll av diagnostiska metoder | Metod-, individ-, miljö<br>Saknas | 1<br>0 |
| Kontroll för diff. diagnos alt. orsak | Läkar-u.s., lab screening (ej polyneuropati)<br>Saknas | 1<br>0 |
| Stadium klassificerat | Om stadium indelat<br>Saknas | 2<br>0 |

## Exponering

| Kriterium | Alternativ | Poäng |
|---|---|---|
| Aktuell exponering nivå ($m/s^2$) | Objektiva mätningar<br>Subjektiv skattning<br>Uppgift saknas | 2<br>1<br>0 |
| Aktuell exponeringstid (tim/dag) | Objektiva mätningar<br>Subjektiv skattning<br>Uppgift saknas | 2<br>1<br>0 |
| Uppgifter om tidigare acceleration | Objektiva mätningar<br>Subjektiv skattning<br>Uppgift saknas | 2<br>1<br>0 |
| Uppgifter om tidigare exponeringstid (år) | Objektiva mätningar<br>Subjektiv skattning<br>Uppgift saknas | 2<br>1<br>0 |
| Uppgifter om tidigare exponeringstid (tim/dag) | Objektiva mätningar<br>Subjektiv skattning<br>Uppgift saknas | 2<br>1<br>0 |

## Metod

| Kriterium | Alternativ | Poäng |
|---|---|---|
| Studie design | RCT<br>Kohort<br>Fall-kontroll<br>Tvärsnitt | 8<br>6<br>4<br>2 |
| Deltagande frekvens | Deltagande frekvens högre än 70% alternativt bortfall vid uppföljning mindre än 30%<br>Ej uppfyllt | 2<br>0 |
| Kontroll för individuella störfaktorer i den statisiska analysen | Ja<br>Nej | 2<br>0 |

# Bilaga 3. Störningar och faktorer förknippade med sekundärt Raynauds fenomen

Nedanstående tabell visar exempel på faktorer som förknippats med Raynauds fenomen.

**Autoimmuna och reumatiska sjukdomar**

Sklerodermi
SLE
Reumatoid artrit
Dermatomyocit
Polymyosit
Mixed connective tissue disease
Vaskulit
Reumatoid artrit
CREST syndrom
Sjögrens syndrom
Tyreoidit
Primär biliär cirros
Vaskuliter (polyarteritis nodosa, polymyosit, dermatomyosit, trombangitis obliterans, polymyalgia rheumatica arterica)

**Blodsjukdomar / onkologiska sjukdomar**

Paraneoplastiska syndrom
Kryoglobulinemi
Kryoproteinemi
Köldhaemagluteinemi
Paraproteinemi
Polycytemi
POEMS syndrom*
Makroglobulinemi
Polycytemi
Kryofibrinogenemi
Köldaggluteinemi efter Mycoplasmainfektion
Anemi
Leukemi

**Endokrina sjukdomar**

Hypotyreos

**Vaskulära / obstruktiva kärlsjukdomar**

Neurovaskulärt thoraxapertursyndrom
Emboli
Vaskulit
Prinzmetals angina
Ateroskleros
Thrombangitis obliterans

**Neurologiska sjukdomar**

Karpaltunnelsyndrom
Migrän

**Miljöpåverkan**

Förfrysning
Kärlskada

**Läkemedel / toxiner**

Sympaticomimetiska läkemedel
Kemoterapeutiska läkemedel
Interferoner
Nikotin
Kokain
Ergotpreparat
Betaadrenerga blockerare
Antikonceptionsmedel
Cytostatika (metysergid, bleomycin, vinblastin)
Klonidin
Litium
Metylamfetamin

**Yrkesbetingade**

Vibrationsskador
Polyvinylklorid
Arsenik
Bly
* POEMS: polyneuropathy, organomegaly, endocrinopathy, monoclonal gammopathy, and skin changes.


1. Bakst R, Merola JF, Franks AG, Jr., Sanchez M. Raynaud's phenomenon: pathogenesis and management. Journal of the American Academy of Dermatology. 2008 Oct;59(4):633-53.
2. Block JA, Sequeira W. Raynaud's phenomenon. Lancet. 2001 Jun 23;357(9273):2042-8.

# Bilaga 4. Störningar och faktorer förknippade med nervskada (neuropati)

**Systemsjukdom som kan vara associerad med neuropati**

*Vanliga*
Diabetes mellitus
Kritisk sjukdom (sepsis)
Cancer (sent i sjukdomsförloppet samt som en följd av kemoterapi)

*Mindre vanliga*
Uremi
Vitaminbrist (utom B12)
Vitamin B12-brist
Kronisk leversjukdom
Malabsorption (glutenintolerans, celiaki)
Carcinoma (sensorimotor)
Carcinoma (demyeliniserande)
HIV-infektion
Borrelia
Lymfom, inklusive Hodgkins
Myelom
Benign monoklonal gammopati
IgA
IgG
IgM

*Sällsynta*
Porfyri (fyra typer)
Hypoglykemi
Primär biliär cirros
Primär systemisk amyloidos
Hypotyreos
Kronisk obstruktiv lungsjukdom
Akromegali
Cancer (sensorisk)
Polycytemia vera
Kryoglobulinemia

*Läkemedel*
Amiodaron
Aurotioglukos
Cisplatin
Dapson
Disulfiram
Hydralazin
Isoniazid
Leflunomid

Linezolid
Metronidazol
Misonidazol
Nitrofurantoin
Nukleosidanaloger (ddC, ddl, d4T)
Oxaliplatin
Fenytoin
Pyridoxin
Suramin
Taxol
Vinkristin

*Yrkesexponeringar / Toxiner*
Akrylamid
Arsenik
Difteri toxin
Gamma-di-keton hexa-carbons
Oorganiskt bly
Organiska fosforföreningar
Tallium
Bly
Kvicksilver
Litium
Guld
n-Hexan
Koldisulfid
Kolmonoxid
Etylenoxid
Organiska fosfater

De vanligaste bakomliggande orsakerna till perifer polyneuropati är diabetes, behandling av malign cancersjukdom och överkonsumtion av alkohol. Bland övriga orsaker till polyneuropati ingår bland annat, övriga endokrina sjukdomar och systemsjukdomar, infektioner, bristtillstånd, immunopatier och ärftliga tillstånd samt exogena intoxikationer. Ovanstående tabell visar detaljerade exempel på faktorer som förknippats med neuropati. Trots omfattande utredningar förblir dock ca. en fjärdedel av alla polyneuropatier klassade som idiopatisk det vill säga utan känd orsak (2).


1. Asbury A. Approach to the patient with peripheral neuropathy. In: Hauser S, editor. Harrisons Neurology in clinical medicine. San Francisco: McGraw-Hill; 2006. p. 491-508.
2. Rutkove S. Overview of polyneuropathy. UpToDate2014 [updated May 12 2014].

# Bilaga 5. Störningar och faktorer förknippade med karpaltunnelsyndrom

Nedanstående tabell visar exempel på faktorer som förknippats med karpaltunnelsyndrom.

**Allmänna riskfaktorer**

Fetma
Kvinnligt kön
Graviditet
Diabetes
Reumatoid artrit
Hypotyreoidism
Bindvävssjukdomar
N. medianus mononeuropati
Genetiska anlag
Tamoxifenbehandling
Infektioner

**Arbetsplatsfaktorer som har föreslagits kunna orsaka eller förvärra KTS**

Tryck över hand, handled
Långvarig handledsposition i extension eller flexion
Upprepad långvarig extension och flexion i handleden
Arbete med vibrerande verktyg
Användning av händerna i kall temperatur

1. Rosenbaum R, Ochoa J. Carpal tunnel syndrome and other disorders of the median nerve. Second ed. Boston: Butterworth Heinemann; 2002.
2. Palmer KT. Carpal tunnel syndrome: the role of occupational factors. Best practice & research Clinical rheumatology. 2011 Feb;25(1):15-29.
3. Kothari M. Etiology of carpal tunnel syndrome. UpToDate2014 [updated Oct 24 2014].

# Senaste utgåvorna i den vetenskapliga skriftserien ARBETE OCH HÄLSA

**2011;45(4).** The Nordic Expert Group for Criteria Documentation of Health Risks from Chemicals and the Dutch Expert Committee on Occupational Safety. 144. Endotoxins.

**2011;45(5). M Albin, J Alkan-Olsson, M Bohgard, K Jakobsson, B Karlson, P Lundqvist, M Ottosson, F Rassner, M Svensson and H Tinnerberg. (Ed.)** 55th Nordic Work Environment Meeting.The Work Environment – Impact of Technological, Social and Climate Change.

**2011;45(6). J Montelius (Ed.) Swedish Criteria Group for Occupational Standards.** Scientific Basis for Swedish Occupational Standards XXXI.

**2011;45(7).** The Nordic Expert Group for Criteria Documentation of Health Risks from Chemicals and the Dutch Expert Committee on Occupational Safety. 145. Aluminium and aluminium compounds.

**2012;46(1). B Lindell.** The Nordic Expert Group for Criteria Documentation of Health Risks from Chemicals. 146. Polychlorinated biphenyls. (PCBs)

**2012:46(2). K Torén, M Albin och B Järvholm.** Systematiska kunskapsöversikter; 2. Exponering för helkroppsvibrationer och uppkomst av ländryggssjuklighet.

**2012:46(3). G Sjögren Lindquist och E Wadensjö.** Kunskapsöversikt kring samhällsekonomiska kostnader för arbetsskador.

**2012;46(4). C Mellner, G Aronsson och G Kecklund.** Segmentering och integrering – om mäns och kvinnors gränssättningsstrategier i högkvalificerat arbete.

**2012;46(5). T Muhonen.** Stress, coping och hälsa under kvinnliga chefers och specialisters karriärer.

**2012;46(6). J Montelius (Ed.) Kriteriegruppen för hygieniska gränsvärden.** Vetenskapligt Underlag för Hygieniska Gränsvärden 32.

**2012;46(7). H Stockmann-Juvala.** The Nordic Expert Group for Criteria Documentation of Health Risks from Chemicals. 147. Carbon monoxide.

**2013;47(1). I Lundberg, P Allebeck, Y Forsell och P Westerholm.** Systematiska kunskapsöversikter; 3. Kan arbetsvillkor orsaka depressionstillstånd? En systematisk översikt över longitudinella studier i den vetenskapliga litteraturen 1998-2012.

**2013;47(2). K Elgstrand and E Vingård (Ed.)** Occupational Safety and Health in Mining. Anthology on the situation in 16 mining countries.

**2013;47(3). A Knutsson och A Kempe.** Systematiska kunskapsöversikter; 4. Diabetes och arbete.

**2013;47(4). K Jakobsson och P Gustavsson.** Systematiska kunskapsöversikter; 5. Arbetsmiljöexponeringar och stroke – en kritisk granskning av evidens för samband mellan exponeringar i arbetsmiljön och stroke.

**2013;47(5). M Hedmer, M Kåredal, P Gustavsson and J Rissler.** The Nordic Expert Group for Criteria Documentation of Health Risks from Chemicals. 148. Carbon nanotubes.

**2013;47(6). J Montelius (Ed.) Swedish Criteria Group for Occupational Standards.** Scientific Basis for Swedish Occupational Standards XXXII.

**2013;47(7). C Håkansta.** Between Science and Politics - Swedish work environment research in a historical perspective.

**2013;47(8). J Montelius (Ed.) Kriteriegruppen för hygieniska gränsvärden.** Vetenskapligt Underlag för Hygieniska Gränsvärden 33.

**2014;48(1). L-G Gunnarsson och L Bodin.** Systematiska kunskapsöversikter; 6. Epidemologiskt påvisade samband mellan Parkinsons sjukdom och faktorer i arbetsmiljön.

**2014;48(2). L-G Gunnarsson och L Bodin.** Systematiska kunskapsöversikter; 7. Epidemiologiskt påvisade samband mellan Amyotrofisk Lateral Skleros (ALS) och faktorer i arbetsmiljön.

**2014;48(3). J Montelius (Ed.) Swedish Criteria Group for Occupational Standards.** Scientific Basis for Swedish Occupational Standards XXXIII.

**2015;49(1). J Montelius (Ed.) Kriteriegruppen för hygieniska gränsvärden.** Vetenskapligt underlag för hygieniska gränsvärden 34.

**2015;49(2). G Aronsson och U Lundberg.** Interventioner för återgång i arbete vid sjukskrivning. En systematisk kunskapsöversikt av metaanalyser med inriktining på muskuloskeleterala och psykiska besvär.

**2015;49(3). L-G Gunnarsson och L Bodin.** Systematiska kunskapsöversikter; 8. Epidemiologiskt undersökta samband mellan Alzheimers sjukdom och faktorer i arbetsmiljön
